# Phthalates and bisphenols early-life exposure, and childhood allergic conditions: a pooled analysis of cohort studies

**DOI:** 10.1038/s41370-025-00790-2

**Published:** 2025-07-03

**Authors:** Thomas Boissiere-O’Neill, Nina Lazarevic, Peter D. Sly, Anne-Louise Ponsonby, Aimin Chen, Meghan B. Azad, Joseph M. Braun, Jeffrey R. Brook, David Burgner, Bruce P. Lanphear, Theo J. Moraes, Richard Saffery, Padmaja Subbarao, Stuart E. Turvey, Kimberly Yolton, CHILD investigator group, Meghan B. Azad, Theo J. Moraes, Padmaja Subbarao, Stuart E. Turvey, Elinor Simons, Piushkumar J. Mandhane, BIS investigator group, Peter D. Sly, Anne-Louise Ponsonby, David Burgner, Richard Saffery, Peter Vuillermin, Mimi L. K. Tang, Fiona Collier, Leonard Harrison, Sarath Ranganathan, Toby Mansell, Martin O’Hely, Lawrence Gray, Dwan Vilcins

**Affiliations:** 1 The University of Queensland, Child Health Research Centre, The Children’s Health and Environment Program, Brisbane, QLD Australia; 2 National Centre for Epidemiology and Population Health, Australian National University, Canberra, ACT Australia; 3 Florey Institute of Neuroscience and Mental Health, University of Melbourne, Melbourne, VIC Australia; 4 Department of Biostatistics, Epidemiology and Informatics, Perelman School of Medicine, University of Pennsylvania, Philadelphia, PA USA; 5 Children’s Hospital Research Institute of Manitoba, Department of Pediatrics and Child Health, University of Manitoba, Winnipeg, MB Canada; 6 Department of Epidemiology, Brown University, Providence, RI USA; 7 Dalla Lana School of Public Health, University of Toronto, Toronto, ON Canada; 8 Murdoch Children’s Research Institute, Royal Children’s Hospital, The University of Melbourne, Melbourne, VIC Australia; 9 Faculty of Health Sciences, Simon Fraser University, Burnaby, BC Canada; 10 Department of Pediatrics, Hospital for Sick Children, University of Toronto, Toronto, ON Canada; 11 Department of Pediatrics, Child and Family Research Institute, BC Children’s Hospital, University of British Columbia, Vancouver, BC Canada; 12 Cincinnati Children’s Hospital Medical Center and the University of Cincinnati College of Medicine, Cincinnati, OH USA; 13 Department of Pediatrics, University of Alberta, Edmonton, AB Canada; 14 Deakin University, Institute for Mental and Physical Health and Clinical Translation (IMPACT), School of Medicine, Geelong, VIC Australia; 15 The Walter and Eliza Hall Institute of Medical Research, Melbourne, VIC Australia

**Keywords:** Phthalates, Bisphenols, Asthma, Allergies, Children, Pooled-analysis

## Abstract

**Background:**

Exposure to plastic additives, such as phthalates and bisphenols, has been associated with a higher risk of allergic conditions, but the evidence is inconsistent for children younger than five.

**Objective:**

To examine the association between pre- and postnatal urinary phthalates and bisphenols, and allergic conditions, and potential effect modification by sex, in pre-school children, through a pooled analysis.

**Methods:**

We pooled data from the Barwon Infant Study (Australia), the Canadian Healthy Infant Longitudinal Development Study (Canada), the Health Outcomes and Measures of the Environment (United States) and the Environmental Influences on Child Health Outcomes–wide cohorts (United States). Urinary phthalates and bisphenols were measured during pregnancy and early childhood. We estimated daily intakes from urinary concentrations, except for mono-(3-carboxypropyl) phthalate (MCPP). Outcomes, including asthma, wheeze, eczema, and rhinitis, were assessed up to five years of age through questionnaires and clinical assessments. We used generalised estimating equations for single compounds and quantile G-computation for the chemical mixtures.

**Results:**

5306 children were included. A two-fold increase in prenatal dibutyl phthalates (DBP; risk ratio [RR] = 1.08; 95% confidence interval [CI]: 1.00–1.16) and benzyl butyl phthalate (BBzP; RR = 1.06; 95%CI: 1.00–1.12) increased the risk of asthma in children under five. Prenatal MCPP levels were associated with rhinitis (RR = 1.05; 95%CI: 1.01–1.09). Postnatal BBzP levels increased the risk of wheezing (RR = 1.05; 95%CI 1.01–1.09), as well as di(2-ethylhexyl) phthalate (DEHP; RR = 1.06; 95%CI: 1.01–1.11) and MCPP (RR = 1.09; 95%CI: 1.04–1.14). These were also inversely associated with eczema. A one-quartile increase in the postnatal chemical mixture increased the risk of wheezing (RR = 1.14; 95%CI: 1.02–1.26). There was limited evidence of effect modification by sex.

**Impact:**

- Phthalates and bisphenols are widespread and may contribute to allergic conditions in children. We pooled data from 5000 children across multiple birth cohorts, suggesting that early-life exposure to these chemicals is associated with increased risks of asthma, wheezing, and rhinitis by age five. We further investigated the timing of exposure, non-linear dose-response relationships, and effect measure modification by sex. This study provides a comprehensive assessment of early-life exposure to phthalates and bisphenols and strengthens the evidence for their role in the development of childhood allergic outcomes.

## Introduction

Endocrine-disrupting chemicals (EDCs) can alter the endocrine system by interfering with hormonal action and have been associated with a variety of adverse health outcomes [1, 2]. Phthalates and bisphenols are among the most common EDCs and are ubiquitous in the population [2, 3]. High-molecular-weight phthalates (HMWPs), such as benzyl-butyl phthalate (BBzP) and di-2-ethylhexyl phthalate (DEHP), increase the flexibility and durability of plastics and are added to products such as vinyl flooring, food packaging, and children’s toys [2]. Low-molecular-weight phthalates (LMWPs), such as dimethyl phthalate (DMP), diethyl phthalate (DEP), or di-iso-butyl phthalate (DiBP), are mainly found in personal care products [2]. Finally, bisphenols, such as bisphenol A (BPA), bisphenol S (BPS), and bisphenol F (BPF), are used in the manufacture of polycarbonate plastics and epoxy resins, and are mainly found in food packaging, thermal paper, and cans [3].

Phthalates and bisphenols have been suggested to induce a T helper (Th) 2 immune response, resulting in increased susceptibility to allergic inflammation [4, 5]. Potential mechanisms are numerous but include oxidative stress, epigenetic, and estrogenic modulations [4, 5]. Given that these chemicals can interact with estrogenic pathways, their effect on childhood allergic conditions may also differ between sexes [4]. Epidemiological studies have shown that phthalates and bisphenols can increase the risk of childhood asthma, wheezing, eczema, and rhinitis [6–11]. However, systematic reviews and meta-analyses have highlighted that findings in the literature are inconsistent and that the relationship between phthalates, bisphenols and childhood allergic conditions requires further study [12–16]. For instance, on study has shown an increase in the odds of developing eczema [11], while others have contradicted these findings [10]. Such variations in the literature can be attributed to variations in the timing of exposure, latency between exposure and health effects, variations in the exposure mixture, and non-monotonic dose-response relationships [17].

The prenatal period is a critical window of susceptibility, as foetal development is highly sensitive to environmental insults, which may disrupt organogenesis and the maturation of the immune and respiratory systems [18]. Furthermore, children up to five years old are particularly vulnerable to chemical exposure due to higher exposure relative to their body weight, immature metabolic pathways, and active physiological growth [18]. However, this age group remains underrepresented in studies investigating the association between phthalates, bisphenols, and allergic conditions. Moreover, previous studies were hindered by small sample sizes, potential residual confounding issues and exposure measurement error [4]. Finally, most studies have focused on specific chemicals, disregarding the potential for a combined effect of plasticisers.

We aimed to investigate associations between early-life urinary phthalates and bisphenols and the risk of allergic conditions in preschool-aged children using a pooled analysis from three high-income countries. We also aimed to assess whether the child’s sex modifies the association between phthalates, bisphenols, and allergic conditions. Finally, we estimated the association between chemical mixtures and childhood allergic conditions.

## Methods

### Data sources

This pooled analysis draws from four data sources: the Barwon Infant Study (BIS), the Canadian Healthy Infant Longitudinal Development (CHILD) Study, the Health Outcomes and Measures of the Environment (HOME) Study, and the Environmental Influences on Child Health Outcomes (ECHO)-wide cohorts. Details on the individual cohorts, such as the inclusion/exclusion criteria, have been published elsewhere [19–22]. Briefly, BIS enrolled 1074 mother-infant pairs from Victoria, Australia, recruited between 2010 and 2013, excluding very preterm deliveries (≤32 weeks), genetic diseases, major congenital malformations, or serious illnesses [19]. Ethical approval was provided by the Barwon Health Human Research Ethics Committee (HREC 10/24), and families provided informed consent. CHILD, established between 2008 and 2012, recruited 3624 pregnant mothers from four Canadian communities, excluding mothers with moderate preterm deliveries (≤35 weeks), respiratory distress syndrome, and in vitro fertilisation, resulting in 3542 infants in the inception cohort [20]. Each recruitment centre obtained approval from local Research Ethics Boards, and each participant provided signed informed consent. The HOME Study, a longitudinal cohort from Cincinnati, Ohio, recruited women from prenatal practices between 2003 and 2006 during their 2nd trimester of gestation, with inclusion criteria related to year residence was built and maternal health conditions, resulting in 401 women–infant pairs [21]. Ethical approval was provided by the Cincinnati Children’s Hospital Medical Centre and cooperating delivery hospitals, and all participants provided written informed consent. The ECHO-wide cohorts, encompassing 69 pregnancy and paediatric cohorts across the USA and Puerto Rico, started in the 1980s and continues to enrol participants, focusing on diverse populations and harmonising data collection through the ECHO-Wide Data Collection Protocol (ECWP) approved by the Western institutional review board in 2019 [22]. ECHO data was obtained through the Data and Specimen Hub provided by the Eunice Kennedy Shriver National Institute of Child Health and Human Development, with data locked as of 31st August 2022 [23]. The ECHO-wide cohorts, with their diverse designs, objectives, and data collection protocols, included 56 cohorts (30,904 children) that consented to the ECWP [22]. Local institutional review boards approved all cohorts, and de-identified data were used for this analysis [22].

### Study participants

The study population included participants who had data on urinary phthalates or bisphenols during pregnancy or within the first five years of childhood and had at least one measure of childhood allergy within the same timeframe. We separated the study population into those with exposure measured during pregnancy (prenatal analysis) and those measured during childhood (postnatal analysis). We excluded cohorts in which more than 50% of participants had missing covariate data or those with a sample size below 30 [24]. We included nine ECHO-wide cohorts, BIS, CHILD and HOME studies. After applying the exclusion criteria, there were 3763 participants in the prenatal analysis and 1862 participants in the postnatal analysis (Fig. S1).

### Exposure characterisation

In all cohorts, urinary phthalates and bisphenols were assessed using validated methods described elsewhere [25–28] (Table 1). All methods used metabolite deconjugation before analysis by high-performance liquid chromatography-tandem mass spectrometry. Within each cohort, phthalates with a detection rate > 50% and bisphenols with a detection rate >10% were included (Table 1). These thresholds allowed us to maximise the number of participants in the analyses while maintaining sufficient variability in the exposure to identify associations, in line with previous studies [29, 30]. The limits of detection for each metabolite are shown in Table S1. To maintain methodological continuity and comparability with previous work from BIS, HOME and CHILD, we applied the same approaches to handling values below the limit of detection (LOD) that had been previously validated [31–33].

**Table 1:** Analytical exposure methods across cohorts.

Cohort	Country	Exposure timing	Mean Samples/Subject	Freezing temperature	Analytical centre	Analytical method	Urine dilution variable	Time of Day Adjustment^a^	Metabolites included
BIS^b^	Australia	36 gestational weeks	1	−80 °C	QAEHS	HPLC-MS/MS with direct injection [25]	Specific Gravity	Yes	MMP, MEP, MiBP, MnBP, MBzP, MEHHP, MEOHP, MECPP, MCPP, BPA, BPS, BPF
HOME (prenatal)^b^	USA	16 & 26 gestational weeks	2	−20 °C	CDC	SPE- HPLC-MS/MS [26, 27]	Specific Gravity	No	MEP, MiBP, MnBP, MBzP, MEHHP, MEOHP, MECPP, MCPP, BPA
HOME (postnatal)^b^	USA	1–4 years	2.8	−20 °C	CDC	SPE- HPLC-MS/MS [26, 27]	Specific Gravity	No	MEP, MiBP, MnBP, MBzP, MEHHP, MEOHP, MECPP, MCPP, BPA
AAA01^c^	USA	10–28 gestational weeks	2	−80 °C	CDC	SPE- HPLC-MS/MS [27]	Creatinine	Yes	BPA, BPS, BPF
AAG01^c^	USA	6–35 gestational weeks	2.3	−80 °C	Wadsworth Laboratory	SPE- HPLC-MS/MS [26, 27]	Creatinine	Yes	MEP, MiBP, MnBP, MBzP, MEHHP, MEOHP, MECPP, MCPP, BPA
AAM01^c^	USA	4–40 gestational weeks	2.9	−80 °C	Wadsworth Laboratory	SPE- HPLC-MS/MS [26, 27]	Specific Gravity	Yes	MMP, MEP, MiBP, MnBP, MBzP, MEHP, MEHHP, MEOHP, MECPP, MCPP, BPA, BPS, BPF
AAV01^c^	USA	21–34 gestational weeks	1	−80 °C	CDC	SPE- HPLC-MS/MS [26, 27]	Creatinine	Yes	MMP, MEP, MiBP, MnBP, MBzP, MEHP, MEHHP, MEOHP, MECPP, MCPP, BPA, BPS
AAZ01^c^	USA	17–39 gestational weeks	1.9	−80 °C	Wadsworth Laboratory	SPE- HPLC-MS/MS [26]	Specific Gravity	Yes	MMP, MEP, MiBP, MnBP, MBzP, MEHP, MEHHP, MEOHP, MECPP, MCPP
AAZ02^c^	USA	9–40 gestational weeks	1	−80 °C	Wadsworth Laboratory	SPE- HPLC-MS/MS [26]	Specific Gravity	Yes	MMP, MEP, MiBP, MnBP, MBzP, MEHP, MEHHP, MEOHP, MECPP, MCPP
ABA03^c^	USA	14–40 gestational weeks	2.2	−80 °C	CDC	SPE- HPLC-MS/MS [26, 27]	Specific Gravity	Yes	MEP, MiBP, MnBP, MBzP, MEHP, MEHHP, MEOHP, MECPP, MCPP, BPA, BPS, BPF
AFA01^c^	USA	15–19 gestational weeks	1	−80 °C	CDC	SPE- HPLC-MS/MS [26, 27]	Specific Gravity	Yes	MEP, MiBP, MnBP, MBzP, MEHP, MEHHP, MEOHP, MECPP, MCPP, BPA, BPS, BPF
AGA01^c^	USA	12–38 gestational weeks	2.3	−80 °C	CDC	SPE- HPLC-MS/MS [26, 27]	Specific Gravity	Yes	MEP, MiBP, MnBP, MBzP, MEHP, MEHHP, MEOHP, MECPP, MCPP, BPA, BPS, BPF
CHILD	Canada	3-month, 1 year, 3-year	2.4	−80 °C	AXYS Analytical Services Inc	SPE- HPLC-MS/MS [28]	Specific Gravity	No	MEP, MiBP, MnBP, MBzP, MEHP, MEHHP, MEOHP, MCPP

^a^Adjustment for time of day at urine collection.

^b^MEHP not included due to external contamination.

^c^ECHO-wide cohorts.

*BBzP* Butyl Benzyl Phthalate, *BIS* Barwon Infant Study, *BPA* Bisphenol A, *BPF* Bisphenol F, *BPS* Bisphenol S, *CDC* Centre for Disease Control and Prevention, *HPLC-MS* High-Performance Liquid Chromatography-Mass Spectrometry, *MECPP* Mono-Ethylcarboxypentyl Phthalate, *MEHP* Mono(2-ethylhexyl) Phthalate, *MEHHP* Mono-Ethylhydroxyhexyl Phthalate, *MEOHP* Mono-Ethyloxohexyl Phthalate, *MEP* Mono-Ethyl Phthalate, *MiBP* Mono-Isobutyl Phthalate, *MMP* Mono-Methyl Phthalate, *MnBP* Mono-Butyl Phthalate, *MBzP* Mono-Benzyl Phthalate, *MCPP* Mono(3-carboxypropyl) Phthalate, *QAEHS* Queensland Alliance for Environmental Health Sciences, *SPE* Solid-Phase Extraction.

#### BIS

Single spot urine samples were collected at 36 gestational weeks and analysed by the Queensland Alliance for Environmental Health Sciences [25, 29]. Measurements below the limit of detection (LOD) were imputed using LOD/√2 for phthalates and, due to lower detection rates, bisphenols were multiply imputed using a left-censored log-normal distribution with five datasets [29, 34]. The Levine-Fahey equation was used to correct for urine dilution using specific gravity: ${E}_{{Sg}}={E}_{0} \times [({{Sg}}_{{median}}-1)/({{Sg}}_{0}-1)]$, where E_sg_ is the specific gravity-corrected analyte, E_0_ is the observed analyte, Sg_median_ is the median of specific gravity values in the study sample, and Sg_0_ is the observed specific gravity value [35]. Phthalate and bisphenol measurements were weighted to account for batch effects [36], and corrected for the time of day at sampling using the residual method [37].

#### ECHO-wide cohorts

Spot urine samples were collected throughout pregnancy (4–40 gestational weeks) and analysed by the Division of Laboratory Sciences, National Centre for Environmental Health, Centres for Disease Control and Prevention (CDC), or the Wadsworth Laboratory, New York State Department of Health (Table 1) [26, 27]. The number of samples per pregnant woman ranged from one to eight, averaging two samples per mother. Values below the LOD were imputed with LOD/√2 for phthalates. Due to lower detection rates in some cohorts, bisphenols were multiply imputed using a left-censored log-normal distribution, as above. When available, we used specific gravity to standardise the sample for urine dilution, as described above. If specific gravity was unavailable for an entire cohort (*n* = 3 cohorts, 548 children), we used the Boeniger method for creatinine standardisation: ${E}_{{Cr}}={E}_{0}\times [({{Cr}}_{{median}})/({{Cr}}_{0})]$, where E_cr_ is the creatinine-corrected analyte, E_0_ is the observed analyte, Cr_median_ is the creatinine median in the study sample, and Cr_0_ is the observed creatinine value [38]. Missing urine dilution data (*n* = 120 samples, 2%) were imputed using linear regression with gestational age at sampling, maternal age, and pre-pregnancy weight. Standardisation for time of day at sampling was applied using the residual method [37].

#### CHILD

Spot urine samples were collected at three months, one year, and three years [32]. For nontoilet-trained children, urine was collected using Tegaderm plastic film and cotton pads, which were subsequently squeezed to extract urine [32]. AXYS Analytical Service analysed the samples using the Kato method [28]. We used previously described phthalate variables, standardised for specific gravity using the Levine-Fahey equation [32]. Values below the LOD were imputed with a log-normal distribution using NDexpo [32].

#### HOME

Spot urine samples were collected into polypropylene cups during pregnancy at 16 and 26 weeks and annually during childhood before analysis by the CDC [26, 27]. Surgical inserts were placed inside the diaper for non-toilet-trained infants, and urine was extracted into the specimen cups [39]. Specific gravity was used for urine dilution correction as described above [35]. Missing specific gravity data (*n* = 82 samples, 5%) were imputed using linear regression with creatinine, gestational age, maternal age, and body weight. Analytes with values below the LOD were imputed with LOD/√2 [34].

### Outcome assessment

This study assessed four health outcomes from birth to five years of age: asthma, wheezing, eczema, and rhinitis.

#### BIS

Caregivers completed a validated questionnaire at 1, 3, 6, 9, 12, 18, 24, and 48 months [40]. Asthma was assessed during the 2- and 4-year reviews based on a positive response from parents to the question, *“Has your child ever had asthma*?”. Wheeze was evaluated at each time point by asking caregivers, *“Has your child experienced wheezing or whistling from the chest since the last review?”*. Eczema was identified at all time points using the modified UK Working Party criteria [41]. This included a history of itchy skin, plus at least three of the following: a history of dry skin, a family history of allergy, a history of skin rash affecting the flexures or outer surfaces of the limbs or the head or cheeks, visible dermatitis assessed during a study visit at either 1 month, 6 months, 1 year or 4 years. Rhinitis was determined at the 2- and 4-year reviews if parents responded affirmatively to the question, “*Has your child ever had hay fever?*”

#### ECHO-wide cohorts

Outcomes were assessed using the International Study of Asthma and Allergies in Childhood (ISAAC) questionnaire [42]. All outcomes were caregiver-reported episodes since the previous visit. The timing and number of outcome measurements varied across cohorts, with some children having their outcome measured at six months only, while others had repeated measures up to eight times, spanning from birth to five years of age (Table S2). Asthma was defined as a positive response to the question, *“Has the child ever had asthma?*”; wheeze as “*Has the child ever had wheezing or whistling in the chest at any time in the past?*”; eczema as “*Has the child ever had eczema (also called atopic dermatitis)?*”; and rhinitis as “*Has the child ever had a problem with sneezing or a runny or blocked (stuffy) nose when he/she did NOT have a cold or the flu?*”.

#### CHILD

Subspecialist paediatricians assessed allergic outcomes in children aged 1, 3 and 5 years, which were classified as “definite”, “possible”, or “no” [43]. Only those with a “definite” diagnosis were considered cases to mitigate the risk of outcome misclassification. Asthma and allergic rhinitis were assessed at the three- and five-year study visits [43]. Eczema was clinically assessed at 1, 3, and 5 years, using the modified UK Working Party criteria [41]. Data on wheezing collected at 3, 6, 12, 18, 24, 36, 48, and 60 months was defined as a positive response to “*In the last X months, has your child had a wheezing noise (whistling sound) coming from his/her chest either WITH a cold or WITHOUT a cold?”*.

#### HOME

Caregivers were surveyed biannually on their child’s allergic outcomes from six months to five years of age, with a questionnaire adapted from the National Health and Nutrition Examination Survey [44]. Asthma was defined from two to five years by the question, “*Has a doctor or other health professional ever told you that [child] has asthma?*”. Wheeze was assessed at all time points with the question, “*Has [child] had wheezing or whistling in his/her chest since he/she was X months old?*”. Eczema was evaluated by asking, “*Has [child] had any symptoms of eczema since he/she was X months old?*”. Rhinitis was defined by the question, “*Since [child] was X months old, has he/she had any symptoms of hay fever or allergies, such as recurrent runny, itchy, or stuffy nose without a cold, or recurrent sneezing and itchy eyes?*”.

### Covariates

We identified potential confounders by reviewing the literature using a directed acyclic graph (Fig. S2). Most covariates were caregiver-reported, except those collected at birth, for which hospital records were used. Data harmonisation of categorical variables is shown in Table S3. To account for variability in exposure distributions, outcome prevalence and covariate distribution between cohorts, we adjusted all models for cohort membership, namely BIS, HOME, CHILD, and each of the nine ECHO-wide cohorts. In the prenatal analysis, covariates included offspring sex (male; female), maternal age at conception (years; continuous), ethnicity (Caucasian/White; Other), Marital status reported during pregnancy (Single/Not married; Other), the highest level of caregiver education reported during pregnancy (high school or under; bachelor’s degree; master or doctorate; Other), family history of asthma (yes; no), and season of birth (categorical; Spring, Summer, Autumn, Winter). Ethnicity was included to account for racial disparities in chemical exposure and prevalence of childhood allergies. We collapsed ethnicity categories given the differences in categories between cohorts (Table S3). In addition, prenatal exposure to tobacco smoke was assessed via questionnaires administered during pregnancy, while for the HOME Study, this was quantified using serum cotinine levels at 16 and 26 gestational weeks, with women exhibiting cotinine levels above the LOD (0.015 ng/ml) classified as exposed to tobacco smoke [45]. Further adjustments for the postnatal analysis were made for any breastfeeding duration (weeks, continuous), gestational age (weeks, continuous), postnatal tobacco smoke exposure (yes; no), and age at outcome assessment (years, continuous) in addition to the covariates above. Postnatal tobacco smoke exposure was determined based on caregiver-reported smoking inside the house or dwellings. In the HOME Study, child serum cotinine concentrations at each visit were used, with thresholds described by Mourino et al. [46]. The distribution of serum cotinine concentrations in HOME is shown in Fig. S3. All covariates were considered time-fixed except age at outcome assessment, which we considered time-varying.

### Statistical analysis

#### Exposure processing

To normalise biomarker concentrations for body weight and facilitate comparison with regulatory reference values, we derived estimated daily intakes (EDI) from urinary metabolites, accounting for weight at sampling, average daily urine volume, fractional excretion, and the compound-to-metabolite molecular weight ratio:

1 $${{EDI}}=\frac{{C}_{{adj}}* {UV}* {{MW}}_{{diester}}}{{FUE}* W* {{MW}}_{{metabolite}}}$$

Where C_adj_ is the standardised metabolite urinary concentration (µg/L), UV is the average daily urine volume (1.6 L during pregnancy), FUE is the metabolite fractional urinary excretion (unitless), MW is the molecular weight (g/mol), and W is the body weight at sampling (kg). In all cohorts, pre-pregnancy weight was self-reported. In BIS, weight at sampling was imputed using a linear model including pre-pregnancy weight, maternal weight at 28 weeks, birth weight, placental weight, maternal postnatal weight (4 weeks postpartum), and recalled pre-pregnancy weight gain at four years postpartum [31]. In other cohorts, it was estimated using the Institute of Medicine’s recommended weight gain during pregnancy based on pre-pregnancy BMI [47]. For postnatal exposure, UV/W was estimated from the child’s age at sampling [48]. Mono-methyl phthalate (MMP) was used to calculate dimethyl phthalate (DMP) daily intake, monoethyl phthalate (MEP) for diethyl phthalate (DEP), mono-isobutyl phthalate (MiBP) for di-isobutyl phthalate (DiBP), and mono-n-butyl phthalate (MnBP) for di-n-butyl phthalate (DnBP); we used the sum of DiBP and DnBP to derive di-butyl phthalates (DBP) daily intake. Monobenzyl phthalate (MBzP) was used to calculate benzyl butyl phthalate (BBzP). For di(2-ethylhexyl) phthalate (DEHP), we used the molar sum of mono(2-ethylhexyl) phthalate (MEHP), mono(2-ethyl-5-oxohexyl) phthalate (MEOHP), mono(2-ethyl-5-hydroxyhexyl) phthalate (MEHHP), and mono(2-ethyl-5-carboxypentyl) phthalate (MECPP). In the HOME Study, MEHP was excluded from the 1–3-year time points due to external contamination, so only DEHP oxidative metabolites were considered for DEHP calculation to ensure consistency across maternal and child samples [39]. In BIS, MEHP was excluded due to external contamination [29]. In CHILD, we lacked data on MECPP; therefore, we used MEHP, MEOHP, and MEHHP for DEHP calculation. Since Mono (3-carboxypropyl) phthalate (MCPP) is a non-specific metabolite of multiple high-molecular-weight phthalates, we kept urinary MCPP concentrations rather than calculating EDI. Due to insufficient measurements and low detection rates, we did not include DMP, bisphenol S (BPS), or bisphenol F (BPF) in the postnatal analysis.

#### Single-compound associations

We first summarised cohort characteristics using descriptive statistics. Given the right-skewed distribution of exposure variables, we applied a log2 transformation. The correlation plots of exposures are shown in Fig. S4–S5. In a complete case analysis, we estimated pairwise longitudinal associations between single compounds and repeated outcome measures. We used generalised estimating equations with an exchangeable correlation matrix to account for repeated measures to estimate longitudinal associations. A Poisson model with log link function and robust standard errors was used to estimate risk ratios (RR), associated with a twofold increase in the exposure levels and 95% confidence intervals (CI) [49]. For the prenatal analysis, the average of each compound was used when multiple samples were available. For postnatal analysis, we estimated lagged associations. Specifically, future outcomes were regressed on repeated exposures, with outcome measures aggregated over each exposure time point (i.e., 12-month exposure aligned with outcomes from 12–24 months), as previously described elsewhere (Fig. S6) [50]. We then added smoothing terms with restricted cubic splines with three knots located at each exposure’s 10th, 50th and 90th percentiles to plot dose-response curves, and calculated *p*-values for non-linearity with Wald tests on spline terms [51]. To investigate effect measure modification by sex, we added an interaction term to each model, and stratum-specific estimates were obtained using the *marginaleffects* package (RStudio, version 0.17.0) [52]. To identify windows of vulnerability during pregnancy, we used the average exposure concentration for each trimester and stratified the analysis by trimester. We opted not to correct for multiple comparisons, given that we aimed to investigate underlying patterns of associations, thereby prioritising the detection of potential signals over the control of the Type I error rate [53].

#### Sensitivity analyses

First, we repeated our analysis on urinary biomarkers instead of estimated daily intakes to assess whether the uncertainty in estimating daily intakes influenced our results. Since a pooled analysis assumes a common treatment effect across cohorts, we then conducted a random-effects meta-analysis using restricted maximum likelihood on cohort-stratified effect estimates to evaluate this assumption and examine between-cohort heterogeneity [54]. Third, assuming a missing-at-random mechanism, we imputed missing covariates (*n* = 207 children; 3.9%) with multiple imputations by chained equations, using the same variables from the main analysis, generating five datasets with 20 iterations [24]. Fourth, to mitigate potential selection bias arising from systematic differences between the subjects included in the analysis and those excluded from the inception cohorts due to missing exposure or outcome data, we used stabilised inverse probability weights (IPWs) [55, 56]. The weights were based on the same covariates used in the main models. Fifth, to ensure that variability in DEHP metabolite availability did not influence our estimates, we repeated the analysis using only metabolites consistently measured across cohorts, excluding MEHP from pre- and postnatal analyses, and excluding MECPP from the postnatal analysis. Last, we derived E-values from our estimates to evaluate the potential for unmeasured confounding [57].

#### Overall mixture associations

Since 77.9% of children (*n* = 2930) did not have DMP, BPS, and BPF measured during pregnancy, these compounds were excluded from the mixture analysis. Similarly, BPA was excluded from the postnatal analysis as it was not measured in CHILD. Participants who did not have all exposures measured were excluded, resulting in 2402 children in the prenatal analyses and 1654 in the postnatal analyses. We employed Quantile G-computation to estimate the association of the chemical mixture with allergic conditions [58]. This method fits a marginal structural model with quantised exposure, allowing the estimation of the effect of increasing all exposures by one quantile simultaneously. We estimated RRs associated with a one-quartile increase in the chemical mixture, considering subjects as random intercepts, with 95% CI estimated via 1000 bootstrap replicates. Quantile G-computation also accommodates non-linear relationships between the chemical mixture and the outcome. We increased the number of quantiles to 20 to plot dose-response curves between the chemical mixtures and outcomes. We also added second- and third-order polynomials on top of restricted cubic splines and selected the models with the best Akaike Information Criterion (AIC) (Table S4). Finally, we used the *qgcompint* package (version 0.7.0) to assess the interaction between sex and the overall chemical mixture [59]. All statistical analyses were performed using R version 4.2.2 [60].

## Results

### Sample characteristics

The study population included 5306 children, with 3444 having exposure assessed during pregnancy, 1543 during childhood, and 319 during both periods (Table 2). Further details of characteristics for each of the included cohorts are provided in Table S5. The average maternal age was approximately 30 years for all cohorts. The study population was predominantly Caucasian/White, ranging from 53.9% in ECHO (*n* = 1376) to 76.7% in BIS (*n* = 633). Single parenthood was the least common in BIS (*n* = 24, 2.9%), and most common in ECHO (*n* = 619, 24.5%). BIS had the highest prevalence of a family history of asthma (*n* = 411, 49.4%), while the CHILD cohort had the lowest (*n* = 324, 21.1%). The proportion of children having asthma in the first five years ranged from 11.6% in CHILD (*n* = 132) to 21.7% in BIS (*n* = 140). The proportion of wheezing varied across cohorts, with ECHO showing the lowest rates (*n* = 712, 27.9%), and BIS showing the highest (*n* = 466, 67.2%). The proportion of children with eczema ranged from 36.5% (*n* = 790) in ECHO to 66.4% (*n* = 805) in CHILD. Finally, the prevalence of rhinitis ranged from 9.6% in CHILD (*n* = 125), to 63.1% in HOME (*n* = 238).

**Table 2:** Description of study participants.

	Prenatal analysis *N* (%) or mean ± SD				Postnatal analysis *N* (%) or mean ± SD		
	ECHO (*N* = 2553)	BIS (*N* = 833)	HOME (*N* = 377)	Overall (*N* = 3763)	CHILD (*N* = 1543)	HOME (*N* = 319)	Overall (*N* = 1862)
**Socioeconomic status**							
Marital status							
Single/Not married	619 (24.5)	24 (2.9)	77 (20.4)	720 (19.3)	83 (5.5)	59 (18.5)	142 (7.8)
Other	1908 (75.5)	806 (97.1)	300 (79.6)	3014 (80.7)	1426 (94.5)	260 (81.5)	1686 (92.2)
Missing	26	3	0	29	34	0	34
Caregiver education							
High school or under	673 (26.5)	80 (9.6)	65 (17.2)	818 (21.8)	65 (4.3)	51 (16.0)	116 (6.4)
Bachelor’s degree	734 (28.9)	267 (32.1)	111 (29.4)	1112 (29.7)	834 (55.6)	100 (31.3)	934 (51.3)
Master’s or doctorate	636 (25.0)	180 (21.6)	115 (30.5)	931 (24.8)	418 (27.8)	100 (31.3)	518 (28.5)
Other	498 (19.6)	305 (36.7)	86 (22.8)	889 (23.7)	184 (12.3)	68 (21.3)	252 (13.8)
Missing	12	1	0	13	42	0	42
Child ethnicity							
Caucasian/White	1376 (53.9)	633 (76.7)	234 (62.2)	2243 (59.7)	1094 (71.2)	210 (66.0)	1304 (70.3)
Other	1177 (46.1)	192 (23.3)	142 (37.8)	1511 (40.3)	442 (28.8)	108 (34.0)	550 (29.7)
Missing	0	8	1	9	7	1	8
**Family Characteristics**							
Maternal age (years)	29.9 ± 5.8	32.4 ± 4.5	29.7 ± 5.7	30.4 ± 5.6	32.2 ± 4.6	29.9 ± 5.5	31.8 ± 4.9
Missing	0	0	0	0	0	0	0
Family history of asthma	942 (37.7)	411 (49.4)	112 (29.7)	1465 (39.5)	324 (21.1)	99 (31.0)	423 (22.8)
Missing	52	1	0	53	10	0	10
Prenatal smoke exposure	161 (6.4)	100 (12.1) ^a^	281 (74.5) ^b^	542 (14.5)	264 (17.5)	237 (74.3) ^b^	501 (27.4)
Missing	24	6	0	30	33	0	33
**Child Characteristics**							
Child sex							
Male	1315 (51.5)	432 (51.9)	170 (45.1)	1,917 (50.9)	826 (53.5)	144 (45.1)	970 (52.1)
Female	1238 (48.5)	401 (48.1)	207 (54.9)	1846 (49.1)	717 (46.5)	175 (54.9)	892 (47.9)
Missing	0	0	0	0	0	0	0
Season of birth							
Spring	660 (25.9)	176 (21.1)	96 (25.5)	932 (24.8)	453 (29.4)	83 (26.0)	536 (28.8)
Summer	631 (24.7)	196 (23.5)	77 (20.4)	904 (24.0)	497 (32.2)	67 (21.0)	564 (30.3)
Autumn	623 (24.4)	206 (24.7)	96 (25.5)	925 (24.6)	295 (19.1)	81 (25.4)	376 (20.2)
Winter	639 (25.0)	255 (30.6)	108 (28.6)	1002 (26.6)	298 (19.3)	88 (27.6)	386 (20.7)
Missing	0	0	0	0	0	0	0
Gestational age (weeks)	38.6 ± 2.0	39.3 ± 1.2	38.9 ± 1.8	38.8 ± 1.9	39.1 ± 1.4	38.9 ± 1.8	39.1 ± 1.5
Missing	0	0	0	0	0	0	0
Breastfeeding duration (weeks)	24.7 ± 32.7	23.8 ± 17.1	23.7 ± 24.4	24.2 ± 26.8	42.7 ± 27.2	24.8 ± 24.0	39.6 ± 27.6
Missing	1,792	332	1	2,125	40	0	40
Postnatal smoke exposure	437 (19.0)	8 (1.5)	163 (49.1) ^b^	608 (19.2)	495 (32.1)	149 (49.0) ^b^	644 (34.9)
Missing	251	293	45	589	2	15	17
**Child outcomes**							
Asthma	248 (11.7)	140 (21.7)	53 (19.9)	441 (14.6)	132 (11.6)	47 (19.5)	179 (13.0)
Missing	435	189	110	734	405	78	483
Wheeze	712 (27.9)	466 (67.2)	198 (52.5)	1376 (38.0)	566 (61.1)	178 (55.8)	744 (59.8)
Missing	0	140	0	140	617	0	617
Eczema	790 (36.5)	227 (34.5)	220 (58.4)	1,237 (38.7)	805 (66.4)	192 (60.2)	997 (65.1)
Missing	389	175	0	564	331	0	331
Rhinitis	592 (35.2)	104 (17.2)	238 (63.1)	934 (35.0)	125 (9.6)	217 (68.0)	342 (21.0)
Missing	870	228	0	1098	235	0	235

Proportions exclude missing data.

^a^Includes preconception and prenatal tobacco smoke.

^b^Measured with serum cotinine.

With the exception of DEP, children had higher urinary phthalates and bisphenols concentrations relative to body weight than pregnant women (Table 3). Average exposure levels ranged from 5 ng/kg/day for BPF during pregnancy to 6.6 µg/kg/day for DEHP during childhood. As expected, we found variations in exposure levels between cohorts, with BIS showing lower levels of bisphenols and HOME exhibiting the highest levels of DEHP (Tables S6–S7).

**Table 3:** Distribution of exposure levels in the prenatal and postnatal periods.

	*N*	Cohorts	GM (GSD)	25th percentile	50th percentile	75th percentile	Max
DMP							
Prenatal	2024	4	0.05 (4.40)	0.01	0.05	0.13	6.98
Postnatal	0	0	—	—	—	—	—
DEP							
Prenatal	3553	10	1.52 (5.82)	0.53	1.52	4.65	1578.88
Postnatal	1854	2	1.23 (2.77)	0.65	1.12	2.05	210.19
DBP							
Prenatal	3553	10	0.90 (3.62)	0.49	1.11	2.06	44.70
Postnatal	1709	2	2.28 (1.96)	1.51	2.22	3.39	87.99
BBzP							
Prenatal	3553	10	0.20 (4.53)	0.08	0.22	0.53	26.73
Postnatal	1848	2	0.41 (3.00)	0.19	0.41	0.85	23.28
DEHP							
Prenatal	3553	10	3.85 (3.78)	1.55	3.55	8.92	1017.24
Postnatal	1853	2	6.62 (2.55)	3.68	6.12	11.27	161.59
MCPP							
Prenatal	3454	9	1.40 (3.92)	0.70	1.51	3.18	1024.92
Postnatal	1853	2	1.31 (2.72)	0.75	1.28	2.29	35.07
BPA							
Prenatal	2796	9	0.02 (5.07)	0.01	0.02	0.05	1.98
Postnatal	319	1	0.17 (2.13)	0.11	0.16	0.27	7.60
BPS							
Prenatal	2321	7	0.005 (6.65)	0.002	0.006	0.016	3.091
Postnatal	0	0	—	—	—	—	—
BPF							
Prenatal	2081	6	0.003 (15.97)	0.001	0.007	0.018	4.994
Postnatal	0	0	—	—	—	—	—

Estimated daily intake (µg/kg/day), except MCPP: metabolite concentration (µg/L).

*BBzP* Benzyl butyl phthalate, *BPA* Bisphenol A, *BPF* Bisphenol F, *BPS* Bisphenol S, *DBP* Dibutyl phthalate, *DEHP* Di(2-ethylhexyl) phthalate, *DEP* Diethyl phthalate, *DMP* Dimethyl phthalate, *GM* Geometric mean, *GSD* Geometric standard deviation, *MCPP* Mono(3-carboxypropyl) phthalate.

### Single-compound associations

#### Prenatal analysis

**Fig. 1:**
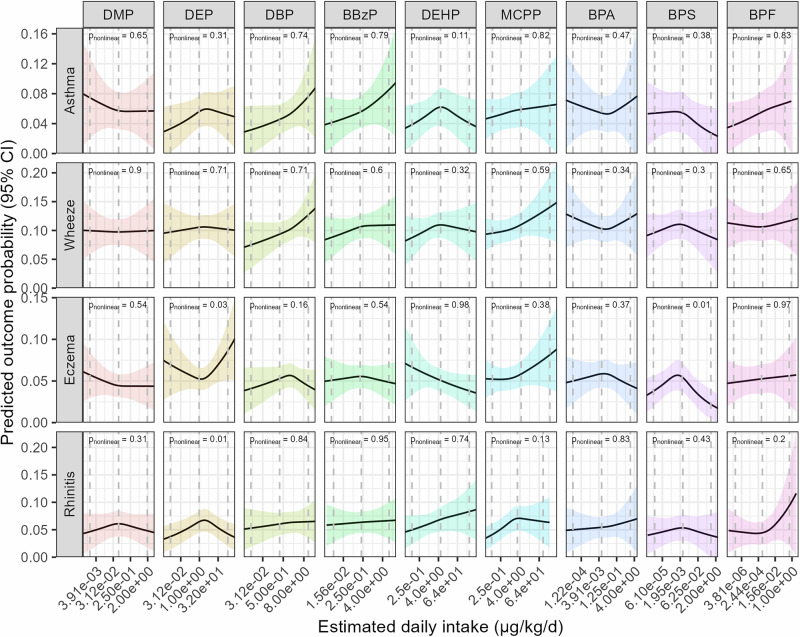
Univariate dose-response relationship between prenatal phthalates and bisphenols and childhood allergic conditions. Models obtained with generalised estimating equations. Dose-response relationships are displayed with continuous covariates held at their mean values and categorical covariates set to their reference category. *P*-values for non-linearity obtained with Wald tests on spline terms. Models adjusted for cohort membership, maternal age, ethnicity, parental education, marital status, family history of asthma, sex, prenatal tobacco smoke exposure, and season of birth. All exposures are modelled as estimated daily intakes, except MCPP, which is modelled using biomarker concentrations.

We found that each two-fold increase in prenatal DBP levels was associated with a higher risk of asthma (RR = 1.08; 95% CI: 1.00–1.16) and wheezing (RR = 1.04; 95% CI: 1.00–1.08) (Table 4). Similarly, a two-fold increase in prenatal BBzP levels was associated with a higher risk of asthma (RR = 1.06; 95% CI: 1.00–1.12). Additionally, MCPP levels were positively associated with the risk of wheezing (RR = 1.03; 95% CI: 1.00–1.05) and allergic rhinitis (RR = 1.05; 95% CI: 1.01–1.09), while DEHP showed an inverse relationship with eczema (RR = 0.97; 95% CI: 0.93–1.00). No other associations were observed between prenatal urinary phthalates, bisphenols, and health outcomes. Crude estimates are presented in Table S8. While most associations were linear, a few demonstrated non-linearity (Fig. 1). For instance, there were inverted U-shaped associations between prenatal urinary BPS and eczema, and between DEP and rhinitis. Furthermore, the association between DEP and eczema exhibited a U-shaped relationship.

**Table 4:** Adjusted risk ratios of allergic conditions associated with a twofold increase in prenatal phthalates and bisphenols.

	Asthma			Wheeze		
	Cohorts	*N*	RR (95%CI)	Cohorts	*N*	RR (95%CI)
DMP	4	1927	0.97 (0.92–1.04)	4	1983	1.00 (0.96–1.04)
DEP	8	2900	1.02 (0.98–1.07)	10	3447	1.00 (0.98–1.03)
DBP	8	2900	1.08 (1.00–1.16)	10	3447	1.04 (1.00–1.08)
BBzP	8	2900	1.06 (1.00–1.12)	10	3447	1.01 (0.99–1.04)
DEHP	8	2900	1.00 (0.94–1.07)	10	3447	1.01 (0.97–1.04)
MCPP	7	2812	1.02 (0.97–1.08)	9	3344	1.03 (1.00–1.05)
BPA	7	2163	0.99 (0.95–1.05)	9	2710	1.00 (0.97–1.02)
BPS	5	1808	0.98 (0.93–1.03)	7	2231	1.00 (0.97–1.03)
BPF	4	1570	1.03 (0.99–1.07)	6	1993	1.00 (0.98–1.02)

	Eczema			Rhinitis		
	Cohorts	*N*	RR (95%CI)	Cohorts	*N*	RR (95%CI)
DMP	4	1947	0.98 (0.95–1.02)	4	1869	1.01 (0.96–1.05)
DEP	9	3111	1.02 (0.99–1.04)	8	2562	1.00 (0.98–1.03)
DBP	9	3111	1.01 (0.97–1.04)	8	2562	1.01 (0.97–1.05)
BBzP	9	3111	1.00 (0.97–1.03)	8	2562	1.01 (0.97–1.04)
DEHP	9	3111	0.97 (0.93–1.00)	8	2562	1.03 (0.99–1.07)
MCPP	8	3023	1.03 (0.99–1.06)	7	2474	1.05 (1.01–1.09)
BPA	8	2409	0.99 (0.96–1.02)	7	1860	1.01 (0.97–1.05)
BPS	6	1945	0.98 (0.94–1.01)	5	1396	1.01 (0.96–1.05)
BPF	5	1707	1.01 (0.98–1.04)	4	1158	1.03 (0.99–1.07)

Models obtained with generalised estimating equations. Models adjusted for cohort membership, maternal age, ethnicity, parental education, marital status, family history of asthma, sex, prenatal tobacco smoke exposure, and season of birth. All exposures are modelled as estimated daily intakes, except MCPP, which is modelled using biomarker concentrations.

*BBzP* Benzyl butyl phthalate, *BPA* Bisphenol A, *BPF* Bisphenol F, *BPS* Bisphenol S, *DBP* Dibutyl phthalate, *DEHP* Di(2-ethylhexyl) phthalate, *DEP* Diethyl phthalate, *DMP* Dimethyl phthalate, *MCPP* Mono(3-carboxypropyl) phthalate, *RR* Risk Ratio.

#### Postnatal analysis

**Fig. 2:**
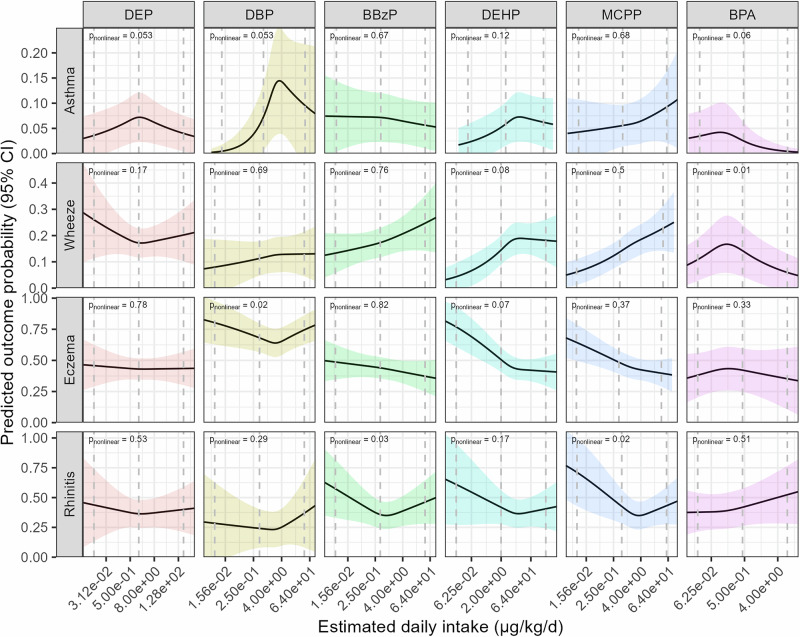
Univariate dose‒response relationship between postnatal phthalates and bisphenols and childhood allergic conditions. Models obtained with generalised estimating equations. Dose-response relationships are displayed with continuous covariates held at their mean values and categorical covariates set to their reference category. *P*-values for non-linearity obtained with Wald tests on spline terms. Models adjusted for cohort membership, maternal age, ethnicity, parental education, marital status, family history of asthma, sex, prenatal tobacco smoke exposure, season of birth, breastfeeding duration, age at outcome assessment, postnatal smoke exposure and gestational age. All exposures are modelled as estimated daily intakes, except MCPP, which is modelled using biomarker concentrations.

We found some evidence that high-molecular-weight phthalates, such as BBzP (RR = 1.05; 95% CI: 1.01–1.09), DEHP (RR = 1.06; 95% CI: 1.01–1.11), and MCPP (RR = 1.09; 95% CI: 1.04–1.14), were associated with a higher risk of wheezing (Table 5). Conversely, high-molecular-weight phthalates were inversely associated with eczema, such as BBzP (RR = 0.98; 95% CI: 0.95–1.00), DEHP (RR = 0.95; 95% CI: 0.91–0.98), and MCPP (RR = 0.95; 95% CI: 0.92–0.98). Crude estimates are shown in Table S9. We found a U-shape dose-response relationship between DBP and eczema, and between MCPP and rhinitis (Fig. 2). Furthermore, the association between BPA and wheeze followed an inversed-U relationship.

**Table 5:** Adjusted risk ratios of allergic conditions associated with a twofold increase in postnatal phthalates and bisphenols.

	Asthma			Wheeze		
	Cohorts	*N*	RR (95%CI)	Cohorts	*N*	RR (95%CI)
DEP	2	1175	0.98 (0.93–1.03)	2	1719	0.99 (0.95–1.03)
DBP	2	1098	1.11 (0.99–1.25)	2	1589	1.03 (0.96–1.09)
BBzP	2	1184	0.98 (0.93–1.03)	2	1720	1.05 (1.01–1.09)
DEHP	2	1179	1.01 (0.95–1.09)	2	1718	1.06 (1.01–1.11)
MCPP	2	1184	1.06 (0.98–1.16)	2	1719	1.09 (1.04–1.14)
BPA	1	235	0.89 (0.78–1.01)	1	303	0.98 (0.90–1.06)

	Eczema			Rhinitis		
	Cohorts	*N*	RR (95%CI)	Cohorts	*N*	RR (95%CI)
DEP	2	1707	1.00 (0.97–1.02)	2	1273	1.00 (0.96–1.04)
DBP	2	1576	1.01 (0.97–1.06)	2	1149	1.04 (0.94–1.16)
BBzP	2	1707	0.98 (0.95–1.00)	2	1280	0.99 (0.95–1.04)
DEHP	2	1705	0.95 (0.91–0.98)	2	1276	0.99 (0.94–1.05)
MCPP	2	1707	0.95 (0.92–0.98)	2	1279	0.98 (0.93–1.04)
BPA	1	303	1.00 (0.95–1.06)	1	303	1.03 (0.98–1.09)

Models obtained with generalised estimating equations. Models adjusted for cohort membership, maternal age, ethnicity, parental education, marital status, family history of asthma, sex, prenatal tobacco smoke exposure, season of birth, breastfeeding duration, age at outcome assessment, postnatal smoke exposure and gestational age. All exposures are modelled as estimated daily intakes, except MCPP, which is modelled using biomarker concentrations.

*BBzP* Benzyl butyl phthalate, *BPA* Bisphenol A, *DBP* Dibutyl phthalate, *DEHP* Di(2-ethylhexyl) phthalate, *DEP* Diethyl phthalate, *DMP* Dimethyl phthalate, *MCPP* Mono(3-carboxypropyl) phthalate, *RR* Risk Ratios.

### Overall mixture associations

**Fig. 3:**
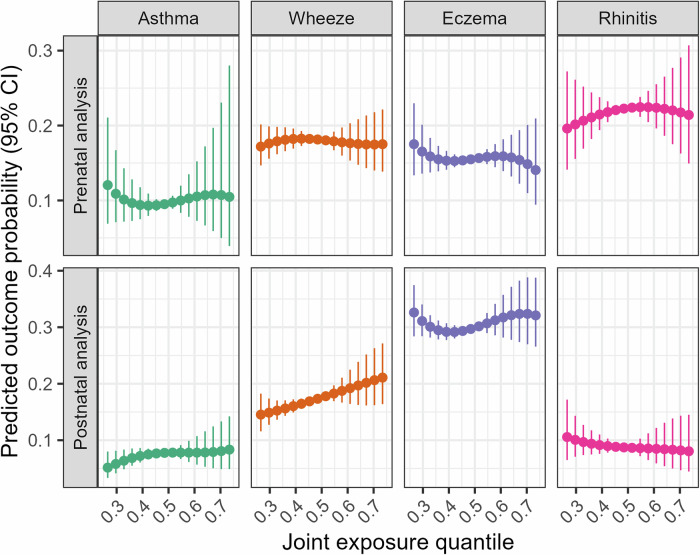
Multivariate dose‒response relationship between the overall chemical mixture and childhood allergic conditions, using quantile G computation. Dose-response relationships are displayed with continuous covariates held at their mean values and categorical covariates set to their reference category. Prenatal analysis models adjusted for cohort membership, maternal age, ethnicity, parental education, marital status, family history of asthma, sex, prenatal tobacco smoke exposure, and season of birth. Postnatal analysis models were further adjusted for breastfeeding duration, age at outcome assessment, postnatal smoke exposure and gestational age.

Using quantile G computation, there was no association between prenatal phthalates and bisphenols mixture and outcomes (Table 6). However, the postnatal chemical mixture was positively associated with wheezing (RR = 1.14; 95%CI: 1.02–1.26), with MCPP showing the highest weight (Fig. S7). Except for a linear association between the postnatal chemical mixture and wheeze, there was no clear pattern of association for other outcomes (Fig. 3).

**Table 6:** Risk Ratios of allergic conditions associated with a quartile increase in phthalates and bisphenols mixture.

	*N*	Risk Ratio (95%CI)	
		Crude	Adjusted^a^
Prenatal analysis			
Asthma	1871	1.25 (1.02–1.54)	1.10 (0.83–1.46)
Wheeze	2402	1.07 (0.98–1.18)	1.04 (0.93–1.16)
Eczema	2116	1.33 (1.18–1.50)	0.95 (0.85–1.06)
Rhinitis	1567	1.34 (1.20–1.50)	1.03 (0.89–1.19)
Postnatal analysis			
Asthma	1083	1.50 (1.19–1.90)	1.17 (0.91–1.50)
Wheeze	1586	1.12 (1.01–1.24)	1.14 (1.02–1.26)
Eczema	1574	0.93 (0.87–1.01)	0.98 (0.91–1.05)
Rhinitis	1137	1.47 (1.18–1.83)	0.89 (0.68–1.17)

Models obtained using quantile G-computation.

^a^Prenatal analysis models adjusted for cohort membership, maternal age, ethnicity, parental education, marital status, family history of asthma, sex, prenatal tobacco smoke exposure, and season of birth. Postnatal analysis models were further adjusted for breastfeeding duration, age at outcome assessment, postnatal smoke exposure and gestational age.

### Effect measure modification by sex

**Fig. 4:**
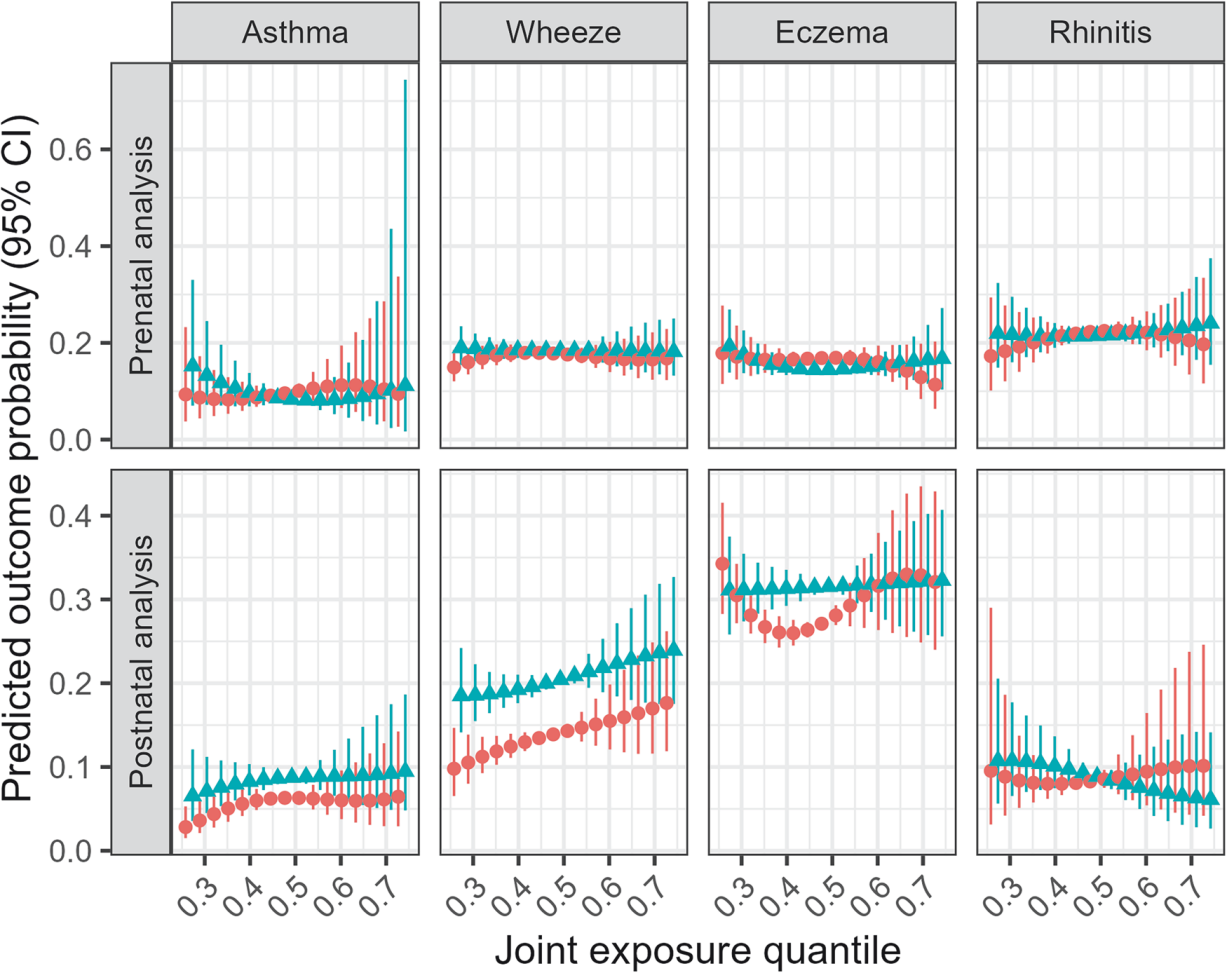
Multivariate dose‒response relationship between the overall chemical mixture and childhood allergic conditions stratified by sex. Red circles: Females. Blue triangles: Male. Models obtained with quantile G-computation. Prenatal analysis models adjusted for cohort membership, maternal age, ethnicity, parental education, marital status, family history of asthma, prenatal tobacco smoke exposure, and season of birth. Postnatal analysis models were further adjusted for breastfeeding duration, age at outcome assessment, postnatal smoke exposure and gestational age.

Although evidence of effect measure modification by sex was limited in single compound models, postnatal DEHP was associated with a higher risk of wheezing in females (RR = 1.11; 95%CI: 1.04–1.18; *p* = 0.04 for interaction; Tables S10–S11), and a lower risk of rhinitis in males (RR = 0.94; 95% CI: 0.87–1.00, *p* = 0.02 for interaction). Additionally, the association between pre- and postnatal DEP levels and asthma followed an inverted U-shape pattern in females, but not in males (p_prenatal_ < 0.001 for interaction; p_postnatal_ = 0.04 for interaction; Fig. S8–S9). No evidence of effect measure modification by sex was found when using quantile G-computation (Table 7). However, the relationship between the postnatal chemical mixtures and eczema exhibited a U-shaped pattern only in females (Fig. 4).

**Table 7:** Sex-specific effect measure modification in the association between the overall chemical mixture and childhood allergic conditions, stratified by sex.

	Adjusted Risk Ratio (95% CI) ^a^		*p*-value ^b^
	Male	Female	
Prenatal analysis			
Asthma	1.07 (0.86–1.33)	1.18 (0.94–1.47)	0.72
Wheeze	1.00 (0.90–1.12)	1.11 (0.98–1.25)	0.37
Eczema	0.97 (0.85–1.10)	0.89 (0.78–1.01)	0.49
Rhinitis	1.04 (0.88–1.22)	1.02 (0.87–1.20)	0.91
Postnatal analysis			
Asthma	1.18 (0.84–1.65)	1.29 (0.84–1.97)	0.74
Wheeze	1.11 (0.95–1.30)	1.29 (1.06–1.58)	0.19
Eczema	1.00 (0.86–1.16)	0.94 (0.80–1.10)	0.55
Rhinitis	0.71 (0.50–1.01)	1.01 (0.69–1.49)	0.33

Models obtained using quantile G-computation.

^a^Prenatal analysis models adjusted for cohort membership, maternal age, ethnicity, parental education, marital status, family history of asthma, sex, prenatal tobacco smoke exposure, and season of birth. Postnatal analysis models were further adjusted for breastfeeding duration, age at outcome assessment, postnatal smoke exposure and gestational age.

^b^*p*-value for sex interaction.

### Susceptible window of exposure during pregnancy

We found that the association of prenatal exposure to phthalates and bisphenols with childhood allergic conditions differed by trimester of exposure (Fig. S10–S13). There were stronger associations for first-trimester BPS (RR = 2.45; 95% CI: 1.42, 4.23) and BBzP (RR = 1.43; 95% CI: 1.20–1.71), with asthma, but also with first-trimester MCPP and eczema (RR = 1.12; 95% CI: 1.00–1.24). Second-trimester DBP levels (RR = 1.14; 95% CI: 1.03–1.26), BBzP (RR = 1.11; 95% CI: 1.02–1.21), and MCPP (RR = 1.13; 95% CI: 1.04–1.24) were associated with a higher risk of asthma. Furthermore, second-trimester BPF was positively associated with eczema (RR = 1.08; 95% CI: 1.01–1.15), while MCPP was associated with rhinitis (RR = 1.06; 95% CI: 1.01–1.12) and wheezing (RR = 1.05; 95% CI: 1.00–1.10). Regarding third-trimester exposures, only DEP levels were associated with eczema (RR = 1.05; 95% CI: 1.01–1.09).

### Sensitivity analyses

Repeating the analysis using urinary biomarkers rather than estimated daily intakes did not substantially change our estimates (Table S12–S13). The heterogeneity between cohorts, measured by the I^2^ statistic, was generally low, below 40% in the prenatal analysis, indicating a reasonable degree of replication across cohorts (Fig. S14–S17). However, the postnatal analysis had higher heterogeneity, except for eczema (Fig. S18–S21). Point estimates remained generally unchanged when combining cohort-specific effect sizes in a random-effect meta-analysis. However, confidence intervals were wider in some cases due to moderate to high heterogeneity (Fig. S22–S23). The imputation of missing values or using IPW to account for potential selection bias did not substantially change our findings (Fig. S22–S23). Furthermore, restricting DEHP metabolites to those measured in all cohorts did not substantially change our estimates (Table S14). Last, an unmeasured confounder would need to be associated with both prenatal DBP exposure and childhood asthma by a risk ratio of at least 1.37 to fully explain the observed association. For the postnatal phthalate mixture and wheeze, the corresponding E-value was 1.54.

## Discussion

This pooled analysis suggests that prenatal and postnatal levels of specific phthalates and bisphenols are marginally associated with asthma, wheezing and allergic rhinitis in children up to five. Additionally, phthalates and bisphenols chemical mixtures during childhood were associated with childhood wheezing. However, we found inverse associations between phthalates and eczema. In some cases, the associations were non-linear, exhibiting either a U-shape or an inverse U-shape dose-response relationship. Finally, the risk of childhood allergic conditions associated with prenatal phthalates and bisphenols might differ by trimester of exposure.

Many studies have investigated whether phthalates and bisphenols are associated with asthma and wheezing during childhood, yet results have been inconsistent. Our findings on prenatal DBP and BBzP with asthma align with those of the *Infancia y Medio Ambiente* (INMA) study [6] and the Columbia Centre for Children’s Environmental Health Cohort [61]. However, results from Berger et al. suggested no association between MiBP and MnBP during the second trimester of pregnancy and asthma at seven years of age [10]. While differences in exposure windows may explain different findings, the Raine study reported reduced odds of persistent asthma and higher odds for transient asthma associated with serum MBzP during the second and third trimesters of pregnancy [62], suggesting these chemicals may affect different asthma phenotypes. Overall, there was no strong evidence of effect measure modification by sex, as the magnitude of estimates remained similar across sexes, suggesting that the observed differences may be due to chance rather than true modification. While some studies have suggested effect measure modification by sex [10, 62], others have reported limited or no sex-specific effects [6, 63–65], indicating that the evidence for sex-specific effects of phthalates and bisphenols on childhood allergic outcomes remains limited.

Surprisingly, we found inverse associations between high-molecular-weight phthalates and eczema. Few studies reported similar findings between prenatal phthalates and childhood eczema [8, 63, 64, 66, 67]. Moreover, many studies investigating childhood exposure and eczema lacked a clear temporal relationship between exposure and outcome. Children with eczema may use more personal care products, such as creams and ointments, potentially leading to higher phthalate and bisphenol exposure and inflated estimates [68]. Only a few cohort studies have demonstrated temporal precedence between childhood measurements and outcomes. The Polish Mother and Child cohort reported an inverse association between longitudinal MEOHP measurements and atopic dermatitis at age nine [8]. In contrast, two other cohort studies found no association between phthalate exposure during early childhood and subsequent eczema [69, 70]. In our postnatal analysis, we ensured that exposure measurements preceded the outcome, mitigating reverse causation. Since most reported associations between childhood phthalate exposure and eczema are cross-sectional, further longitudinal studies maintaining exposure-outcome timing are necessary to confirm these findings.

Although our estimates were modest and unlikely to be clinically important, plastic chemical exposure is chronic and widespread [2, 3]. Thus, population-level strategies to reduce exposure could decrease the burden of asthma and wheezing in developed countries over time [71]. In our mixture analysis, dibutyl and HMW phthalates had the highest relative weights for asthma and wheezing. While these chemicals are primarily introduced through diet, a randomised study found that dietary interventions failed to reduce phthalate levels [72], suggesting that further studies are warranted to develop effective strategies for reducing plastic chemical exposure at a population level.

Human evidence on the susceptibility window of EDC exposure is limited. Consistent with our results, the INMA cohort study reported that first-trimester phthalate exposure were associated with a higher risk of asthma, wheezing, and eczema in children aged four to seven, whereas third-trimester exposure did not [6]. Since airway development is completed by 16–18 gestational weeks, this period may represent a critical window of susceptibility [73]. However, these results should be interpreted cautiously, as the estimates for different trimesters were based on different subsets of the study population, with small sample sizes for the first trimester.

Phthalates and bisphenols may induce childhood allergic conditions through multiple pathways, although mechanisms are not fully understood [4]. Experimental studies support the role of DBP and BBzP in the development of asthma. In mice, oral DBP administration increased Th2 and Th17 cytokine infiltration in lung tissue and elevated oxidative stress biomarkers [74], supporting its role in airway inflammation. Furthermore, gestational BBzP exposure in mice led to offspring airway inflammation via global DNA hypermethylation in CD4 cells [75], aligning with our findings. Phthalates with 8 carbon atoms on their side chains, such as DEHP, had the strongest immunostimulatory effects [76]. This aligns with our findings of postnatal exposure to these phthalates being associated with wheezing, suggesting that structural differences between phthalates may influence their biological activity. Regarding eczema, while most murine studies suggest that phthalates exacerbate eczema, these studies often used non-environmentally relevant doses and mainly focused on their adjuvant effects rather than direct eczema onset [4]. Interestingly, one study reported that subcutaneous injection of high-molecular-weight phthalates in ovalbumin-sensitised mice reduced IgE production [77], potentially supporting the inverse association we observed. Nonetheless, further studies are warranted to determine the relevance of these mechanisms in humans.

Strengths of this study include the integration of four different data sources from various populations combined with a uniform analytical strategy. Thus, decreasing the heterogeneity of our estimates and enhancing the generalisability of our results within high-income, English-speaking populations. By increasing sample size and exposure range, we captured relationships and dose responses that may have gone undetected in smaller cohorts. To our knowledge, this is the largest study investigating urinary phthalate and bisphenol exposure and childhood allergic conditions across both the prenatal and postnatal periods. We also estimated the overall effects of chemical mixtures through longitudinal associations. Finally, most cohorts had repeated urinary samples available, offering more reliable exposure assessments than single-spot urine samples [78].

Limitations include the large variations in outcome prevalence rates, mainly due to differences in assessment methods and age at evaluation. Similarly, exposure assessment methods varied across cohorts, further contributing to heterogeneity and potentially affecting the precision of our findings. However, the literature on this topic is often conflicting, and pooling multiple cohorts with diverse exposure and outcome assessment methods allowed us to evaluate the robustness of previously reported associations rather than relying on a single study. While clinical assessment is the gold standard for diagnosing outcomes, caregiver-reported outcomes might introduce potential misclassification. Nevertheless, validated surveys, such as the ISAAC questionnaire, have demonstrated 87% sensitivity and 100% specificity for asthma in children aged 6–7 years [79]. Similarly, the UK Working Party criteria for eczema, used in the BIS and CHILD cohorts, have shown 85% sensitivity and 96% specificity for childhood eczema [41]. Detection rates for bisphenols were lower than for phthalates, likely resulting in reduced exposure levels compared to other studies of bisphenols. This may have diminished exposure variability and limited our capacity to identify associations. Additionally, models for bisphenols also had smaller sample sizes, which may lack adequate statistical power to detect small effect sizes. Furthermore, our mixture approach did not specifically examine co-adjusted compounds or co-exposure multiplicative interactions. We cannot rule out the potential for residual confounding from maternal diet, household factors, or other correlated environmental chemicals that may have influenced our estimates and dose-responses. Furthermore, the aggregation of covariate categories, though necessary, may have led to some residual confounding. While the direction and extent of total unmeasured confounding remain uncertain, an unmeasured confounder would need a 1.37-fold association with both exposures and outcomes to nullify the association between prenatal DBP exposure and asthma [57].

In summary, this pooled analysis adds to the growing body of evidence that EDCs are associated with respiratory and allergic outcomes, with novel observations in children under five. Our findings on asthma align with previous research, reinforcing the potential risk that specific phthalates pose during critical developmental windows. Furthermore, the observed inverse relationship with eczema requires further investigation. Future studies should focus on longitudinal designs with repeated exposure measurements, ensure proper exposure-outcome timing precedence, and explore potential mechanisms.

## Supplementary information

Supplementary Materials

## Data Availability

The datasets generated during and/or analysed during the current study are not publicly available due privacy and ethical reasons but are available from the corresponding author on reasonable request.
