# Supplementary material for: Phthalates and bisphenols early-life exposure, and childhood allergic conditions: a pooled analysis of cohort studies
Source: J Expo Sci Environ Epidemiol. 2025 Jul 3;35(6):965–80. doi: 10.1038/s41370-025-00790-2 (PMC12583191; doi:10.1038/s41370-025-00790-2)
Supplement: Supplementary file 1 — Supplementary Materials [file 41370_2025_790_MOESM1_ESM.docx]

**Supplementary Materials**

**Phthalates and Bisphenols Early-Life Exposure, and Childhood Allergic Conditions: A Pooled Analysis of Cohort Studies**

Thomas Boissiere-O'Neill ^1^, Nina Lazarevic ^2^, Peter D Sly ^1^, Anne-Louise Ponsonby ^3^, Aimin Chen ^4^, Meghan B. Azad ^5^, Joseph M. Braun ^6^, Jeffrey R. Brook ^7^, David Burgner ^8^, Bruce P. Lanphear ^9^, Theo J. Moraes ^10^, Richard Saffery ^8^, Padmaja Subbarao ^10^, Stuart E. Turvey ^11^, Kimberly Yolton ^12^, CHILD investigator group, BIS investigator group, Dwan Vilcins ^1^

^1^ The University of Queensland, Child Health Research Centre, The Children’s Health and Environment Program QLD, 4101, Australia.

^2^ National Centre for Epidemiology and Population Health, Australian National University, Canberra, ACT 2601, Australia

^3^ Florey Institute of Neuroscience and Mental Health, University of Melbourne, Melbourne, VIC 3010, Australia

^4^ Department of Biostatistics, Epidemiology and Informatics, Perelman School of Medicine, University of Pennsylvania, Philadelphia, PA 19104, United States

^5^ Children’s Hospital Research Institute of Manitoba, Department of Pediatrics and Child Health, University of Manitoba, Winnipeg, Canada

^6^ Department of Epidemiology, Brown University, Providence, RI, 02912

^7^ Dalla Lana School of Public Health, University of Toronto, Toronto, ON, Canada

^8^ Murdoch Children's Research Institute, Royal Children's Hospital, The University of Melbourne, Melbourne, Victoria, Australia

^9^ Faculty of Health Sciences, Simon Fraser University, Burnaby, BC, V5A 1S6, Canada

^10^ Department of Pediatrics, Hospital for Sick Children, University of Toronto, Toronto, ON, Canada

^11^ Department of Pediatrics, Child and Family Research Institute, BC Children’s Hospital, University of British Columbia, Vancouver, BC, Canada

^12^ Cincinnati Children’s Hospital Medical Center and the University of Cincinnati College of Medicine, Cincinnati, OH, USA.

#

# Table S1. Limit of detection (µg/L) of plasticiser metabolites across cohorts

|  | BIS | CHILD | HOME | AAA01 | AAG01 | AAM01 | AAV01 | AAZ01 | AAZ02 | ABA03 | AFA01 | AGA01 |
| --- | --- | --- | --- | --- | --- | --- | --- | --- | --- | --- | --- | --- |
| MMP | 0.05 | 0.15 | - | - | 0.1 | 0.03 | 0.5 | 0.14 | 0.14 | - | - | 0.1 |
| MEP | 0.34 | 0.40 | 0.5 | - | 0.4 | 0.4 | 0.6 | 0.08 | 0.08 | 1.2 | 1.2 | 1.2 |
| MiBP | 3.9 | 0.26 | 0.3 | - | 0.2 | 0.02 | 0.2 | 0.06 | 0.05 | 0.08 | 0.8 | 0.8 |
| MnBP | 4.5 | 0.40 | 0.4 | - | 0.2 | 0.04 | 0.4 | 0.04 | 0.04 | 0.4 | 0.4 | 0.4 |
| MBzP | 0.17 | 0.11 | 0.2 | - | 0.2 | 0.03 | 0.3 | 0.09 | 0.07 | 0.3 | 0.3 | 0.3 |
| MEHP | - | 0.90 | 1.2 | - | 0.2 | 0.3 | 0.5 | 0.30 | 0.08 | 0.8 | 0.8 | 0.8 |
| MEHHP | 0.13 | 0.32 | 0.7 | - | 0.4 | 0.04 | 0.2 | 0.02 | 0.03 | 0.4 | 0.4 | 0.4 |
| MEOHP | 0.10 | 0.45 | 0.7 | - | 0.4 | 0.02 | 0.2 | 0.05 | 0.03 | 0.2 | 0.2 | 0.2 |
| MECPP | 0.03 | - | 0.6 | - | 0.2 | 0.05 | 0.2 | 0.02 | 0.04 | 0.4 | 0.4 | 0.4 |
| MCPP | 0.04 | 0.16 | 0.2 | - | 0.2 | 0.22 | 0.2 | 0.09 | 0.12 | 0.4 | 0.4 | 0.4 |
| BPA | 0.24 | - | 0.4 | 0.2 | 0.4 | 0.15 | 0.1 | - | - | 0.35 | 0.2 | 0.2 |
| BPS | 0.04 | - |  | 0.1 | 0.05 | 0.08 | 0.1 | - | - | 0.02 | 0.1 | 0.1 |
| BPF | 0.25 | - |  | 0.2 | 0.50 | 0.36 | - | - | - | 0.02 | 0.2 | 0.2 |

*Abbreviations: BIS: Barwon Infant Study;BPA: Bisphenol A; BPF: Bisphenol F; BPS: Bisphenol S; CHILD: Canadian Healthy Infant Longitudinal Development; ECHO: Environmental Influences on Child Health Outcomes; HOME: Health Outcomes and Measures of the Environment MiBP: Mono-iso-butyl phthalate; MnBP: Mono-n-butyl phthalate; MBzP: Monobenzyl phthalate; MCPP: Mono(3-carboxypropyl) phthalate; MECPP: Mono(2-ethyl-5-carboxypentyl) phthalate; MEHHP: Mono(2-ethyl-5-hydroxyhexyl) phthalate; MEHP: Mono(2-ethylhexyl) phthalate; MEOHP: Mono(2-ethyl-5-oxohexyl) phthalate; MEP: Monoethyl phthalate; MMP: Monomethyl phthalate*

# Table S2. Outcome assessment across cohorts

| Cohort | Outcome included | Assessment method | Number of assessments |
| --- | --- | --- | --- |
| BIS | Asthma, Wheeze, Eczema, Rhinitis | Caregiver-reported | - Asthma: 2 (2, 4 years) - Wheeze: 8 (1 month-4 years) - Eczema: 8 (1 month-4 years) - Rhinitis: 2 (2, 4 years) |
| HOME | Asthma, Wheeze, Eczema, Rhinitis | Caregiver-reported | - Asthma: 4 (2-5 years) - Wheeze: 10 (6 months-5 years) - Eczema: 10 (6 months-5 years) - Rhinitis: 5 (1-5 years) |
| AAA01^a^ | Asthma, Wheeze, Eczema, Rhinitis | Caregiver-reported | 2 (3-5 years) |
| AAG01^a^ | Asthma, Wheeze, Eczema, Rhinitis | Caregiver-reported | 3 (2-5 years) |
| AAM01^a^ | Asthma, Wheeze, Eczema | Caregiver-reported | 2 (3-5 years) |
| AAV01^a^ | Asthma, Wheeze, Eczema, Rhinitis | Caregiver-reported | 4 (3 months – 5 years) |
| AAZ01^a^ | Asthma, Wheeze, Eczema, Rhinitis | Caregiver-reported | 1 (4, 5 years) |
| AAZ02^a^ | Asthma, Wheeze, Eczema, Rhinitis | Caregiver-reported | 1 (4-5 years) |
| ABA03^a^ | Wheeze | Caregiver-reported | 1 (6 months) |
| AFA01^a^ | Wheeze, Eczema, Rhinitis | Caregiver-reported | 2 (3 – 4 years) |
| AGA01^a^ | Asthma, Wheeze, Eczema, Rhinitis | Caregiver-reported | 7 (1 month – 5 years) |
| CHILD | Asthma, Wheeze, Eczema, Rhinitis | - Asthma, eczema, rhinitis: Clinical assessment - Wheeze: caregiver-reported | - Asthma: 2 (3, 5 years) - Wheeze: 8 (3 months, 5 years) - Eczema: 2 (3, 5 years) - Rhinitis: 2 (3, 5 years) |

*^a^: ECHO-wide cohorts*

*Abbreviations: BIS: Barwon Infant Study; CHILD: Canadian Healthy Infant Longitudinal Development; ECHO: Environmental Influences on Child Health Outcomes; HOME: Health Outcomes and Measures of the Environment*

# Table S3. Harmonisation of categorical variables

| **Variable** | **BIS** | **ECHO** | **HOME** | **CHILD** | **Variable used in the pooled analysis** |
| --- | --- | --- | --- | --- | --- |
| **Ethnicity (self-reported)** | Four the four grandparents:   - British/Irish - European - Asian - Aboriginal or Torres Strait Islander - Don’t Know - Other   Caucasian/White defined if all 4 grandparents were of British/Irish or European descent | For the child:   - White - Black or African American - American Indian or Alaska Native - Asian Indian - Other Asian - Native Hawaiian or other Pacific Islander - Other - Don’t know - Prefer not to answer | For the child:   - White - Black African American - Asian or Pacific Islander - American Indian, Eskimo, Aleut - Other - Refuse - Don’t Know | For both caregivers:   - First nations - Southeast Asian - East Asian - South Asian - Black - Middle eastern - Hispanic - Caucasian white - Other - Unknown - Mixed   Child ethnicity was defined as the parents’ ethnicity if both parents had the same, otherwise defined as mixed | For the child:   - Caucasian/white - Other |
| **Caregiver education (self-reported)** | For both caregivers:   - Less than year 10 - Year 10 or equivalent - Year 12 or equivalent - Trade certificate/apprenticeship - Bachelor’s degree - Postgraduate degree - Other - Don't know | For both caregivers:   - No schooling / never attended - 8th grade or less - Some high school, no degree - High school degree - GED or equivalent - Some college, no degree - Associate’s degree - Bachelor’s degree - Master’s degree - Professorial or Doctorate Degree - Prefer not to answer - Don’t know | For both caregivers:   - No formal schooling - 8th grade or less - Some high School - High school diploma - GED - Some college or 2-year degree - Technical or trade school - Bachelor’s degree - Graduate or professional school - Refuse - Don't know | For both caregivers:   - Less than high school - Some high school - Completed high school - Some college - Completed college - Some university - Completed university - Master’s degree - PhD | For both caregivers:   - High school or under - Bachelor’s degree - Diploma or certificate - Master’s or Doctorate   Caregiver education defined as the maximal education level between both parents |
| **Marital status (self-reported)** | For the primary caregiver:   - Single - Married - Not married but living together with a partner of the opposite sex - Not married but living together with a partner of the same sex - Widowed - Divorced - Separated - Declined to answer - Don't know | For the primary caregiver:   - Married to a partner of the opposite sex - Married to a partner of the same sex - Not married but living with a partner of the opposite sex - Not married but living with a partner of the same sex - Widowed - Separated - Divorced - Single, never married - Prefer not to answer - Don’t know | For the primary caregiver:   - Married or common law - Single (never been married) - Divorced or separated | For the primary caregiver:   - Married, living together - Married, living apart - Not married, but living with someone - Not married, living alone - Not applicable - Refuse - Don't know | For the primary caregiver:   - Single/Not married - Other |
| **Family history of asthma**  **(self-reported)** | Defined if one of the caregivers or child’s siblings ever been told by a doctor that they had asthma (binary) | Defined if one of the caregivers or child’s siblings ever been told by a doctor that they had asthma (binary) | Defined if one of the caregivers ever been told by a doctor that they had asthma (binary) | Defined if one of the caregivers or child’s siblings ever been told by a doctor that they had asthma (binary) | Binary (yes/no) |
| **Prenatal smoke exposure** | Any periconceptional (3 months before conception) or gestational active or passive tobacco smoke exposure (self-reported) | Any active tobacco or nicotine products use during pregnancy OR smoking in the mother's home(s) or dwelling(s) during the child's pregnancy? (self-reported) | Cotinine measured in the mother’s serum at 16 and 26 gestational weeks above limit of detection (0.015 ng/ml) | Any gestational active or passive tobacco smoke exposure (self-reported) | Binary (yes/no) |
| **Postnatal smoke exposure** | Any smoking inside the house during the first four years of life (self-reported) | Any smoking in the child's home(s) or dwelling(s) during infancy or early childhood? (self-reported) | Cotinine measured annually in the child’s serum using the following cut-offs:   - 1 year: 0.11 ng/ml - 2 years: 0.08 ng/ml - 3 years: 0.05 ng/ml - 4 years: 0.04 ng/ml | Any smoking in the child's home(s) or dwelling(s) during the first five years of life (self-reported) | Binary (yes/no) |
| **Season of birth**  **(hospital records)** | - Summer: December-February - Autumn: March-May - Winter: June-august - Spring: September-November | - Summer: June-august - Autumn: September-November - Winter: December-February - Spring: March-May | - Summer: June-august - Autumn: September-November - Winter: December-February - Spring: March-May | - Summer: June-august - Autumn: September-November - Winter: December-February - Spring: March-May | Categorical (Spring, Summer, Autumn, Winter) |

*Abbreviations: BIS: Barwon Infant Study; CHILD: Canadian Healthy Infant Longitudinal Development; ECHO: Environmental Influences on Child Health Outcomes; HOME: Health Outcomes and Measures of the Environment*

# Table S4. Akaike information criteria (AIC) in linear and non-linear joint exposure models

|  | Prenatal exposure | Postnatal Exposure |
| --- | --- | --- |
| Asthma |  |  |
| Polynomial (2^nd^ order) | 3174.88 | 836.79 |
| Polynomial (3^rd^ order) | **3174.40** | 847.79 |
| Restricted cubic spline | 3190.51 | **835.81** |
| Wheeze |  |  |
| Polynomial (2^nd^ order) | 9702.06 | 2980.14 |
| Polynomial (3^rd^ order) | 9702.55 | 2987.59 |
| Restricted cubic spline | **9691.01** | **2977.48** |
| Eczema |  |  |
| Polynomial (2^nd^ order) | 8032.36 | 3601.24 |
| Polynomial (3^rd^ order) | **8009.83** | 3613.21 |
| Restricted cubic spline | 8061.20 | **3592.99** |
| Rhinitis |  |  |
| Polynomial (2^nd^ order) | 5021.37 | 892.55 |
| Polynomial (3^rd^ order) | **5035.46** | 896.44 |
| Restricted cubic spline | 5035.89 | **890.20** |

Bold cells indicate the best fit model

# Table S5. Demographics of all ECHO-wide cohorts

| **N (%) or mean ± SD** | **AAA01**  **N=194** | **AAG01**  **N=114** | **AAM01**  **N=468** | **AAV01**  **N=240** | **AAZ01**  **N=717** | **AAZ02**  **N=234** | **ABA03**  **N=70** | **AFA01**  **N=106** | **AGA01**  **N=410** | **Overall**  **N=2,553** |
| --- | --- | --- | --- | --- | --- | --- | --- | --- | --- | --- |
| **Socio-economic status** |  |  |  |  |  |  |  |  |  |  |
| Marital status |  |  |  |  |  |  |  |  |  |  |
| Single/not married | 20 (10.3) | 103 (90.4) | 67 (14.3) | 35 (14.7) | 302 (42.2) | 26 (11.9) | 3 (4.4) | 2 (1.9) | 61 (15.1) | 619 (24.5) |
| Other | 174 (89.7) | 11 (9.6) | 401 (85.7) | 203 (85.3) | 414 (57.8) | 192 (88.1) | 65 (95.6) | 104 (98.1) | 344 (84.9) | 1,908 (75.5) |
| Missing | 0 | 0 | 0 | 2 | 1 | 16 | 2 | 0 | 5 | 26 |
| Parental Education |  |  |  |  |  |  |  |  |  |  |
| High School or Under | 18 (9.3) | 68 (59.6) | 51 (10.9) | 42 (17.5) | 402 (56.1) | 24 (10.6) | 6 (9.0) | 0 (0.0) | 62 (15.2) | 673 (26.5) |
| Bachelor's Degree | 69 (35.6) | 11 (9.6) | 151 (32.3) | 69 (28.8) | 155 (21.6) | 71 (31.3) | 30 (44.8) | 36 (34.0) | 142 (34.7) | 734 (28.9) |
| Master's or Doctorate | 40 (20.6) | 6 (5.3) | 218 (46.7) | 81 (33.8) | 93 (13.0) | 65 (28.6) | 13 (19.4) | 55 (51.9) | 65 (15.9) | 636 (25.0) |
| Other | 67 (34.5) | 29 (25.4) | 47 (10.1) | 48 (20.0) | 67 (9.3) | 67 (29.5) | 18 (26.9) | 15 (14.2) | 140 (34.2) | 498 (19.6) |
| Missing | 0 | 0 | 1 | 0 | 0 | 7 | 3 | 0 | 1 | 12 |
| Child ethnicity |  |  |  |  |  |  |  |  |  |  |
| Caucasian/White | 72 (37.1) | 0 (0.0) | 340 (72.6) | 199 (82.9) | 242 (33.8) | 189 (80.8) | 55 (78.6) | 93 (87.7) | 186 (45.4) | 1,376 (53.9) |
| Other | 122 (62.9) | 114 (100.0) | 128 (27.4) | 41 (17.1) | 475 (66.2) | 45 (19.2) | 15 (21.4) | 13 (12.3) | 224 (54.6) | 1,177 (46.1) |
| Missing | 0 | 0 | 0 | 0 | 0 | 0 | 0 | 0 | 0 | 0 |
| **Family Characteristics** |  |  |  |  |  |  |  |  |  |  |
| Maternal age (years) | 32.5 ± 4.4 | 26.2 ± 4.8 | 32.1 ± 5.5 | 29.9 ± 5.7 | 27.4 ± 5.5 | 32.2 ± 5.7 | 35.1 ± 5.1 | 31.9 ± 3.4 | 29.2 ± 5.4 | 29.9 ± 5.8 |
| Missing | 0 | 0 | 0 | 0 | 0 | 0 | 0 | 0 | 0 | 0 |
| Family history of asthma | 55 (28.4) | 38 (33.3) | 144 (31.4) | 74 (30.8) | 259 (36.2) | 229 (97.9) | 11 (28.9) | 36 (34.0) | 96 (23.9) | 942 (37.7) |
| Missing | 0 | 0 | 9 | 0 | 2 | 0 | 32 | 0 | 9 | 52 |
| Prenatal smoke exposure | 3 (1.5) | 20 (17.5) | 22 (4.7) | 10 (4.2) | 62 (8.7) | 35 (15.4) | 0 (0.0) | 4 (3.8) | 5 (1.3) | 161 (6.4) |
| Missing | 0 | 0 | 2 | 0 | 1 | 6 | 3 | 0 | 12 | 24 |
| **Child Characteristics** |  |  |  |  |  |  |  |  |  |  |
| Child sex |  |  |  |  |  |  |  |  |  |  |
| Male | 102 (52.6) | 63 (55.3) | 228 (48.7) | 132 (55.0) | 363 (50.6) | 124 (53.0) | 39 (55.7) | 57 (53.8) | 207 (50.5) | 1,315 (51.5) |
| Female | 92 (47.4) | 51 (44.7) | 240 (51.3) | 108 (45.0) | 354 (49.4) | 110 (47.0) | 31 (44.3) | 49 (46.2) | 203 (49.5) | 1,238 (48.5) |
| Missing | 0 | 0 | 0 | 0 | 0 | 0 | 0 | 0 | 0 | 0 |
| Season of birth |  |  |  |  |  |  |  |  |  |  |
| Spring | 57 (29.4) | 35 (30.7) | 120 (25.6) | 62 (25.8) | 201 (28.0) | 47 (20.1) | 23 (32.9) | 21 (19.8) | 94 (22.9) | 660 (25.9) |
| Summer | 50 (25.8) | 26 (22.8) | 122 (26.1) | 53 (22.1) | 167 (23.3) | 65 (27.8) | 21 (30.0) | 25 (23.6) | 102 (24.9) | 631 (24.7) |
| Autumn | 47 (24.2) | 24 (21.1) | 122 (26.1) | 63 (26.2) | 168 (23.4) | 60 (25.6) | 9 (12.9) | 33 (31.1) | 97 (23.7) | 623 (24.4) |
| Winter | 40 (20.6) | 29 (25.4) | 104 (22.2) | 62 (25.8) | 181 (25.2) | 62 (26.5) | 17 (24.3) | 27 (25.5) | 117 (28.5) | 639 (25.0) |
| Missing | 0 | 0 | 0 | 0 | 0 | 0 | 0 | 0 | 0 | 0 |
| Gestational age (weeks | 38.9 ± 1.8 | 38.0 ± 2.4 | 39.0 ± 2.2 | 39.0 ± 1.4 | 38.7 ± 1.8 | 38.2 ± 2.4 | 38.7 ± 2.0 | 38.7 ± 1.3 | 38.2 ± 2.2 | 38.6 ± 2.0 |
| Missing | 0 | 0 | 0 | 0 | 0 | 0 | 0 | 0 | 0 | 0 |
| Breastfeeding duration (weeks) | 58.5 ± 45.3 | 18.0 ± 20.5 | 1.1 ± 5.8 | 36.1 ± 24.5 | - | - | - | 44.8 ± 31.7 | 12.4 ± 14.8 | 24.7 ± 32.7 |
| Missing | 63 | 49 | 226 | 38 | 716 | 234 | 67 | 77 | 322 | 1,792 |
| Postnatal smoke exposure | 27 (14.1) | 5 (62.5) | 36 (8.8) | 44 (18.3) | 237 (33.1) | 28 (12.0) | 3 (8.6) | 3 (4.2) | 54 (13.7) | 437 (19.0) |
| Missing | 2 | 106 | 57 | 0 | 0 | 1 | 35 | 34 | 16 | 251 |
| Child outcomes |  |  |  |  |  |  |  |  |  |  |
| Asthma | 26 (13.4) | 9 (9.2) | 33 (7.1) | 17 (7.1) | 116 (16.2) | 29 (12.4) | - | - | 18 (10.8) | 248 (11.7) |
| Missing | 0 | 16 | 0 | 0 | 0 | 0 | 70 | 106 | 243 | 435 |
| Wheeze | 55 (28.4) | 26 (22.8) | 83 (17.7) | 12 (5.0) | 260 (36.3) | 74 (31.6) | 32 (45.7) | 13 (12.3) | 157 (38.3) | 712 (27.9) |
| Missing | 0 | 0 | 0 | 0 | 0 | 0 | 0 | 0 | 0 | 0 |
| Eczema | 78 (40.2) | 35 (35.4) | 163 (34.9) | 71 (29.6) | 263 (38.7) | 103 (44.0) | - | 32 (45.1) | 45 (25.1) | 790 (36.5) |
| Missing | 0 | 15 | 1 | 0 | 37 | 0 | 70 | 35 | 231 | 389 |
| Rhinitis | 123 (63.4) | 42 (42.4) | - | 14 (5.8) | 219 (32.2) | 79 (33.8) | - | 38 (55.9) | 77 (46.1) | 592 (35.2) |
| Missing | 0 | 15 | 468 | 0 | 36 | 0 | 70 | 38 | 243 | 870 |

*Proportions exclude missing data.*

*Abbreviations: ECHO: Environmental Influences on Child Health Outcomes*

# Table S6. Average exposure estimated daily intakes distribution across cohorts (µg/kg/day).

|  | DMP | DEP | DBP | BBzP | DEHP | BPA | BPS | BPF |
| --- | --- | --- | --- | --- | --- | --- | --- | --- |
| BIS |  |  |  |  |  |  |  |  |
| GM (GSD) | 0.05 (2.45) | 1.58 (3.82) | 1.87 (1.96) | 0.18 (2.94) | 1.61 (2.08) | 0.01 (7.91) | 0.001 (6.45) | 0.0003 (16.14) |
| Median (IQR) | 0.05 (0.05) | 1.36 (2.69) | 1.86 (1.70) | 0.18 (0.25) | 1.54 (1.28) | 0.01 (0.04) | 0.001 (0.003) | 0.0002 (0.002) |
| CHILD |  |  |  |  |  |  |  |  |
| GM (GSD) |  | 1.04 (2.46) | 2.30 (1.91) | 0.35 (2.90) | 5.02 (2.06) |  |  |  |
| Median (IQR) |  | 0.98 (1.05) | 2.22 (1.81) | 0.32 (0.52) | 5.17 (4.57) |  |  |  |
| HOME (Prenatal) |  |  |  |  |  |  |  |  |
| GM (GSD) |  | 5.67 (3.44) | 1.39 (2.34) | 0.37 (3.09) | 15.12 (3.27) | 0.05 (2.31) |  |  |
| Median (IQR) |  | 5.73 (10.33) | 1.37 (1.53) | 0.37 (0.52) | 12.47 (18.77) | 0.05 (0.06) |  |  |
| HOME (Postnatal) |  |  |  |  |  |  |  |  |
| GM (GSD) |  | 3.10 (2.92) | 2.13 (2.40) | 0.96 (2.30) | 25.08 (1.93) | 0.17 (2.13) |  |  |
| Median (IQR) |  | 2.73 (4.64) | 2.14 (2.46) | 0.91 (1.01) | 24.86 (20.63) | 0.16 (0.16) |  |  |
| AAA01 ^a^ |  |  |  |  |  |  |  |  |
| GM (GSD) |  |  |  |  |  | 0.02 (2.59) | 0.02 (3.21) | 0.01 (3.02) |
| Median (IQR) |  |  |  |  |  | 0.02 (0.02) | 0.02 (0.02) | 0.01 (0.02) |
| AAG01 ^a^ |  |  |  |  |  |  |  |  |
| GM (GSD) |  | 0.23 (22.91) | 0.11 (9.88) | 0.05 (5.75) | 0.79 (4.87) | 0.02 (4.16) |  |  |
| Median (IQR) |  | 0.61 (2.64) | 0.18 (0.91) | 0.02 (0.18) | 0.57 (3.11) | 0.02 (0.03) |  |  |
| AAM01 ^a^ |  |  |  |  |  |  |  |  |
| GM (GSD) |  | 0.88 (4.92) | 0.48 (2.87) | 0.17 (5.22) | 3.29 (3.05) | 0.01 (4.60) | 0.01 (2.58) | 0.02 (2.79) |
| Median (IQR) |  | 0.79 (2.17) | 0.48 (0.76) | 0.20 (0.37) | 3.12 (5.07) | 0.02 (0.04) | 0.01 (0.01) | 0.02 (0.02) |
| AAV01 ^a^ |  |  |  |  |  |  |  |  |
| GM (GSD) | 0.04 (3.26) | 1.25 (5.38) | 0.57 (3.57) | 0.17 (4.28) | 3.35 (2.94) | 0.02 (2.36) | 0.01 (2.79) |  |
| Median (IQR) | 0.04 (0.07) | 0.99 (2.47) | 0.66 (1.12) | 0.17 (0.39) | 3.27 (4.85) | 0.02 (0.03) | 0.01 (0.01) |  |
| AAZ01 ^a^ |  |  |  |  |  |  |  |  |
| GM (GSD) | 0.07 (7.05) | 3.50 (3.97) | 1.19 (2.56) | 0.48 (3.76) | 8.45 (3.18) |  |  |  |
| Median (IQR) | 0.09 (0.33) | 3.26 (7.19) | 1.27 (1.65) | 0.50 (0.81) | 8.39 (12.85) |  |  |  |
| AAZ02 ^a^ |  |  |  |  |  |  |  |  |
| GM (GSD) | 0.03 (5.14) | 0.85 (5.97) | 0.58 (4.05) | 0.27 (4.83) | 3.77 (3.39) |  |  |  |
| Median (IQR) | 0.04 (0.10) | 0.96 (1.92) | 0.68 (1.21) | 0.29 (0.60) | 3.89 (6.23) |  |  |  |
| ABA03 ^a^ |  |  |  |  |  |  |  |  |
| GM (GSD) |  | 1.18 (3.62) | 0.86 (2.76) | 0.25 (3.40) | 9.72 (3.14) | 0.02 (2.60) | 0.01 (2.77) | 0.01 (3.78) |
| Median (IQR) |  | 0.99 (2.71) | 0.92 (1.37) | 0.26 (0.39) | 10.20 (11.11) | 0.02 (0.03) | 0.01 (0.01) | 0.01 (0.02) |
| AFA01 ^a^ |  |  |  |  |  |  |  |  |
| GM (GSD) |  | 0.82 (2.58) | 0.85 (2.39) | 0.16 (2.97) | 2.97 (2.32) | 0.02 (2.79) | 0.01 (2.57) | 0.01 (4.33) |
| Median (IQR) |  | 0.82 (1.12) | 0.83 (0.80) | 0.14 (0.21) | 2.52 (3.10) | 0.02 (0.02) | 0.01 (0.01) | 0.01 (0.01) |
| AGA01 ^a^ |  |  |  |  |  |  |  |  |
| GM (GSD) |  | 0.60 (6.94) | 0.51 (4.80) | 0.05 (4.01) | 3.16 (3.33) | 0.03 (3.14) | 0.02 (2.81) | 0.01 (3.04) |
| Median (IQR) |  | 0.51 (1.95) | 0.74 (1.32) | 0.06 (0.12) | 3.80 (5.23) | 0.03 (0.05) | 0.02 (0.02) | 0.01 (0.01) |

*^a^: ECHO-wide cohorts*

*Abbreviations: BBzP: Benzyl butyl phthalate; BIS: Barwon Infant Study; BPA: Bisphenol A; BPF: Bisphenol F; BPS: Bisphenol S; CHILD: Canadian Healthy Infant Longitudinal Development; DBP: Dibutyl phthalate; DEHP: Di(2-ethylhexyl) phthalate; DEP: Diethyl phthalate; DMP: Dimethyl phthalate; ECHO: Environmental Influences on Child Health Outcomes; GM: Geometric mean; GSD: Geometric standard deviation; HOME: Health Outcomes and Measures of the Environment; IQR: Interquartile range.*

# Table S7. Average plasticiser metabolite distribution across cohorts (µg/L).

|  | MMP | MEP | MBP | MBzP | MEHP | MEHHP | MEOHP | MECPP | MCPP | BPA | BPS | BPF |  |
| --- | --- | --- | --- | --- | --- | --- | --- | --- | --- | --- | --- | --- | --- |
| BIS | | | | | | | | | | | | | |
| Samples (N) | 833 | 833 | 833 | 833 |  | 833 | 833 | 833 | 833 | 833 | 833 | 833 |  |
| % > LOD | 98.6 | 99.4 | 91.4 | 98.1 |  | 99.9 | 100 | 100 | 80.3 | 54.0 | 34.8 | 15.8 |  |
| GM (GSD) | 1.56 (2.39) | 46.95 (3.73) | 51.47 (1.92) | 5.28 (2.91) |  | 11.24 (2.20) | 9.12 (2.13) | 13.66 (1.99) | 1.01 (5.95) | 0.65 (3.99) | 0.05 (2.78) | 0.24 (2.16) |  |
| Median (IQR) | 1.52 (1.48) | 39.55 (73.72) | 51.69 (41.68) | 5.33 (7.18) |  | 11.29 (9.00) | 8.94 (7.23) | 13.02 (10.08) | 1.40 (2.44) | 0.68 (2.00) | 0.03 (0.05) | 0.18 (0.00) |  |
| CHILD | | | | | | | | | | | | | |
| Samples (N) | 1933 | 3421 | 3456 | 3454 | 3457 | 3423 | 3455 |  | 3448 |  |  |  |  |
| % > LOD | 22.7 ^a^ | 93.4 | 99.5 | 78.1 | 59.8 | 84.8 | 82.3 |  | 59.9 |  |  |  |  |
| GM (GSD) | 0.66 (4.64) | 11.76 (2.87) | 22.49 (2.03) | 4.25 (3.43) | 0.52 (4.00) | 4.71 (2.91) | 3.54 (2.71) |  | 0.99 (2.26) |  |  |  |  |
| Median (IQR) | 0.63 (1.57) | 10.84 (13.61) | 21.98 (20.73) | 3.98 (7.18) | 0.54 (1.06) | 5.49 (6.83) | 4.00 (4.66) |  | 1.08 (1.01) |  |  |  |  |
| HOME (Prenatal) | | | | | | | | | | | | | |
| Samples (N) |  | 740 | 740 | 740 | 740 | 740 | 740 | 740 | 740 | 740 |  |  |  |
| % > LOD |  | 100 | 95 | 98.9 | 76.9 | 99.2 | 99.1 | 100 | 97.2 | 90.4 |  |  |  |
| GM (GSD) |  | 151.77 (3.41) | 33.65 (2.35) | 9.75 (3.10) | 5.55 (3.82) | 30.08 (3.53) | 22.20 (3.44) | 42.67 (3.10) | 2.79 (2.10) | 2.09 (2.35) |  |  |  |
| Median (IQR) |  | 154.95 (256.24) | 34.41 (37.14) | 10.00 (15.53) | 4.68 (7.71) | 24.72 (41.97) | 18.47 (28.35) | 34.88 (52.86) | 2.83 (2.61) | 1.89 (2.00) |  |  |  |
| HOME (Postnatal) | | | | | | | | | | | | | |
| Samples (N) |  | 878 | 174 | 878 | 174 ^b^ | 878 | 878 | 878 | 878 | 879 |  |  |  |
| % > LOD |  | 100 | 100 | 99.7 | 77 | 99.7 | 99.0 | 100 | 99.5 | 96.7 |  |  |  |
| GM (GSD) |  | 53.56 (3.38) | 58.12 (1.47) | 16.91 (2.63) | 3.56 (1.52) | 26.51 (2.4) | 17.66 (2.33) | 55.13 (2.13) | 5.87 (1.94) | 4.15 (2.27) |  |  |  |
| Median (IQR) |  | 47.51 (87.65) | 49.92 (23.37) | 15.29 (18.61) | 3.71 (1.50) | 25.99 (27.24) | 17.19 (17.64) | 53.92 (54.04) | 5.94 (5.2) | 4.09 (4.43) |  |  |  |
| AAA01 | | | | | | | | | | | | | |
| Samples (N) |  |  |  |  |  |  |  |  |  | 383 | 383 | 383 |  |
| % > LOD |  |  |  |  |  |  |  |  |  | 87.7 | 56.9 | 23.5 |  |
| GM (GSD) |  |  |  |  |  |  |  |  |  | 0.75 (2.56) | 0.61 (2.86) | 0.51 (3.32) |  |
| Median (IQR) |  |  |  |  |  |  |  |  |  | 0.73 (0.98) | 0.60 (0.85) | 0.51 (0.75) |  |
| AAG01 |  |  |  |  |  |  |  |  |  |  |  |  |  |
| Samples (N) | 266 | 266 | 266 | 266 | 266 | 266 | 266 | 266 | 266 | 266 | 266 | 266 |  |
| % > LOD | 0.4 ^a^ | 57.1 | 53.8 | 56.0 | 51.5 | 56.0 | 56.0 | 57.1 | 42.5 | 75.6 | 0.8 ^a^ | 4.5 ^a^ |  |
| GM (GSD) | 0.06 (1.65) | 6.66 (23.77) | 4.01 (10.15) | 1.83 (5.72) | 0.45 (5.10) | 1.14 (4.51) | 1.07 (4.08) | 1.23 (7.26) | 0.35 (2.82) | 0.87 (3.29) | 0.2 (2.6) | 0.02 (12.4) |  |
| Median (IQR) | 0.06 (0.03) | 24.67 (92.84) | 10.07 (31.55) | 1.51 (6.43) | 0.20 (1.77) | 1.79 (3.98) | 1.15 (3.13) | 3.00 (6.38) | 0.22 (0.73) | 0.93 (1.15) | 0.19 (0.28) | 0.03 (0.1) |  |
| AAM01 |  |  |  |  |  |  |  |  |  |  |  |  |  |
| Samples (N) | 1,363 | 1,363 | 1,363 | 1,363 | 1,363 | 1,363 | 1,363 | 1,363 | 1,363 | 1,363 | 1363 | 1,363 |  |
| % > LOD | 48.7 ^a^ | 99.9 | 99.4 | 95.6 | 76.9 | 99.9 | 100 | 100 | 88.6 | 68.3 | 53.0 | 40.0 |  |
| GM (GSD) | 0.22 (14.89) | 26.21 (4.98) | 13.78 (2.91) | 4.83 (5.26) | 1.36 (4.57) | 5.52 (3.25) | 3.50 (3.12) | 7.63 (3.06) | 1.58 (3.70) | 0.50 (4.64) | 0.44 (2.6) | 0.79 (3.06) |  |
| Median (IQR) | 0.02 (2.85) | 23.18 (65.17) | 13.60 (21.91) | 5.76 (10.37) | 1.47 (4.25) | 4.99 (8.84) | 3.24 (5.49) | 7.72 (11.26) | 1.61 (2.53) | 0.61 (1.49) | 0.44 (0.54) | 0.78 (0.93) |  |
| AAV01 |  |  |  |  |  |  |  |  |  |  |  |  |  |
| Samples (N) | 240 | 240 | 240 | 240 | 240 | 240 | 240 | 240 | 240 | 240 | 240 |  |  |
| % > LOD | 67.5 | 100 | 94.6 | 96.7 | 68.3 | 98.8 | 98.8 | 100 | 96.7 | 97.5 | 83.8 |  |  |
| GM (GSD) | 1.26 (3.23) | 34.54 (5.35) | 14.67 (3.69) | 4.61 (4.39) | 1.05 (2.81) | 3.99 (3.68) | 3.81 (3.40) | 9.36 (3.12) | 1.94 (3.63) | 0.96 (2.40) | 0.30 (2.78) |  |  |
| Median (IQR) | 1.20 (2.25) | 28.20 (62.03) | 17.75 (29.83) | 4.50 (10.35) | 0.90 (1.57) | 4.15 (8.03) | 3.90 (7.00) | 10.15 (14.88) | 1.85 (3.50) | 0.90 (1.10) | 0.30 (0.45) |  |  |
| AAZ01 |  |  |  |  |  |  |  |  |  |  |  |  |  |
| Samples (N) | 1,378 | 1,378 | 1,378 | 1,378 | 1,378 | 1,378 | 1,378 | 1,378 | 1,378 |  |  |  |  |
| % > LOD | 81.2 | 99.9 | 99.9 | 99.1 | 90.6 | 99.6 | 99.9 | 99.9 | 99.9 |  |  |  |  |
| GM (GSD) | 2.12 (7.04) | 105.47 (3.96) | 33.10 (2.61) | 14.52 (3.80) | 4.24 (4.88) | 17.21 (3.62) | 9.92 (2.98) | 14.90 (2.80) | 1.77 (2.80) |  |  |  |  |
| Median (IQR) | 3.10 (10.35) | 96.80 (215.62) | 34.84 (48.63) | 15.30 (26.47) | 5.40 (9.35) | 18.06 (32.18) | 9.31 (15.57) | 13.90 (20.41) | 1.68 (2.30) |  |  |  |  |
| AAZ02 |  |  |  |  |  |  |  |  |  |  |  |  |  |
| Samples (N) | 234 | 234 | 234 | 234 | 234 | 234 | 234 | 234 | 234 |  |  |  |  |
| % > LOD | 88.5 | 98.7 | 97.9 | 98.7 | 84.2 | 99.6 | 99.6 | 99.6 | 90.6 |  |  |  |  |
| GM (GSD) | 1.30 (5.00) | 27.18 (5.99) | 17.75 (4.13) | 8.38 (5.06) | 1.05 (7.25) | 7.38 (3.62) | 4.42 (3.37) | 10.95 (3.42) | 1.64 (4.50) |  |  |  |  |
| Median (IQR) | 1.66 (3.70) | 29.24 (68.11) | 21.75 (35.99) | 9.98 (17.87) | 1.36 (3.22) | 7.97 (12.61) | 4.45 (7.03) | 11.58 (20.88) | 1.88 (4.03) |  |  |  |  |
| ABA03 |  |  |  |  |  |  |  |  |  |  |  |  |  |
| Samples (N) |  | 153 | 153 | 153 | 153 | 153 | 153 | 153 | 153 | 153 | 153 | 153 |  |
| % > LOD |  | 100 | 98.0 | 98.7 | 85.6 | 100 | 100 | 100 | 90.8 | 92.8 | 22.2 | 47.1 |  |
| GM (GSD) |  | 34.01 (3.68) | 22.73 (2.75) | 6.98 (3.50) | 3.21 (3.53) | 15.69 (3.35) | 12.43 (3.13) | 24.93 (2.99) | 2.30 (3.27) | 0.96 (2.61) | 0.31 (2.40) | 0.27 (5.09) |  |
| Median (IQR) |  | 25.83 (73.73) | 27.85 (39.23) | 6.50 (10.33) | 3.05 (5.25) | 15.53 (16.35) | 12.63 (14.75) | 24.40 (33.23) | 2.15 (3.27) | 0.84 (1.18) | 0.28 (0.36) | 0.24 (0.58) |  |
| AFA01 |  |  |  |  |  |  |  |  |  |  |  |  |  |
| Samples (N) |  | 106 | 106 | 106 | 106 | 106 | 106 | 106 | 106 | 106 | 106 | 106 |  |
| % > LOD |  | 100 | 100 | 100 | 60.4 | 100 | 100 | 100 | 90.6 | 97.2 | 96.2 | 51.9 |  |
| GM (GSD) |  | 23.79 (2.49) | 22.76 (2.23) | 4.49 (2.88) | 1.22 (2.35) | 4.58 (2.56) | 3.58 (2.49) | 7.36 (2.21) | 1.12 (2.64) | 0.71 (2.78) | 0.46 (2.49) | 0.49 (3.64) |  |
| Median (IQR) |  | 22.80 (28.83) | 21.40 (18.68) | 4.05 (5.70) | 1.10 (1.33) | 4.00 (5.68) | 3.00 (4.53) | 6.80 (7.15) | 1.00 (0.90) | 0.70 (1.08) | 0.40 (0.40) | 0.43 (0.46) |  |
| AGA01 | | | | | | | | | | | | | |
| Samples (N) | 933 | 933 | 933 | 933 | 933 | 933 | 933 | 933 | 933 | 933 | 933 | 933 |  |
| % > LOD | 0.6 ^a^ | 88.3 | 86.7 | 83.2 | 72.1 | 88.7 | 88.9 | 88.9 | 71.4 | 91.1 | 86.7 | 45.1 |  |
| GM (GSD) | 0.07 (1.52) | 15.84 (6.93) | 12.32 (4.88) | 1.42 (4.07) | 1.67 (2.72) | 3.46 (4.08) | 2.89 (4.88) | 5.66 (4.62) | 0.75 (2.81) | 1.23 (3.20) | 0.59 (2.88) | 0.39 (3.26) |  |
| Median (IQR) | 0.07 (0.00) | 14.00 (55.13) | 17.70 (31.75) | 1.50 (3.10) | 1.56 (2.71) | 4.80 (7.15) | 4.40 (6.50) | 8.30 (11.35) | 0.65 (1.12) | 1.30 (1.90) | 0.50 (0.70) | 0.40 (0.47) |  |

*Metabolites corrected for specific gravity*

*^a^ Excluded due to insufficient detection rate*

*^b^ Excluded due to external contamination*

*Abbreviations: BPA: Bisphenol A; BPF: Bisphenol F; BPS: Bisphenol S; IQR: Interquartile range; MBP: Monobutyl phthalate; MBzP: Monobenzyl phthalate; MCPP: Mono(3-carboxypropyl) phthalate; MECPP: Mono(2-ethyl-5-carboxypentyl) phthalate; MEHHP: Mono(2-ethyl-5-hydroxyhexyl) phthalate; MEHP: Mono(2-ethylhexyl) phthalate; MEOHP: Mono(2-ethyl-5-oxohexyl) phthalate; MEP: Monoethyl phthalate; MMP: Monomethyl phthalate*

# Table S8. Crude risk ratios of allergic conditions associated with prenatal exposure to phthalates and bisphenols.

|  | Asthma | | | Wheeze | | |
| --- | --- | --- | --- | --- | --- | --- |
|  | Cohorts | N | RR (95%CI) | Cohorts | N | RR (95%CI) |
| DMP | 4 | 1927 | 1.03 (0.96-1.09) | 4 | 1983 | 1.08 (1.03-1.13) |
| DEP | 8 | 2900 | 1.06 (1.02-1.10) | 10 | 3447 | 1.02 (0.99-1.04) |
| DBP | 8 | 2900 | 1.17 (1.10-1.25) | 10 | 3447 | 1.02 (0.99-1.06) |
| BBzP | 8 | 2900 | 1.11 (1.06-1.17) | 10 | 3447 | 1.04 (1.01-1.07) |
| DEHP | 8 | 2900 | 1.03 (0.98-1.08) | 10 | 3447 | 1.07 (1.04-1.10) |
| MCPP | 7 | 2812 | 1.02 (0.97-1.08) | 9 | 3344 | 1.03 (1.01-1.06) |
| BPA | 7 | 2163 | 0.98 (0.93-1.03) | 9 | 2710 | 1.03 (1.00-1.05) |
| BPS | 5 | 1808 | 0.93 (0.90-0.97) | 7 | 2231 | 1.05 (1.02-1.07) |
| BPF | 4 | 1570 | 0.98 (0.95-1.01) | 6 | 1993 | 1.04 (1.03-1.06) |
|  | Eczema | | | Rhinitis | | |
|  | Cohorts | N | RR (95%CI) | Cohorts | N | RR (95%CI) |
| DMP | 4 | 1947 | 1.05 (1.00-1.11) | 4 | 1869 | 1.11 (1.04-1.18) |
| DEP | 9 | 3111 | 1.04 (1.02-1.07) | 8 | 2562 | 1.03 (1.00-1.06) |
| DBP | 9 | 3111 | 0.92 (0.90-0.95) | 8 | 2562 | 0.97 (0.93-1.00) |
| BBzP | 9 | 3111 | 1.07 (1.04-1.11) | 8 | 2562 | 1.05 (1.01-1.08) |
| DEHP | 9 | 3111 | 1.12 (1.10-1.15) | 8 | 2562 | 1.13 (1.10-1.17) |
| MCPP | 8 | 3023 | 1.09 (1.06-1.12) | 7 | 2474 | 1.07 (1.04-1.11) |
| BPA | 8 | 2409 | 1.09 (1.06-1.12) | 7 | 1860 | 1.10 (1.07-1.14) |
| BPS | 6 | 1945 | 1.16 (1.13-1.20) | 5 | 1396 | 1.19 (1.15-1.24) |
| BPF | 5 | 1707 | 1.14 (1.11-1.16) | 4 | 1158 | 1.17 (1.13-1.20) |

*Models obtained with generalised estimating equations. All exposures are modelled as estimated daily intakes, except MCPP, which is modelled using biomarker concentrations. Abbreviations: BBzP: Benzyl butyl phthalate; BPA: Bisphenol A; BPF: Bisphenol F; BPS: Bisphenol S; DBP: Dibutyl phthalate; DEHP: Di(2-ethylhexyl) phthalate; DEP: Diethyl phthalate; DMP: Dimethyl phthalate; MCPP: Mono(3-carboxypropyl) phthalate; RR: Risk Ratio*

|  | Asthma | | | | Wheeze | | | |
| --- | --- | --- | --- | --- | --- | --- | --- | --- |
|  | Cohorts | N | RR (95%CI) | Cohorts | | N | RR (95%CI) |  |
| DEP | 2 | 1175 | 0.93 (0.88-1.00) | 2 | | 1719 | 1.04 (1.00-1.07) |  |
| DBP | 2 | 1098 | 1.10 (0.98-1.22) | 2 | | 1589 | 1.04 (0.97-1.10) |  |
| BBzP | 2 | 1184 | 0.97 (0.91-1.04) | 2 | | 1720 | 1.06 (1.03-1.12) |  |
| DEHP | 2 | 1179 | 0.99 (0.90-1.08) | 2 | | 1718 | 1.07 (1.03-1.12) |  |
| MCPP | 2 | 1184 | 1.09 (0.99-1.19) | 2 | | 1719 | 1.07 (1.03-1.11) |  |
| BPA | 1 | 235 | 0.93 (0.83-1.06) | 1 | | 303 | 1.07 (0.99-1.14) |  |
|  | Eczema | | | | Rhinitis | | | |
|  | Cohorts | N | RR (95%CI) | Cohorts | | N | RR (95%CI) |  |
| DEP | 2 | 1707 | 1.04 (1.01-1.06) | 2 | | 1273 | 1.02 (0.96-1.09) |  |
| DBP | 2 | 1576 | 1.05 (1.00-1.10) | 2 | | 1149 | 0.98 (0.87-1.10) |  |
| BBzP | 2 | 1707 | 0.97 (0.94-0.99) | 2 | | 1280 | 1.04 (0.97-1.11) |  |
| DEHP | 2 | 1705 | 0.94 (0.91-0.97) | 2 | | 1276 | 1.05 (0.96-1.15) |  |
| MCPP | 2 | 1707 | 0.92 (0.89-0.94) | 2 | | 1279 | 1.10 (1.02-1.19) |  |
| BPA | 1 | 303 | 1.05 (0.99-1.10) | 1 | | 303 | 1.01 (0.97-1.06) |  |

# Table S9. Crude risk ratios of allergic conditions associated with postnatal exposure to phthalates and bisphenols.

*Models obtained with generalised estimating equations. All exposures are modelled as estimated daily intakes, except MCPP, which is modelled using biomarker concentrations. Abbreviations: BBzP: Benzyl butyl phthalate; BPA: Bisphenol A; DBP: Dibutyl phthalate; DEHP: Di(2-ethylhexyl) phthalate; DEP: Diethyl phthalate; DMP: Dimethyl phthalate; MCPP: Mono(3-carboxypropyl) phthalate; RR: Risk Ratio*

|  | Asthma | | | | Wheeze | | |
| --- | --- | --- | --- | --- | --- | --- | --- |
|  | Male | Female | p_int_ | Male | | Female | p_int_ |
| DMP | 0.99 (0.91-1.07) | 0.95 (0.86-1.04) | 0.44 | 1.02 (0.98-1.07) | | 0.97 (0.92-1.02) | 0.13 |
| DEP | 1.03 (0.97-1.10) | 1.01 (0.96-1.06) | 0.52 | 0.99 (0.96-1.03) | | 1.01 (0.98-1.04) | 0.46 |
| DBP | 1.05 (0.95-1.14) | 1.12 (1.00-1.24) | 0.30 | 1.03 (0.98-1.08) | | 1.05 (1.00-1.11) | 0.50 |
| BBzP | 1.06 (0.98-1.14) | 1.06 (0.98-1.14) | 0.99 | 1.01 (0.98-1.05) | | 1.01 (0.98-1.05) | 0.98 |
| DEHP | 0.98 (0.90-1.06) | 1.03 (0.95-1.11) | 0.31 | 0.99 (0.95-1.02) | | 1.03 (0.99-1.08) | 0.06 |
| MCPP | 0.99 (0.93-1.06) | 1.07 (0.99-1.15) | 0.14 | 1.02 (0.99-1.06) | | 1.03 (0.99-1.07) | 0.88 |
| BPA | 0.96 (0.90-1.02) | 1.04 (0.96-1.12) | 0.09 | 0.99 (0.96-1.02) | | 1.01 (0.97-1.04) | 0.36 |
| BPS | 0.96 (0.90-1.02) | 1.01 (0.94-1.08) | 0.17 | 0.99 (0.96-1.02) | | 1.01 (0.98-1.05) | 0.23 |
| BPF | 1.04 (1.00-1.09) | 1.01 (0.95-1.06) | 0.23 | 1.00 (0.98-1.02) | | 1.00 (0.98-1.03) | 0.98 |
|  | Eczema | | | | Rhinitis | | |
|  | Male | Female | p_int_ | Male | | Female | p_int_ |
| DMP | 0.97 (0.92-1.02) | 0.99 (0.94-1.04) | 0.51 | 0.98 (0.91-1.04) | | 1.04 (0.97-1.10) | 0.19 |
| DEP | 1.01 (0.98-1.05) | 1.02 (0.99-1.05) | 0.86 | 1.01 (0.98-1.05) | | 0.99 (0.96-1.02) | 0.35 |
| DBP | 1.00 (0.95-1.05) | 1.01 (0.96-1.06) | 0.59 | 1.01 (0.96-1.06) | | 1.02 (0.97-1.07) | 0.78 |
| BBzP | 1.00 (0.96-1.04) | 1.00 (0.95-1.04) | 0.98 | 1.00 (0.96-1.05) | | 1.02 (0.97-1.06) | 0.62 |
| DEHP | 0.96 (0.92-1.00) | 0.97 (0.93-1.02) | 0.68 | 1.01 (0.96-1.06) | | 1.05 (1.00-1.10) | 0.20 |
| MCPP | 1.01 (0.97-1.06) | 1.04 (0.99-1.08) | 0.41 | 1.03 (0.98-1.09) | | 1.06 (1.00-1.12) | 0.49 |
| BPA | 1.00 (0.96-1.04) | 0.98 (0.94-1.02) | 0.55 | 1.02 (0.97-1.07) | | 1.01 (0.95-1.07) | 0.87 |
| BPS | 0.97 (0.93-1.02) | 0.98 (0.94-1.02) | 0.83 | 1.01 (0.95-1.07) | | 1.00 (0.94-1.06) | 0.68 |
| BPF | 1.00 (0.97-1.03) | 1.02 (0.98-1.05) | 0.38 | 1.03 (0.98-1.08) | | 1.02 (0.97-1.08) | 0.85 |

# Table S10. Adjusted risk ratios of allergic conditions associated with a twofold increase in prenatal phthalates and bisphenols, stratified by child’s sex

*Models obtained with generalised estimating equations. P-values are shown for the multiplicative interaction term between the exposure and the child’s sex. Risk ratios are shown for a twofold increase in phthalates and bisphenols. P-values are shown for the multiplicative interaction term between the exposure and the child’s sex. Models adjusted for cohort, maternal age, ethnicity, parental education, lone parenthood, family history of asthma, prenatal tobacco smoke exposure, and season of birth. All exposures are modelled as estimated daily intakes, except MCPP, which is modelled using biomarker concentrations.*

*Abbreviations: BBzP: Benzyl butyl phthalate; BPA: Bisphenol A; DBP: Dibutyl phthalate; DEHP: Di(2-ethylhexyl) phthalate; DEP: Diethyl phthalate; DMP: Dimethyl phthalate; MCPP: Mono(3-carboxypropyl) phthalate; RR: Risk ratios; p_int_: p-value for interaction*

|  | Asthma | | | Wheeze | | |
| --- | --- | --- | --- | --- | --- | --- |
|  | Male | Female | P_int_ | Male | Female | P_int_ |
| DEP | 0.96 (0.85-1.07) | 0.96 (0.86-1.06) | 0.96 | 0.97 (0.92-1.03) | 1.01 (0.95-1.07) | 0.32 |
| DBP | 1.22 (1.01-1.44) | 1.02 (0.83-1.20) | 0.15 | 1.01 (0.93-1.08) | 1.06 (0.94-1.18) | 0.46 |
| BBzP | 0.96 (0.86-1.06) | 0.89 (0.81-0.98) | 0.32 | 1.05 (1.00-1.09) | 1.05 (0.99-1.11) | 0.86 |
| DEHP | 0.94 (0.83-1.05) | 1.07 (0.94-1.21) | 0.10 | 1.03 (0.97-1.08) | 1.11 (1.04-1.18) | 0.04 |
| MCPP | 1.00 (0.86-1.14) | 1.12 (0.95-1.28) | 0.24 | 1.07 (1.01-1.12) | 1.14 (1.06-1.21) | 0.09 |
| BPA | 0.96 (0.76-1.16) | 0.84 (0.71-0.97) | 0.26 | 0.94 (0.84-1.03) | 1.02 (0.90-1.14) | 0.27 |
|  | Eczema | | | Rhinitis | | |
|  | Male | Female | P_int_ | Male | Female | P_int_ |
| DEP | 1.02 (0.98-1.05) | 0.98 (0.94-1.02) | 0.14 | 0.99 (0.93-1.06) | 1.00 (0.95-1.06) | 0.83 |
| DBP | 1.04 (0.98-1.10) | 0.98 (0.92-1.05) | 0.21 | 1.02 (0.86-1.18) | 1.07 (0.91-1.22) | 0.67 |
| BBzP | 0.99 (0.96-1.02) | 0.96 (0.92-1.00) | 0.21 | 0.97 (0.91-1.03) | 1.02 (0.97-1.08) | 0.16 |
| DEHP | 0.95 (0.92-0.99) | 0.93 (0.89-0.98) | 0.43 | 0.94 (0.87-1.00) | 1.05 (0.98-1.12) | 0.02 |
| MCPP | 0.97 (0.93-1.00) | 0.93 (0.89-0.97) | 0.14 | 0.93 (0.85-1.01) | 1.04 (0.97-1.11) | 0.04 |
| BPA | 1.03 (0.96-1.10) | 0.97 (0.89-1.05) | 0.29 | 1.05 (0.98-1.13) | 1.01 (0.95-1.08) | 0.39 |

# Table S11. Adjusted risk ratios of allergic conditions associated with a twofold increase in postnatal phthalates and bisphenols, stratified by child’s sex

*Models obtained with generalised estimating equations. P-values are shown for the multiplicative interaction term between the exposure and the child’s sex. Risk ratios are shown for a twofold increase in phthalates and bisphenols. Models adjusted for the cohort, maternal age, ethnicity, parental education, lone parenthood, family history of asthma, prenatal tobacco smoke exposure, season of birth, breastfeeding duration, age at outcome assessment, postnatal smoke exposure and gestational age. All exposures are modelled as estimated daily intakes, except MCPP, which is modelled using biomarker concentrations.*

*Abbreviations: BBzP: Benzyl butyl phthalate; BPA: Bisphenol A; DBP: Dibutyl phthalate; DEHP: Di(2-ethylhexyl) phthalate; DEP: Diethyl phthalate; DMP: Dimethyl phthalate; MCPP: Mono(3-carboxypropyl) phthalate; RR: Risk ratios*

|  | Asthma | | | Wheeze | | |
| --- | --- | --- | --- | --- | --- | --- |
|  | Cohorts | N | RR (95%CI) | Cohorts | N | RR (95%CI) |
| MMP | 4 | 1927 | 0.98 (0.92-1.05) | 4 | 1983 | 1.01 (0.98-1.05) |
| MEP | 8 | 2900 | 1.03 (0.98-1.07) | 10 | 3447 | 1.01 (0.98-1.03) |
| ∑MBP | 8 | 2900 | 1.09 (1.01-1.17) | 10 | 3447 | 1.05 (1.01-1.09) |
| MBzP | 8 | 2900 | 1.07 (1.01-1.13) | 10 | 3447 | 1.02 (0.99-1.05) |
| ∑DEHP | 8 | 2900 | 1.03 (0.97-1.10) | 10 | 3447 | 1.02 (0.99-1.05) |
| BPA | 7 | 2163 | 1.01 (0.95-1.08) | 9 | 2710 | 1.01 (0.97-1.04) |
| BPS | 5 | 1808 | 0.96 (0.88-1.05) | 7 | 2231 | 1.00 (0.96-1.04) |
| BPF | 4 | 1570 | 1.04 (0.94-1.16) | 6 | 1993 | 1.01 (0.97-1.06) |
|  | Eczema | | | Rhinitis | | |
|  | Cohorts | N | RR (95%CI) | Cohorts | N | RR (95%CI) |
| MMP | 4 | 1947 | 0.98 (0.95-1.02) | 4 | 1869 | 1.01 (0.96-1.06) |
| MEP | 9 | 3111 | 1.02 (1.00-1.05) | 8 | 2562 | 1.01 (0.98-1.03) |
| ∑MBP | 9 | 3111 | 1.01 (0.98-1.05) | 8 | 2562 | 1.02 (0.98-1.06) |
| MBzP | 9 | 3111 | 1.00 (0.97-1.03) | 8 | 2562 | 1.01 (0.98-1.05) |
| ∑DEHP | 9 | 3111 | 0.97 (0.94-1.01) | 8 | 2562 | 1.04 (1.00-1.07) |
| BPA | 8 | 2409 | 0.99 (0.95-1.03) | 7 | 1860 | 1.01 (0.96-1.06) |
| BPS | 6 | 1945 | 0.95 (0.89-1.00) | 5 | 1396 | 0.98 (0.91-1.04) |
| BPF | 5 | 1707 | 1.03 (0.97-1.09) | 4 | 1158 | 1.06 (1.00-1.13) |

# Table S12. Sensitivity analysis showing adjusted risk ratios of allergic conditions associated with a twofold increase in prenatal urinary biomarkers.

*Models obtained with generalised estimating equations. Models adjusted for cohort, maternal age, ethnicity, parental education, marital status, family history of asthma, sex, prenatal tobacco smoke exposure, and season of birth.*

*Abbreviations: MBzP: Mono Benzyl phthalate; BPA: Bisphenol A; BPF: Bisphenol F; BPS: Bisphenol S; MEP: mono-ethyl phthalate; MMP: mono-methyl phthalate; RR: Risk Ratios; ∑DEHP: molar sum of Di(2-ethylhexyl) phthalate metabolites; ∑MBP: molar sum of mono-iso-butyl phthalate and mono-n-butyl phthalate.*

# Table S13. Sensitivity analysis showing adjusted risk ratios of allergic conditions associated with a twofold increase in postnatal urinary biomarkers.

|  | Asthma | | | Wheeze | | |
| --- | --- | --- | --- | --- | --- | --- |
|  | Cohorts | N | RR (95%CI) | Cohorts | N | RR (95%CI) |
| MEP | 2 | 1175 | 0.98 (0.93-1.03) | 2 | 1719 | 1.00 (0.96-1.04) |
| ∑MBP | 2 | 1098 | 1.11 (0.99-1.25) | 2 | 1589 | 1.04 (0.97-1.11) |
| MBzP | 2 | 1184 | 0.98 (0.93-1.03) | 2 | 1720 | 1.05 (1.01-1.09) |
| ∑DEHP | 2 | 1179 | 1.01 (0.92-1.10) | 2 | 1718 | 1.08 (1.03-1.13) |
| BPA | 1 | 235 | 0.89 (0.78-1.01) | 1 | 303 | 0.98 (0.91-1.06) |
|  | Eczema | | | Rhinitis | | |
|  | Cohorts | N | RR (95%CI) | Cohorts | N | RR (95%CI) |
| MEP | 2 | 1707 | 0.99 (0.97-1.02) | 2 | 1273 | 1.00 (0.96-1.05) |
| ∑MBP | 2 | 1576 | 1.01 (0.97-1.06) | 2 | 1149 | 1.04 (0.94-1.16) |
| MBzP | 2 | 1707 | 0.97 (0.95-1.00) | 2 | 1280 | 1.00 (0.96-1.04) |
| ∑DEHP | 2 | 1705 | 0.94 (0.91-0.97) | 2 | 1276 | 0.99 (0.95-1.05) |
| BPA | 1 | 303 | 1.00 (0.95-1.05) | 1 | 303 | 1.03 (0.98-1.09) |

*Models obtained with generalised estimating equations. Models adjusted for the cohort, maternal age, ethnicity, parental education, marital status, family history of asthma, sex, prenatal tobacco smoke exposure, season of birth, breastfeeding duration, age at outcome assessment, postnatal smoke exposure and gestational age.*

*Abbreviations: MBzP: Mono Benzyl phthalate; BPA: Bisphenol A; MEP: mono-ethyl phthalate; RR: Risk Ratios; ∑DEHP: molar sum of Di(2-ethylhexyl) phthalate metabolites; ∑MBP: molar sum of mono-iso-butyl phthalate and mono-n-butyl phthalate.*

# Table S14. Sensitivity analysis showing adjusted risk ratios and 95% confidence intervals for childhood allergic conditions associated with a twofold increase in DEHP exposure, comparing the main and harmonised DEHP variables.

|  | Asthma | Wheeze | Eczema | Rhinitis |
| --- | --- | --- | --- | --- |
| Prenatal analysis |  |  |  |  |
| DEHP | 1.00 (0.94-1.07) | 1.01 (0.97-1.04) | 0.97 (0.93-1.00) | 1.03 (0.99-1.07) |
| DEHP-harmonised | 1.02 (0.96-1.08) | 1.01 (0.98-1.04) | 0.97 (0.93-1.00) | 1.03 (1.00-1.07) |
| Postnatal analysis |  |  |  |  |
| DEHP | 1.01 (0.95-1.09) | 1.06 (1.01-1.11) | 0.95 (0.91-0.98) | 0.99 (0.94-1.05) |
| DEHP-harmonised | 1.02 (0.96-1.09) | 1.07 (1.02-1.12) | 0.94 (0.91-0.97) | 0.99 (0.94-1.03) |

*The DEHP variable corresponds to the exposure measure used in the main analysis. The harmonised DEHP variable in the prenatal analysis was calculated as estimated daily intake excluding MEHP, while the postnatal harmonised DEHP variable excluded both MEHP and MECPP. Models obtained with generalised estimating equations. Prenatal models were adjusted for cohort, maternal age, ethnicity, parental education, marital status, family history of asthma, child’s sex, prenatal tobacco smoke exposure, and season of birth. Postnatal models were further adjusted for breastfeeding duration, child’s age at outcome assessment, postnatal tobacco smoke exposure, and gestational age.*

#
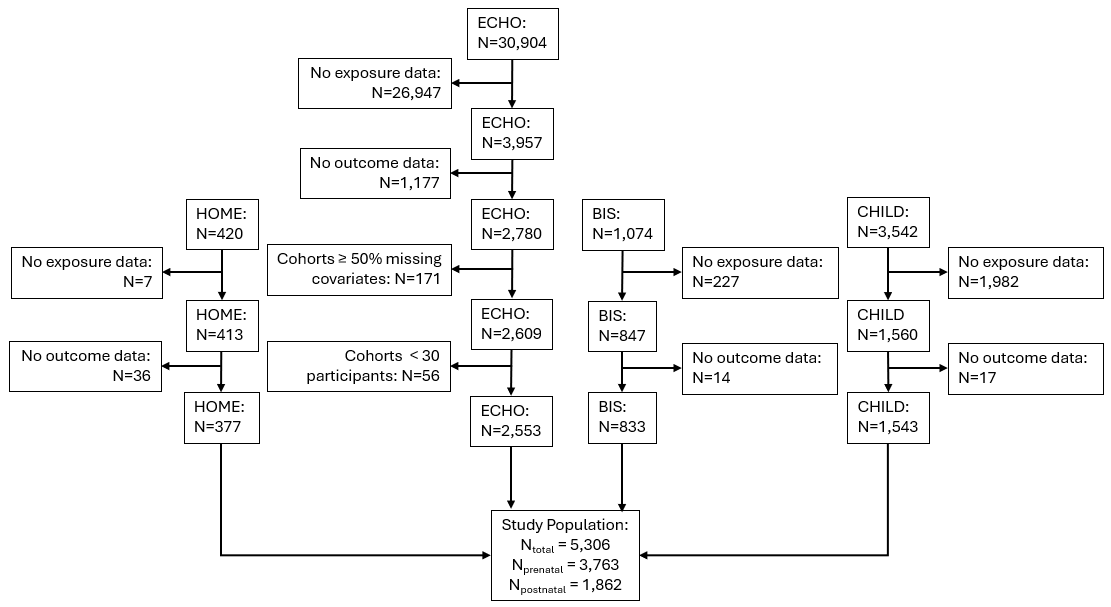
Figure S1. Study population inclusion flow-chart

*3,444 children had exposure measured during pregnancy only, 1,543 during childhood only, and 319 during both periods*

*Abbreviations: BIS: Barwon Infant Study; CHILD: Canadian Healthy Infant Longitudinal Development; ECHO: Environmental Influences on Child Health Outcomes; HOME: Health Outcomes and Measures of the Environment*


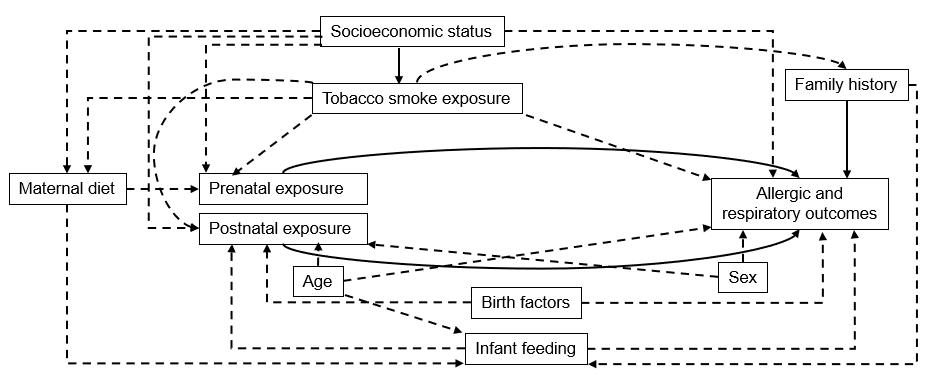


# Figure S2. Directed acyclic graph representing the association between prenatal and postnatal exposure to phthalates, bisphenols, and allergic diseases

Solid lines represent causal pathways, and non-solid lines represent non-causal pathways. Socioeconomic status includes maternal age, ethnicity, marital status and education. Birth factors include gestational age.

#
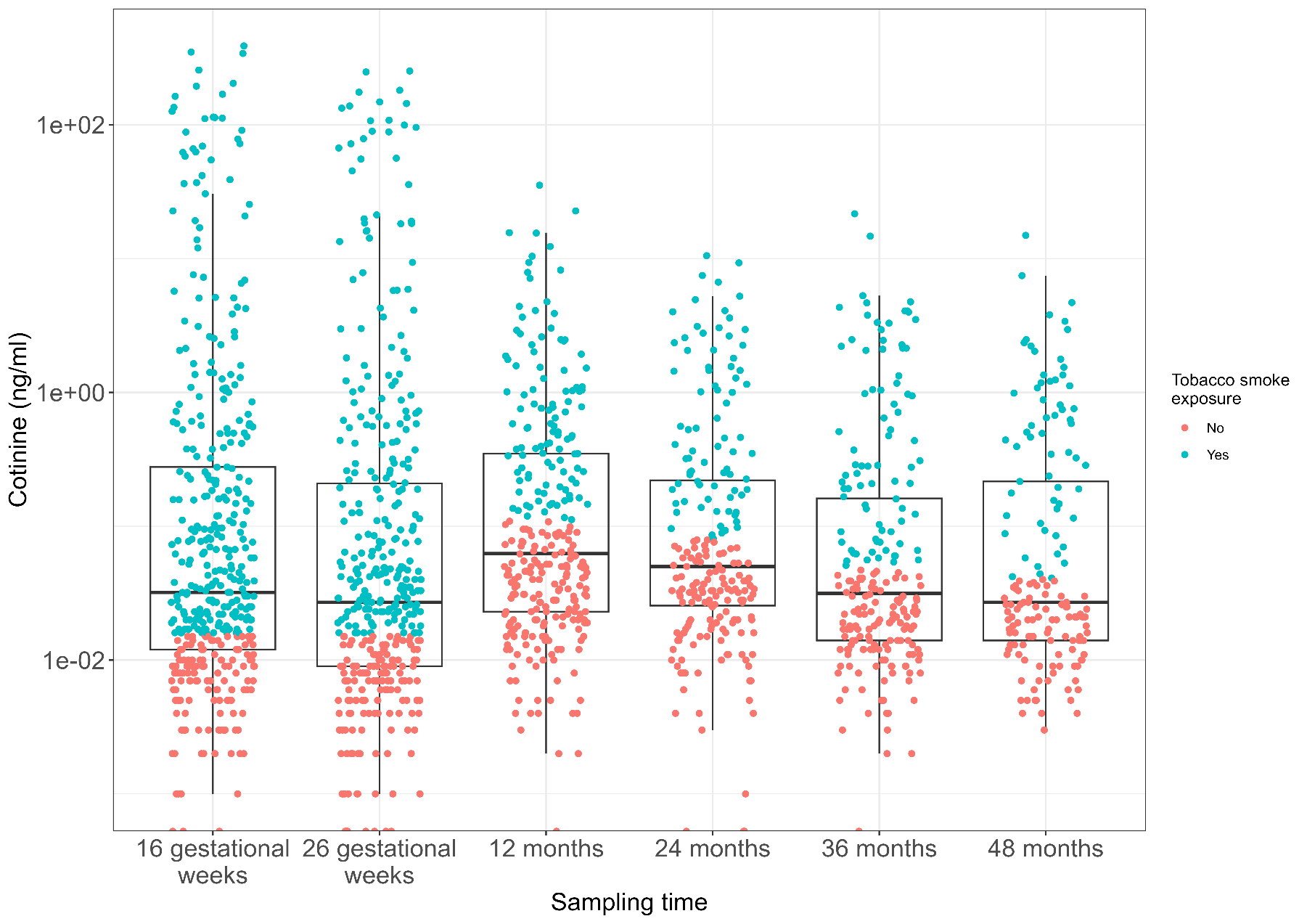
Figure S3. Distribution of serum cotinine concentrations across time points in HOME.

Abbreviations: *HOME: Health Outcomes and Measures of the Environment*


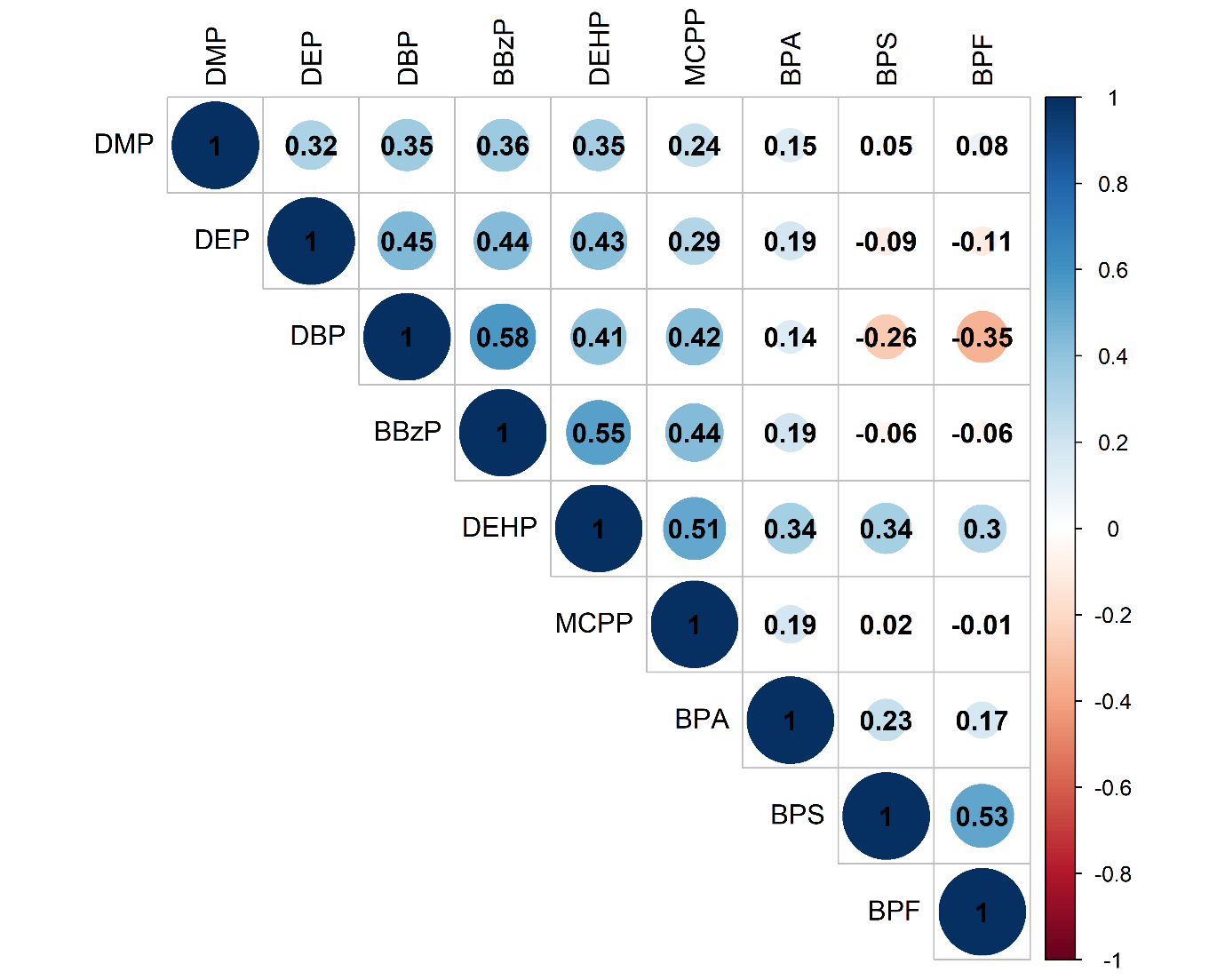


# Figure S4. Spearman correlation plot of prenatal phthalates and bisphenols

Abbreviations: BBzP: Benzyl butyl phthalate; BPA: Bisphenol A; BPF: Bisphenol F; BPS: Bisphenol S; DBP: Dibutyl phthalate; DEHP: Di(2-ethylhexyl) phthalate; DEP: Diethyl phthalate; DMP: Dimethyl phthalate; MCPP: Mono-(3-carboxypropyl) phthalate


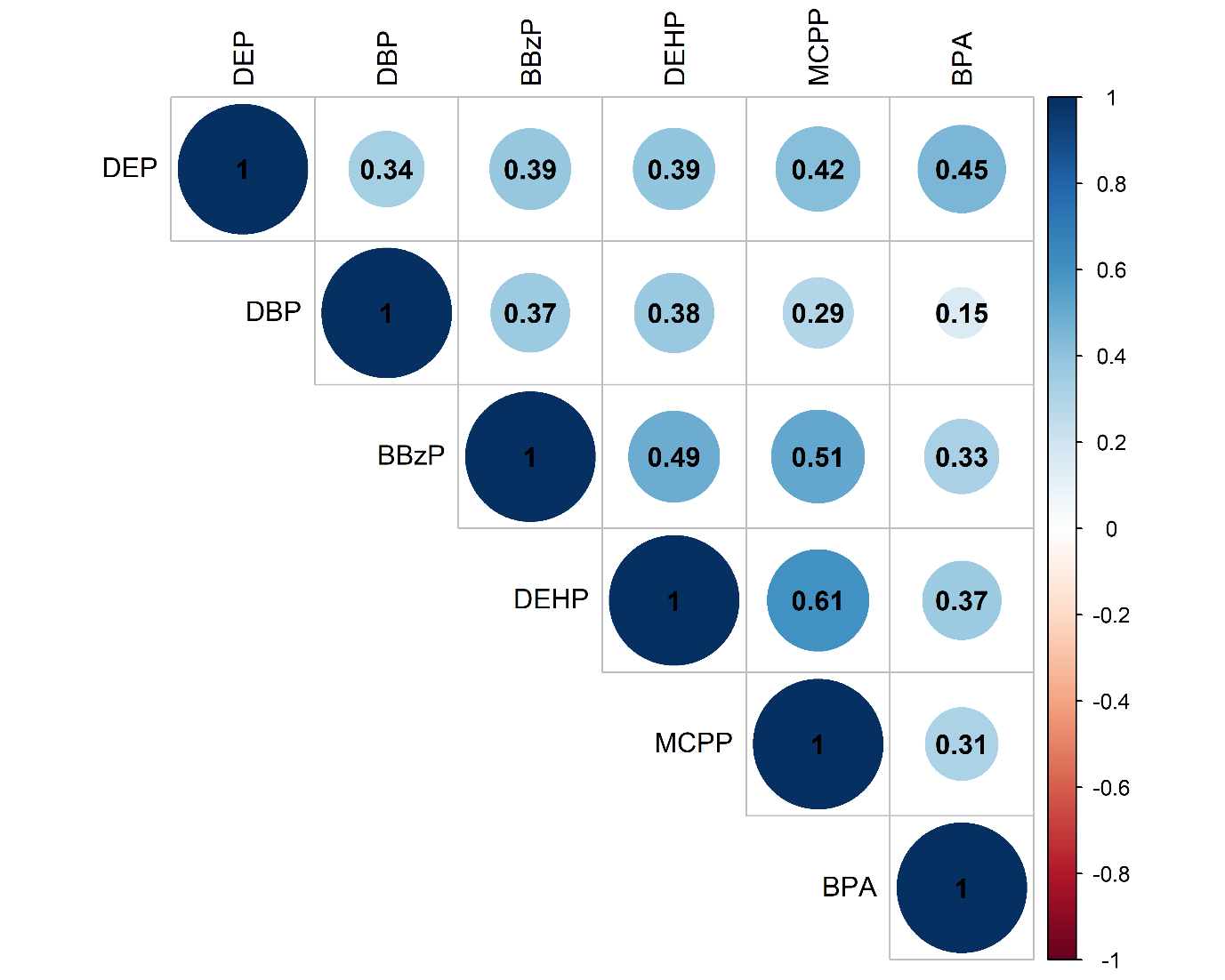


# Figure S5. Spearman correlation plot of postnatal phthalates and bisphenols

Abbreviations: BBzP: Benzyl butyl phthalate; BPA: Bisphenol A; DBP: Dibutyl phthalate; DEHP: Di(2-ethylhexyl) phthalate; DEP: Diethyl phthalate; DMP: Dimethyl phthalate; MCPP: Mono-(3-carboxypropyl) phthalate

#
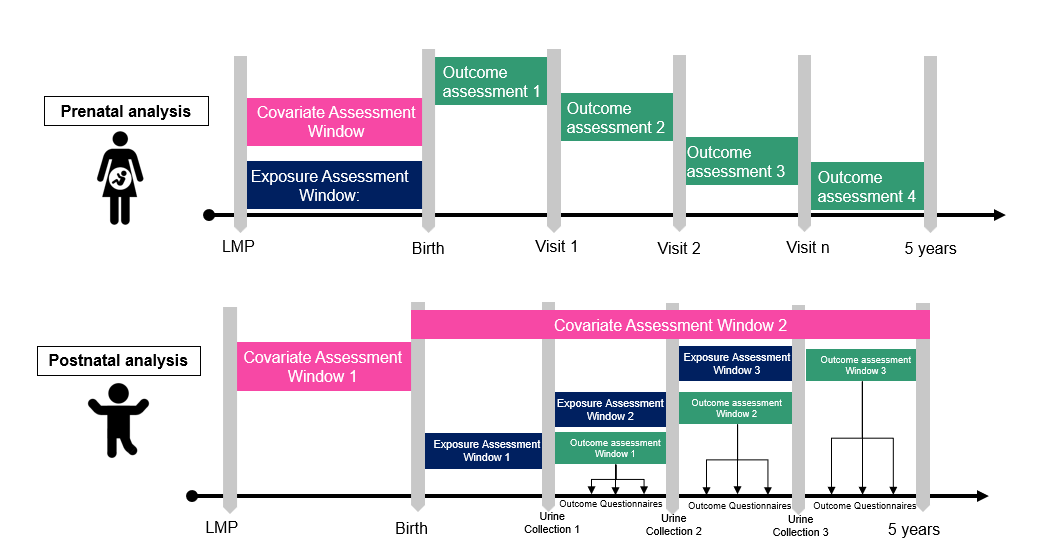
Figure S6. Study-design diagram

*LMP: Last menstrual period*

#
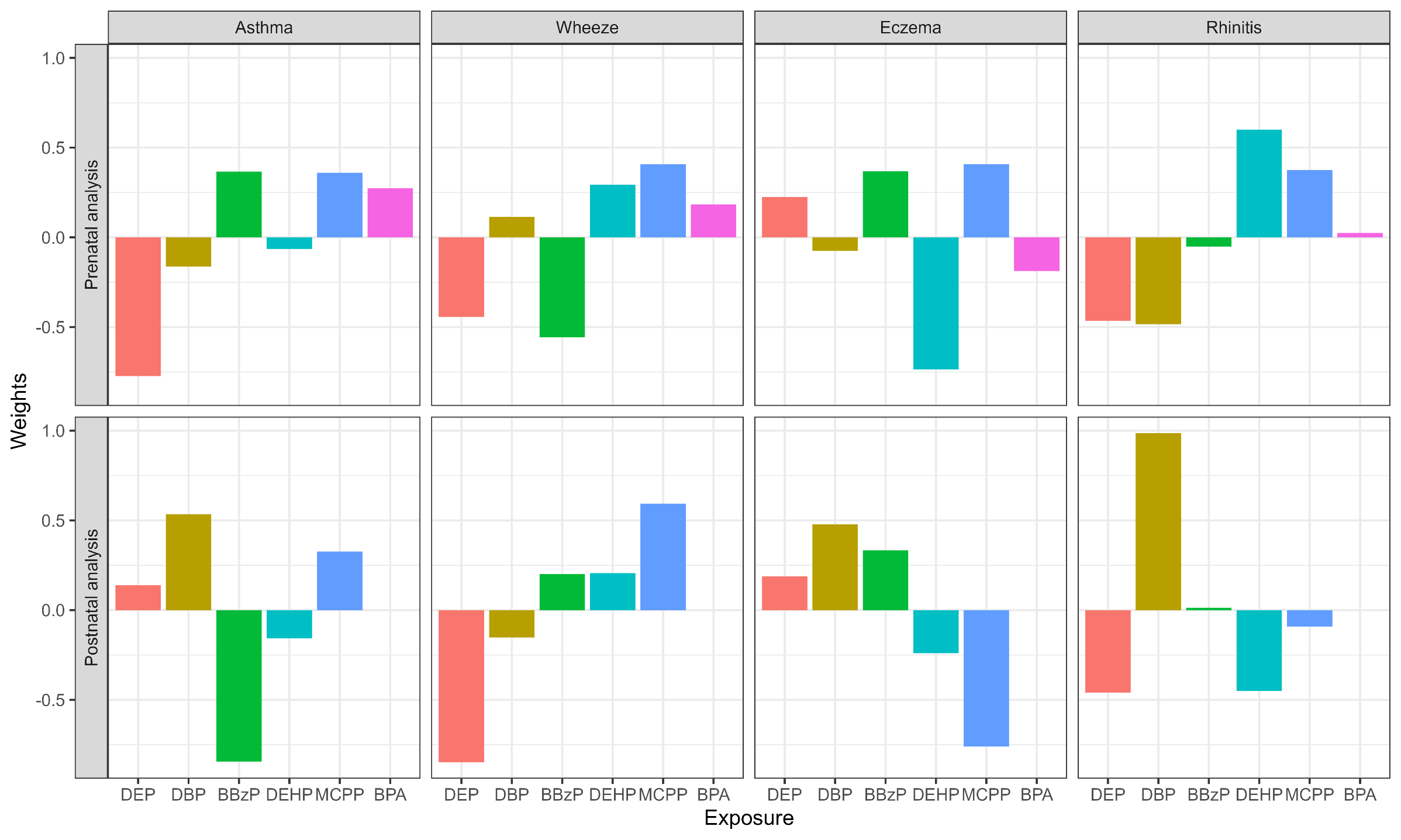
Figure S7. Single compounds relative weights in quantile G computation models

*Abbreviations:* *BBzP: Butyl Benzyl Phthalate; BPA: Bisphenol A; DBP: Dibutyl Phthalate; DEHP: Di(2-ethylhexyl) Phthalate; DEP: Diethyl Phthalate; MCPP: Mono(3-carboxypropyl) Phthalate.* *All exposures are modelled as estimated daily intakes, except MCPP, which is modelled using biomarker concentrations.*

#
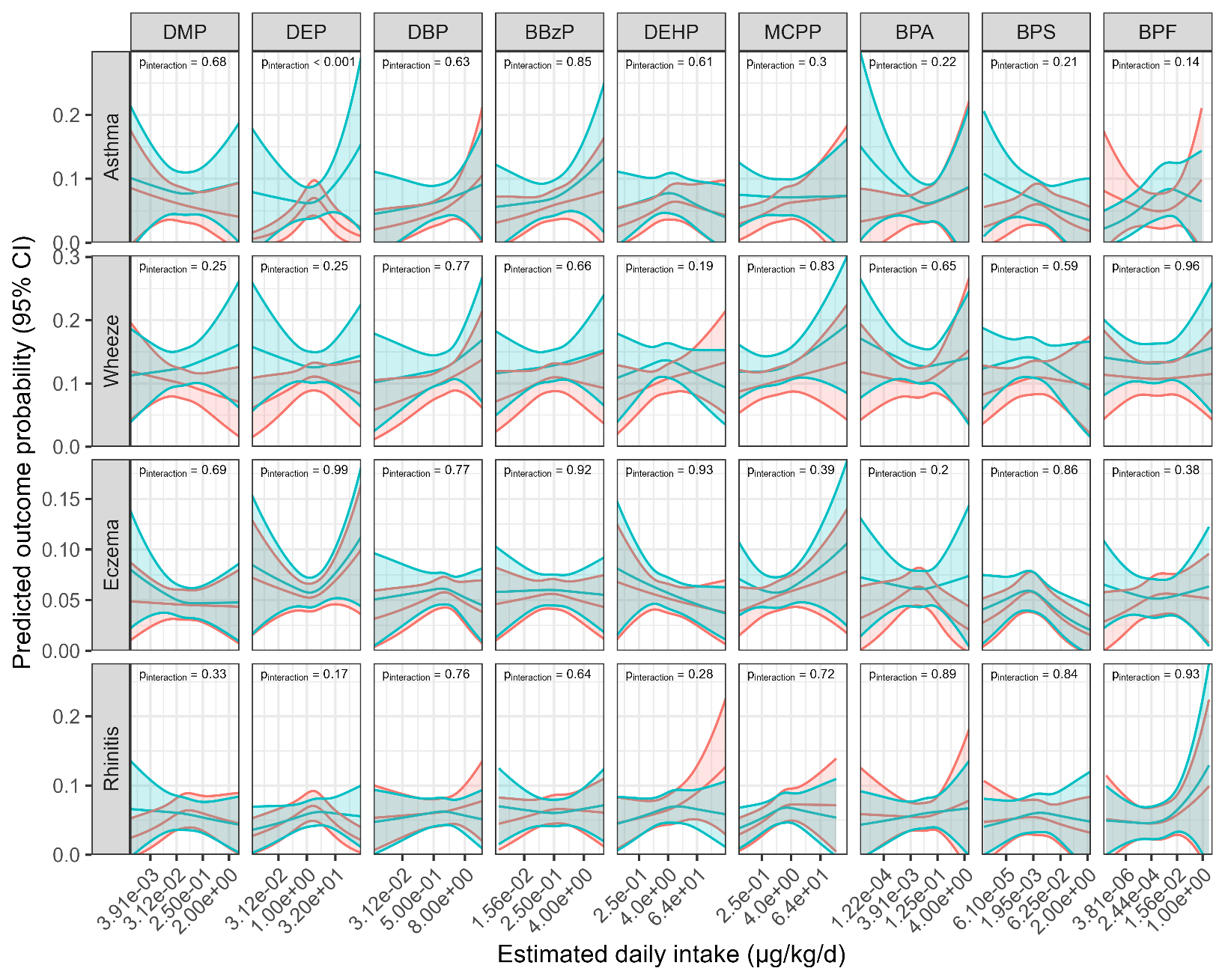
Figure S8. Risk of childhood allergic conditions associated with prenatal exposure to phthalates and bisphenols, stratified by sex.

*Models obtained with generalised estimating equations.* Dose-response relationships are displayed with continuous covariates held at their mean values and categorical covariates set to their reference category. Models adjusted for the cohort, maternal age, ethnicity, parental education, lone parenthood, family history of asthma, prenatal tobacco smoke exposure, and season of birth. All exposures are modelled as estimated daily intakes, except MCPP, which is modelled using biomarker concentrations. Abbreviations: BBzP: Benzyl butyl phthalate; BPA: Bisphenol A; BPF: Bisphenol F; BPS: Bisphenol S; DBP: Dibutyl phthalate; DEHP: Di(2-ethylhexyl) phthalate; DEP: Diethyl phthalate; DMP: Dimethyl phthalate; MCPP: Mono-(3-carboxypropyl) phthalate

#
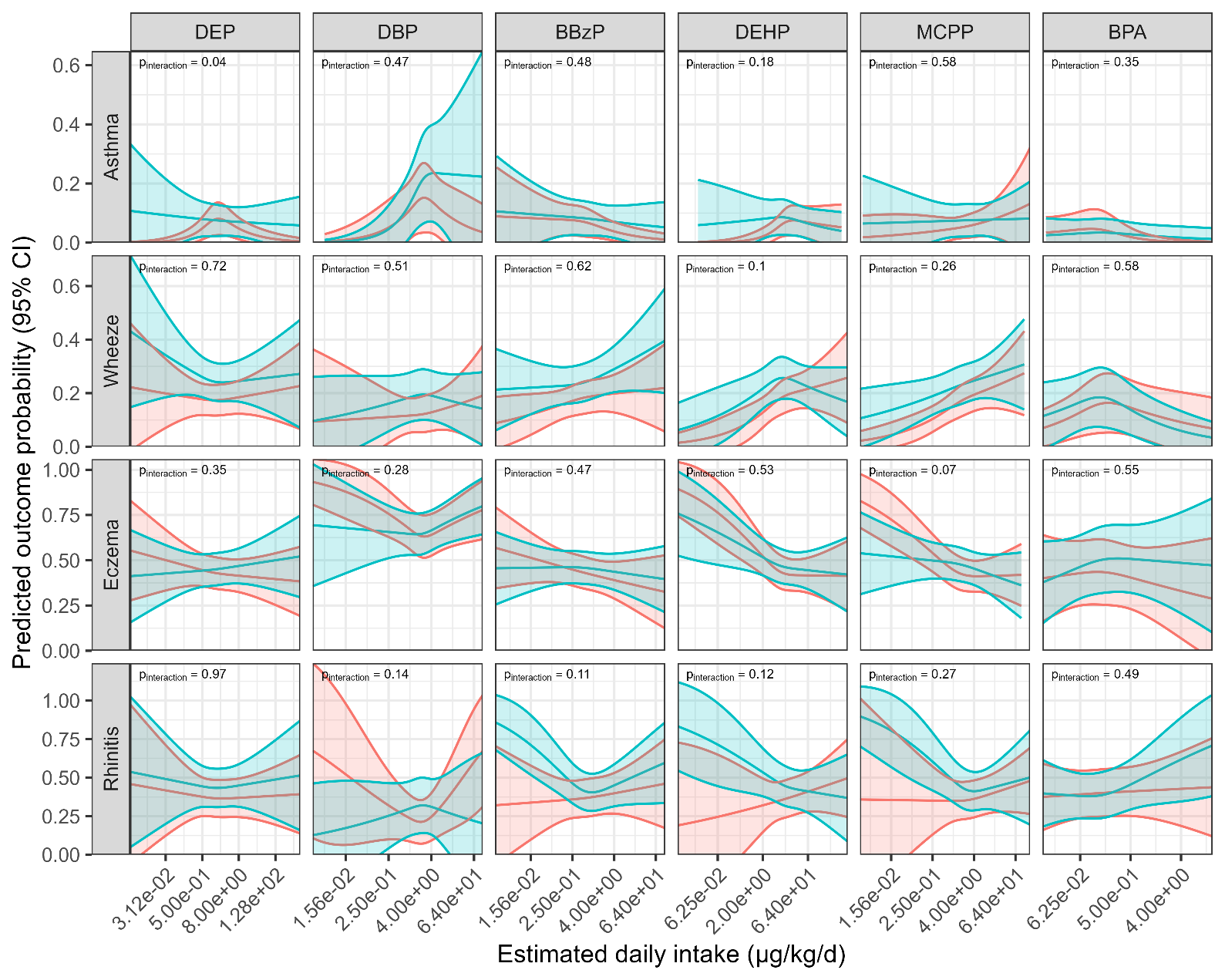
Figure S9. Risk of childhood allergic conditions associated with postnatal exposure to phthalates and bisphenols, stratified by sex

*Models obtained with generalised estimating equations. Dose-response relationships are displayed with continuous covariates held at their mean values and categorical covariates set to their reference category. Models adjusted for the cohort, maternal age, ethnicity, parental education, lone parenthood, family history of asthma, prenatal tobacco smoke exposure, season of birth, breastfeeding duration, age at outcome assessment, postnatal smoke exposure and gestational age. All exposures are modelled as estimated daily intakes, except MCPP, which is modelled using biomarker concentrations. Abbreviations: BBzP: Benzyl butyl phthalate; BPA: Bisphenol A; DBP: Dibutyl phthalate; DEHP: Di(2-ethylhexyl) phthalate; DEP: Diethyl phthalate; MCPP: Mono-(3-carboxypropyl) phthalate*

*
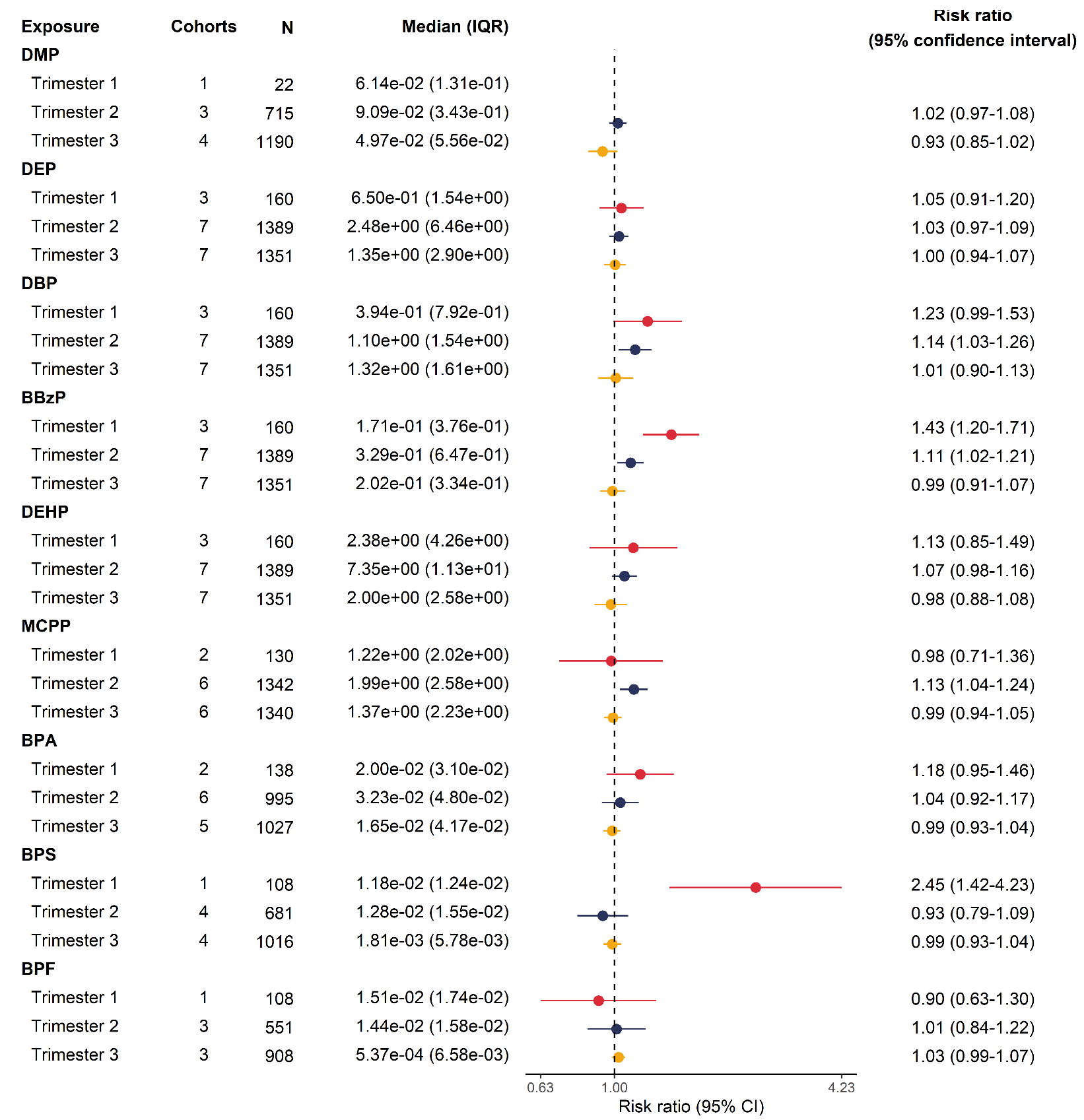
*

# Figure S10. Adjusted risk ratios for asthma associated with a two-fold increase in prenatal phthalates and bisphenols, stratified by trimester of exposure

*Model obtained risk generalised estimating equations.* Median and IQR are shown in µg/kg/day, except for MCPP (µg/L). *Models adjusted for cohort, maternal age, ethnicity, parental education, marital status, family history of asthma, sex, prenatal tobacco smoke exposure, and season of birth. All exposures are modelled as estimated daily intakes, except MCPP, which is modelled using biomarker concentrations.*

*Abbreviations: BBzP: Benzyl butyl phthalate; BPA: Bisphenol A; BPF: Bisphenol F; BPS: Bisphenol S; DBP: Dibutyl phthalate; DEHP: Di(2-ethylhexyl) phthalate; DEP: Diethyl phthalate; DMP: Dimethyl phthalate; IQR: Interquartile range; MCPP: Mono(3-carboxypropyl) phthalate.*

#
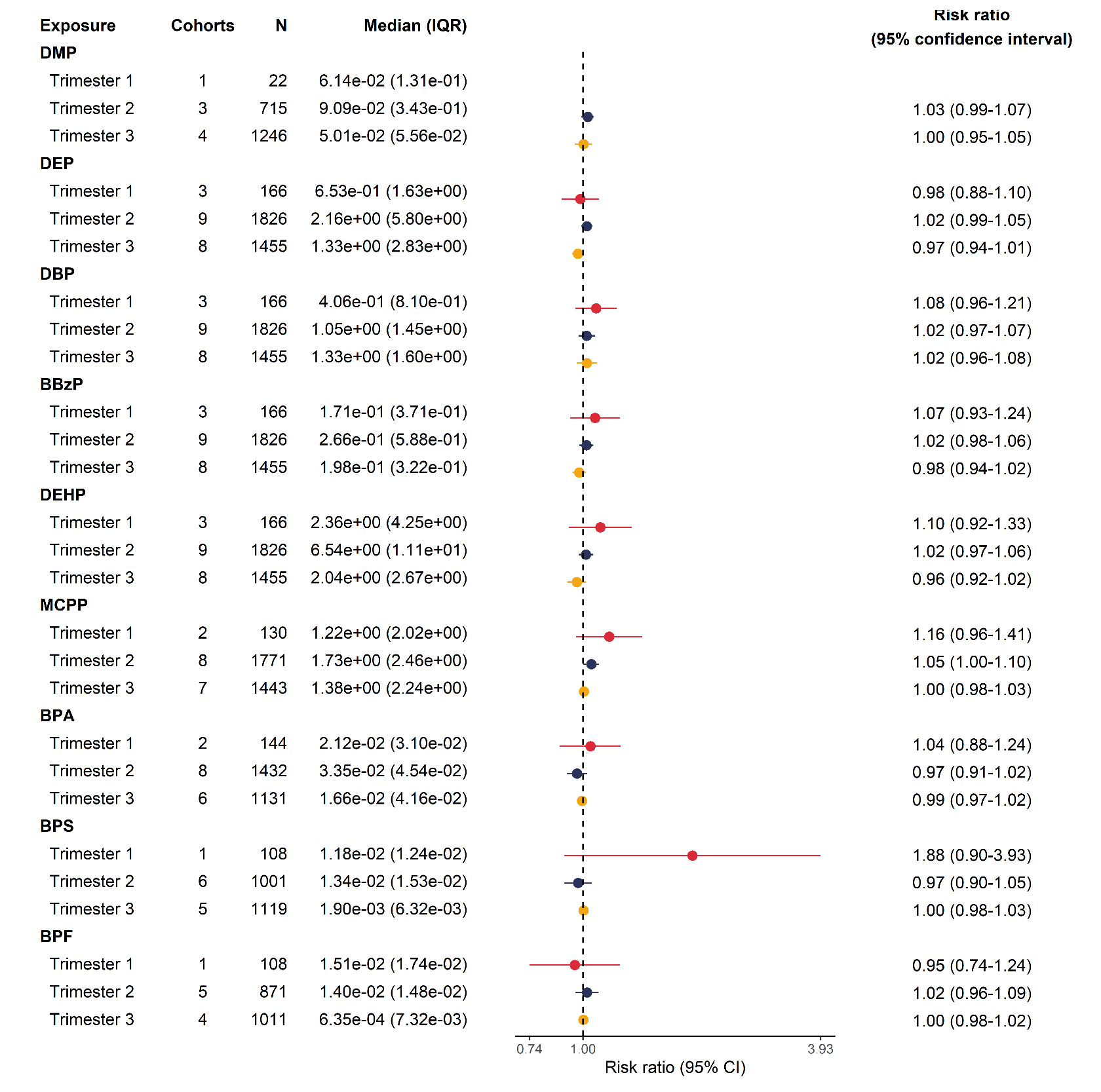
Figure S11. Adjusted risk ratios for wheeze associated with a two-fold increase in prenatal phthalates and bisphenols, stratified by trimester of exposure

*Model obtained risk generalised estimating equations.* Median and IQR are shown in µg/kg/day, except for MCPP (µg/L). *Models adjusted for cohort, maternal age, ethnicity, parental education, marital status, family history of asthma, sex, prenatal tobacco smoke exposure, and season of birth. All exposures are modelled as estimated daily intakes, except MCPP, which is modelled using biomarker concentrations.*

*Abbreviations: BBzP: Benzyl butyl phthalate; BPA: Bisphenol A; BPF: Bisphenol F; BPS: Bisphenol S; DBP: Dibutyl phthalate; DEHP: Di(2-ethylhexyl) phthalate; DEP: Diethyl phthalate; DMP: Dimethyl phthalate; IQR: Interquartile range; MCPP: Mono(3-carboxypropyl) phthalate.*

#
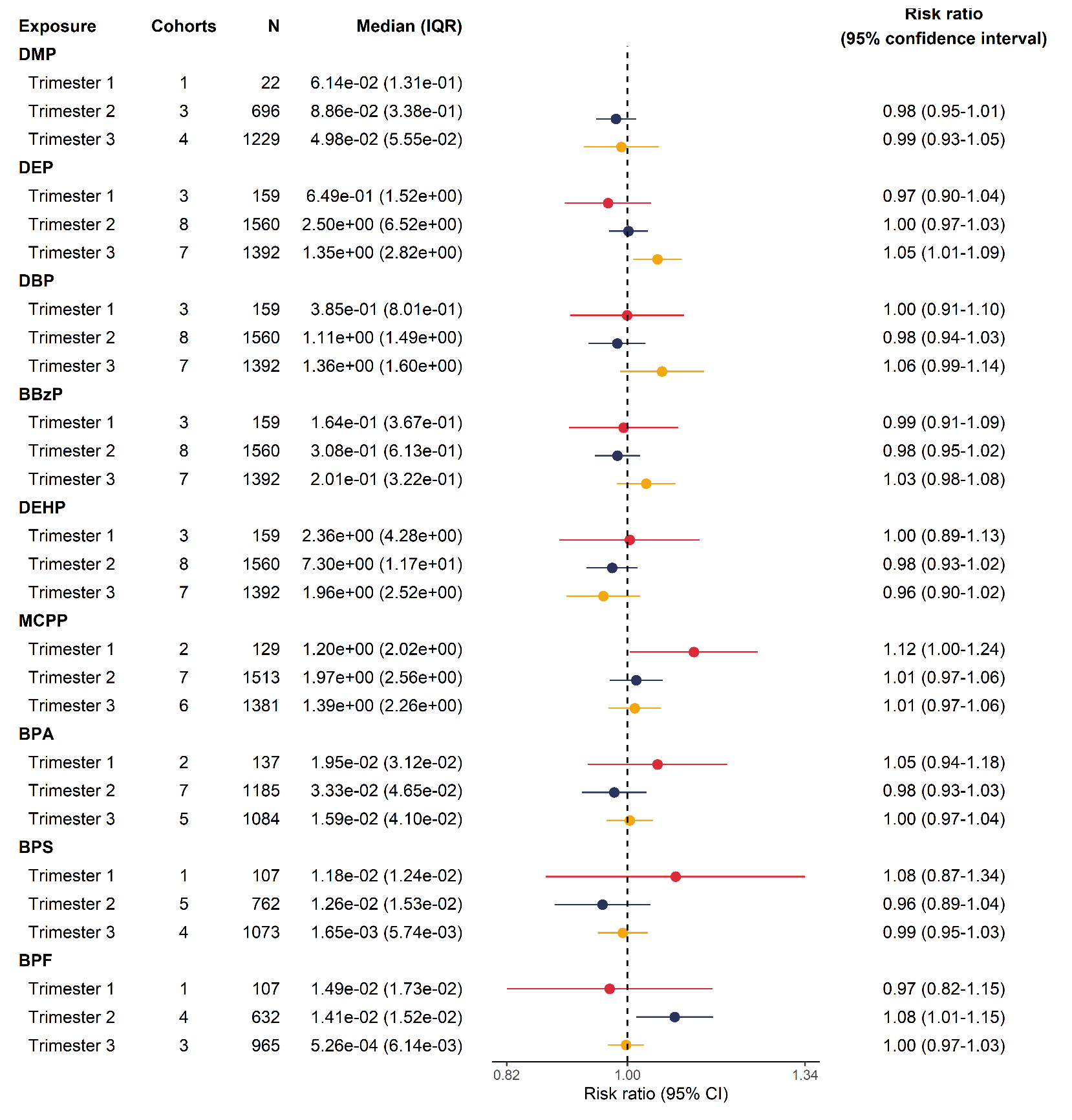
Figure S12. Adjusted risk ratios for eczema associated with a two-fold increase in prenatal phthalates and bisphenols, stratified by trimester of exposure

*Model obtained risk generalised estimating equations.* Median and IQR are shown in µg/kg/day, except for MCPP (µg/L). *Models adjusted for cohort, maternal age, ethnicity, parental education, marital status, family history of asthma, sex, prenatal tobacco smoke exposure, and season of birth. All exposures are modelled as estimated daily intakes, except MCPP, which is modelled using biomarker concentrations.*

*Abbreviations: BBzP: Benzyl butyl phthalate; BPA: Bisphenol A; BPF: Bisphenol F; BPS: Bisphenol S; DBP: Dibutyl phthalate; DEHP: Di(2-ethylhexyl) phthalate; DEP: Diethyl phthalate; DMP: Dimethyl phthalate; IQR: Interquartile range; MCPP: Mono(3-carboxypropyl) phthalate.*

#
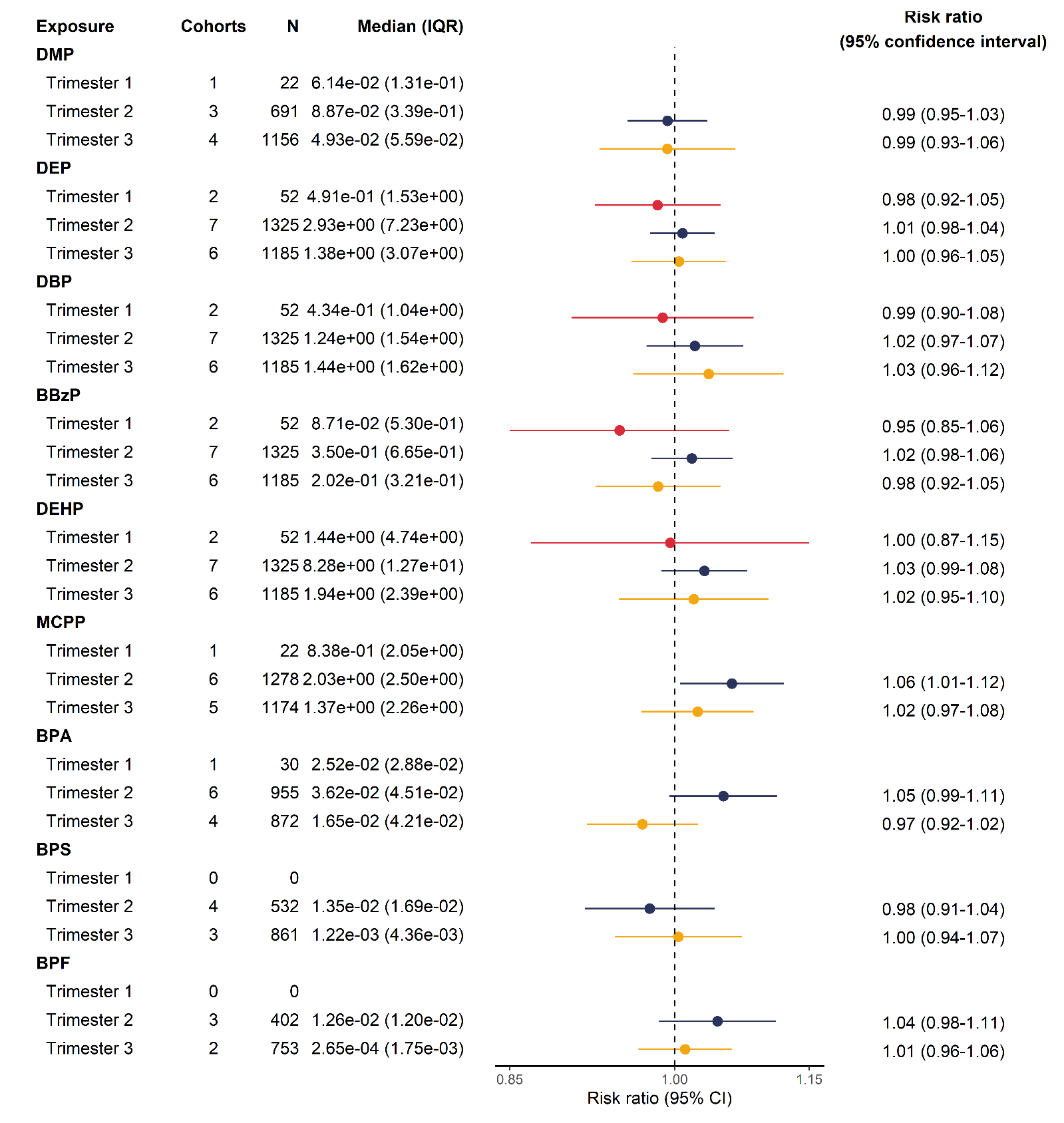
Figure S13. Adjusted risk ratios for rhinitis associated with a two-fold increase in prenatal phthalates and bisphenols, stratified by trimester of exposure

*Model obtained risk generalised estimating equations.* Median and IQR are shown in µg/kg/day, except for MCPP (µg/L). *Models adjusted for cohort, maternal age, ethnicity, parental education, marital status, family history of asthma, sex, prenatal tobacco smoke exposure, and season of birth. All exposures are modelled as estimated daily intakes, except MCPP, which is modelled using biomarker concentrations.*

*Abbreviations: BBzP: Benzyl butyl phthalate; BPA: Bisphenol A; BPF: Bisphenol F; BPS: Bisphenol S; DBP: Dibutyl phthalate; DEHP: Di(2-ethylhexyl) phthalate; DEP: Diethyl phthalate; DMP: Dimethyl phthalate; IQR: Interquartile range; MCPP: Mono(3-carboxypropyl) phthalate.*

#
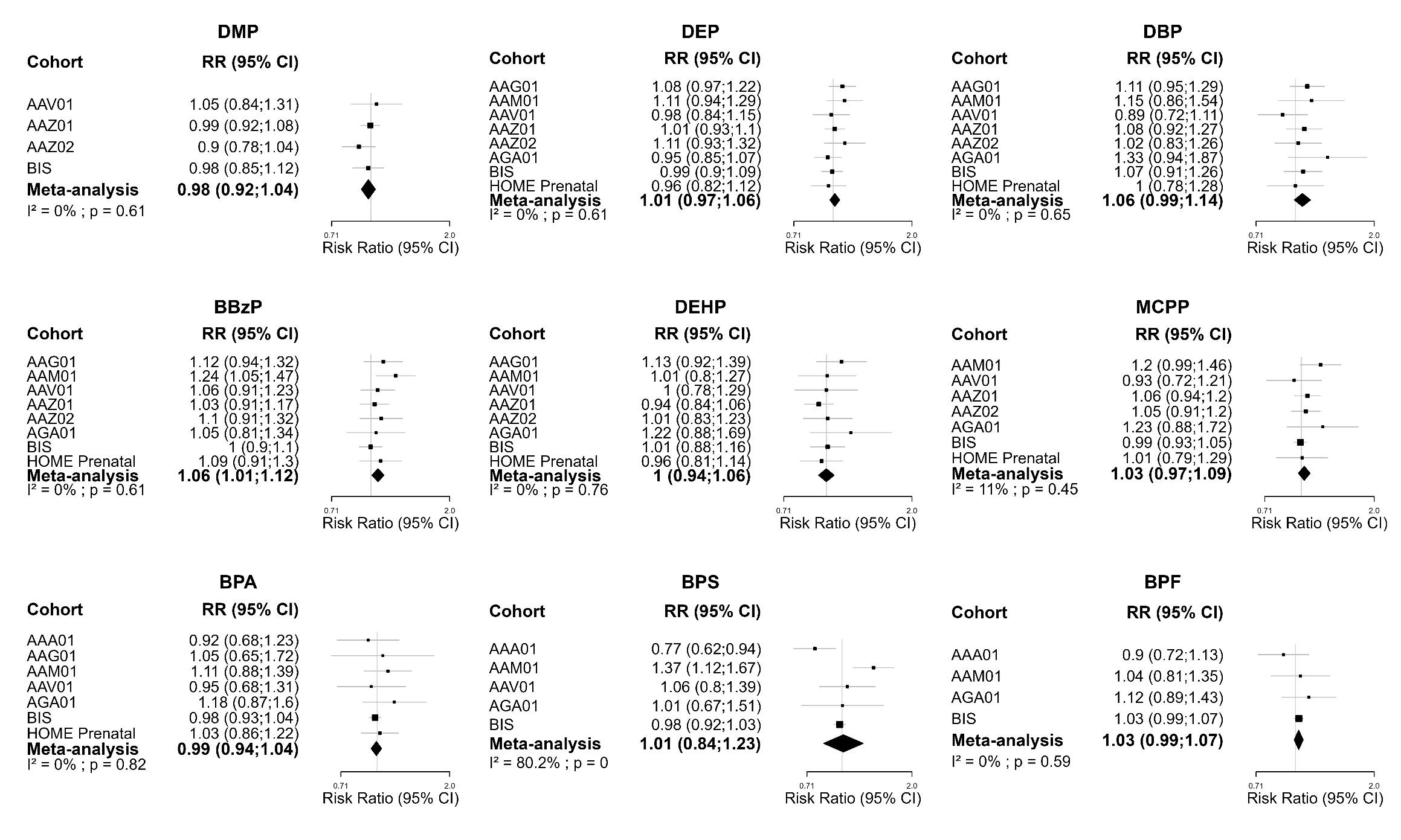
Figure S14. Random-effects Meta-analysis of Cohort-specific Estimates of Prenatal Phthalate and Bisphenol Exposure and Childhood Asthma

*Models adjusted for maternal age, ethnicity, parental education, lone parenthood, family history of asthma, sex, prenatal tobacco smoke exposure, and season of birth. All exposures are modelled as estimated daily intakes, except MCPP, which is modelled using biomarker concentrations.* Abbreviations: BBzP: Benzyl butyl phthalate; BPA: Bisphenol A; BPF: Bisphenol F; BPS: Bisphenol S; DBP: Dibutyl phthalate; DEHP: Di(2-ethylhexyl) phthalate; DEP: Diethyl phthalate; DMP: Dimethyl phthalate; MCPP: Mono-(3-carboxypropyl) phthalate; RR: Risk ratio

#
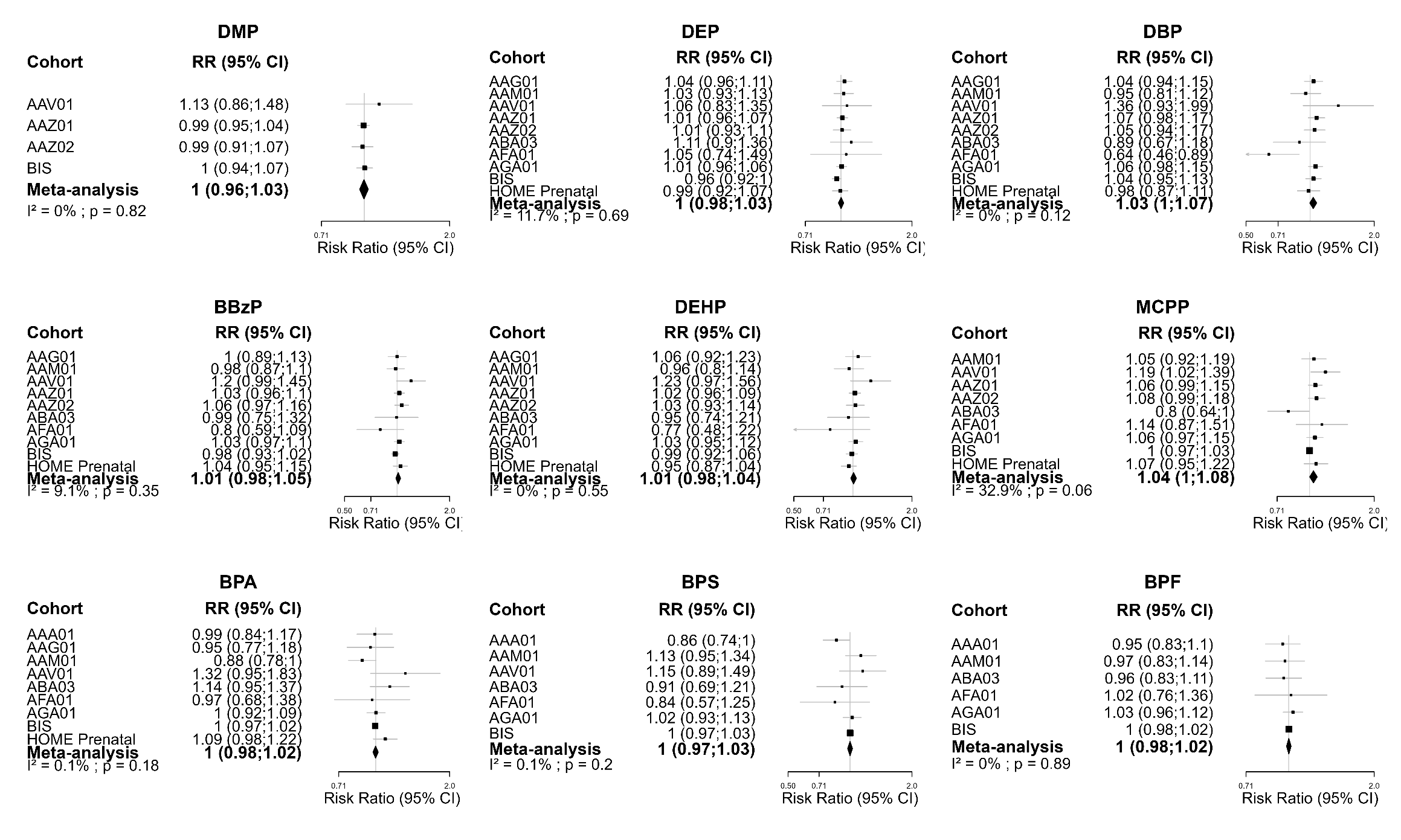
Figure S15. Random-effects Meta-analysis of Cohort-specific Estimates of Prenatal Phthalate and Bisphenol Exposure and Childhood Wheeze

*Models adjusted for maternal age, ethnicity, parental education, lone parenthood, family history of asthma, sex, prenatal tobacco smoke exposure, and season of birth. All exposures are modelled as estimated daily intakes, except MCPP, which is modelled using biomarker concentrations.* Abbreviations: BBzP: Benzyl butyl phthalate; BPA: Bisphenol A; BPF: Bisphenol F; BPS: Bisphenol S; DBP: Dibutyl phthalate; DEHP: Di(2-ethylhexyl) phthalate; DEP: Diethyl phthalate; DMP: Dimethyl phthalate; MCPP: Mono-(3-carboxypropyl) phthalate; RR: Risk ratio

#
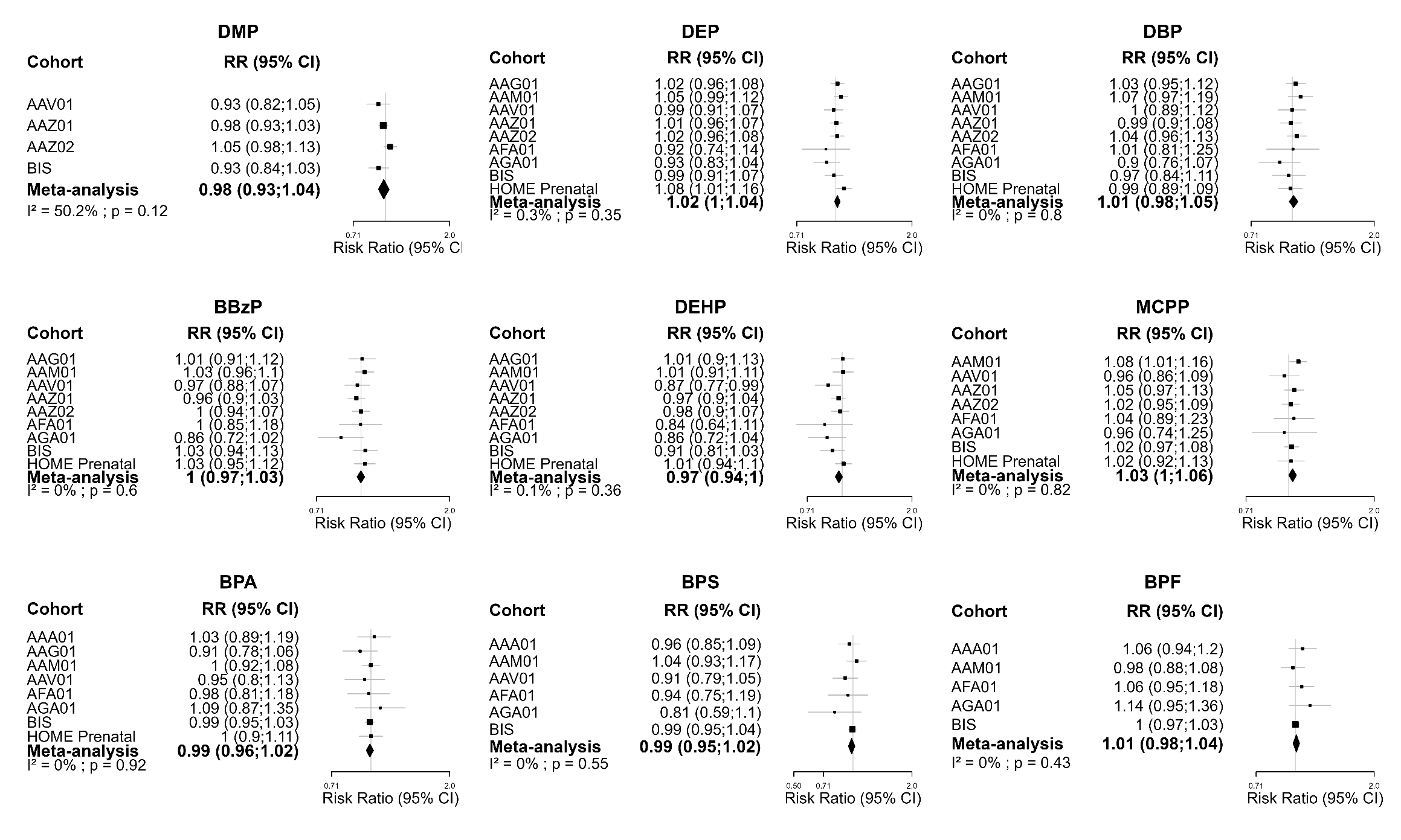
Figure S16. Random-effects Meta-analysis of Cohort-specific Estimates of Prenatal Phthalate and Bisphenol Exposure and Childhood Eczema

*Models adjusted for maternal age, ethnicity, parental education, lone parenthood, family history of asthma, sex, prenatal tobacco smoke exposure, and season of birth. All exposures are modelled as estimated daily intakes, except MCPP, which is modelled using biomarker concentrations.* Abbreviations: BBzP: Benzyl butyl phthalate; BPA: Bisphenol A; BPF: Bisphenol F; BPS: Bisphenol S; DBP: Dibutyl phthalate; DEHP: Di(2-ethylhexyl) phthalate; DEP: Diethyl phthalate; DMP: Dimethyl phthalate; MCPP: Mono-(3-carboxypropyl) phthalate; RR: Risk ratio

#
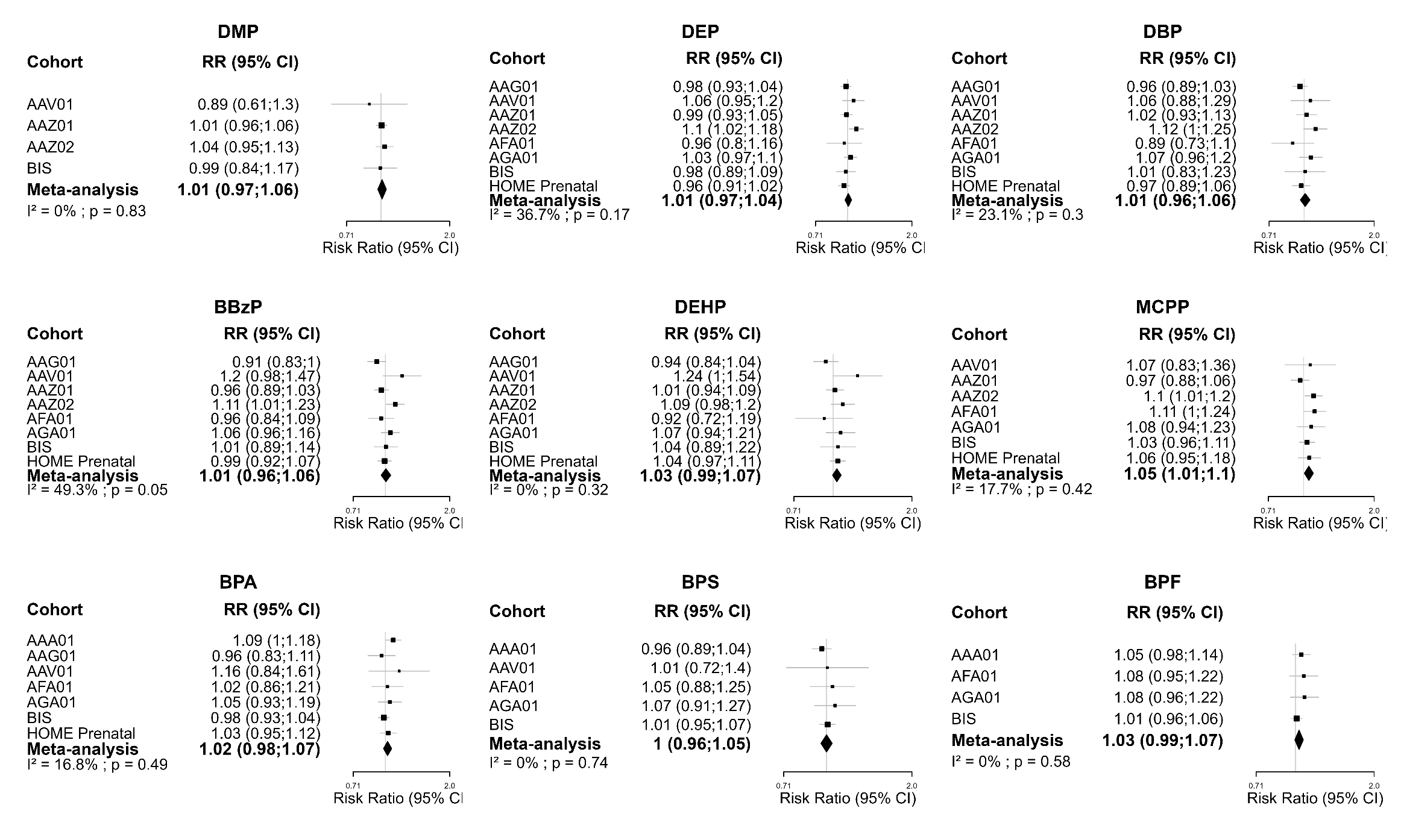
Figure S17. Random-effects Meta-analysis of Cohort-specific Estimates of Prenatal Phthalate and Bisphenol Exposure and Childhood Rhinitis

*Models adjusted for maternal age, ethnicity, parental education, lone parenthood, family history of asthma, sex, prenatal tobacco smoke exposure, and season of birth. All exposures are modelled as estimated daily intakes, except MCPP, which is modelled using biomarker concentrations.* Abbreviations: BBzP: Benzyl butyl phthalate; BPA: Bisphenol A; BPF: Bisphenol F; BPS: Bisphenol S; DBP: Dibutyl phthalate; DEHP: Di(2-ethylhexyl) phthalate; DEP: Diethyl phthalate; DMP: Dimethyl phthalate; MCPP: Mono-(3-carboxypropyl) phthalate; RR: Risk ratio

#
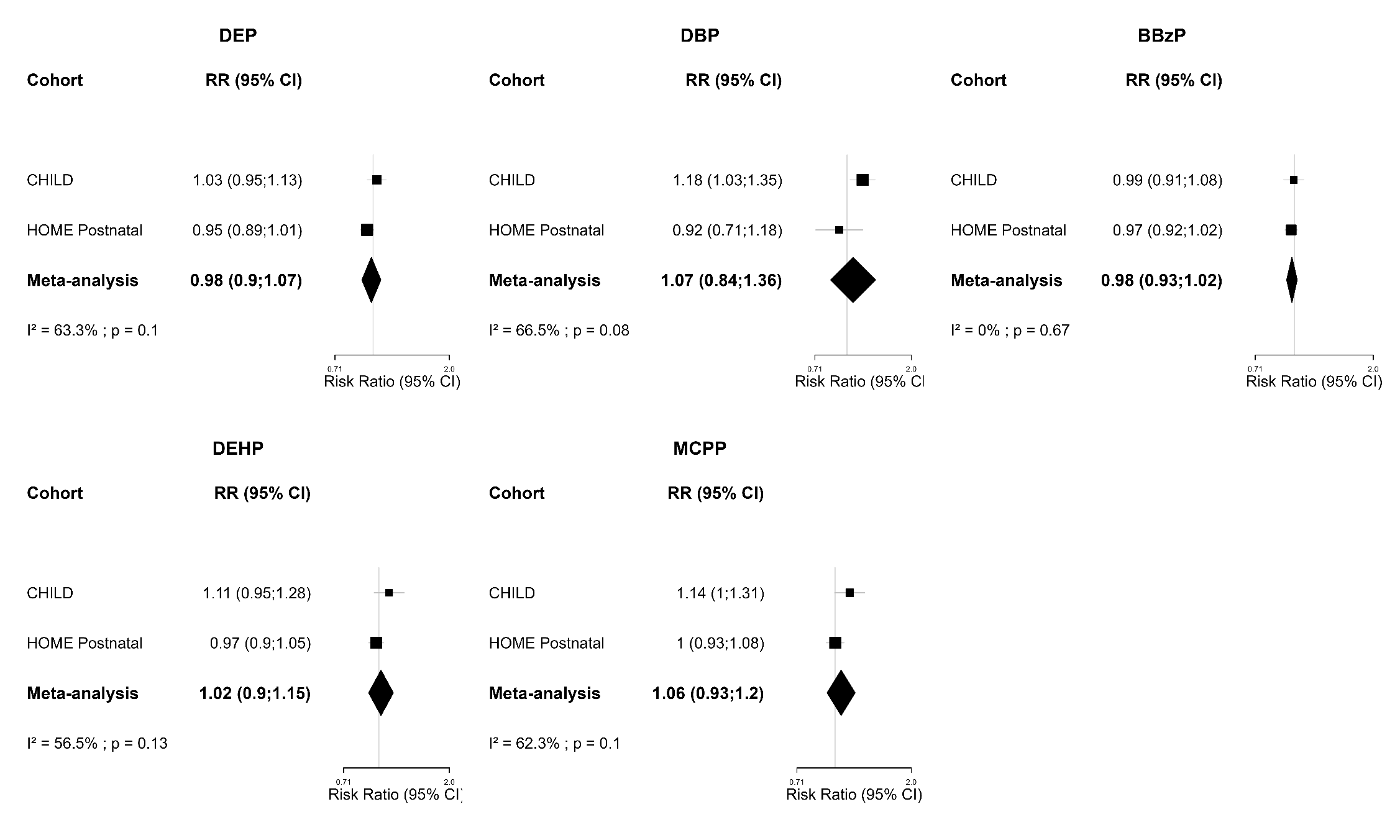
Figure S18. Random-effects Meta-analysis of Cohort-specific Estimates of postnatal Phthalate and Bisphenol Exposure and Childhood Asthma

*Models adjusted for maternal age, ethnicity, parental education, lone parenthood, family history of asthma, sex, prenatal tobacco smoke exposure, and season of birth. All exposures are modelled as estimated daily intakes, except MCPP, which is modelled using biomarker concentrations.* Abbreviations: BBzP: Benzyl butyl phthalate; DBP: Dibutyl phthalate; DEHP: Di(2-ethylhexyl) phthalate; DEP: Diethyl phthalate; DMP: Dimethyl phthalate; MCPP: Mono-(3-carboxypropyl) phthalate; RR: Risk ratio

#
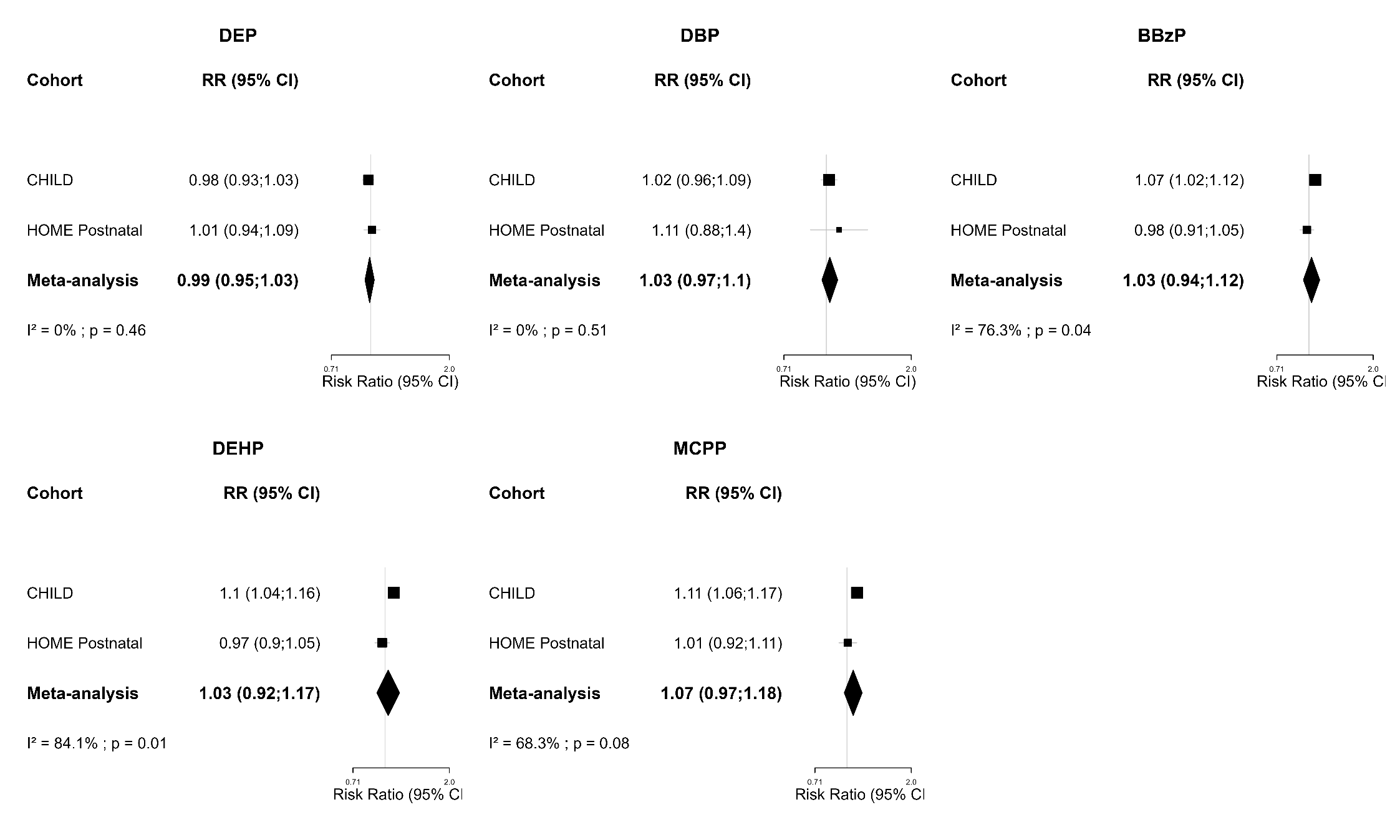
Figure S19. Random-effects Meta-analysis of Cohort-specific Estimates of postnatal Phthalate and Bisphenol Exposure and Childhood Wheeze

*Models adjusted for maternal age, ethnicity, parental education, lone parenthood, family history of asthma, sex, prenatal tobacco smoke exposure, and season of birth. All exposures are modelled as estimated daily intakes, except MCPP, which is modelled using biomarker concentrations.* Abbreviations: BBzP: Benzyl butyl phthalate; DBP: Dibutyl phthalate; DEHP: Di(2-ethylhexyl) phthalate; DEP: Diethyl phthalate; MCPP: Mono-(3-carboxypropyl) phthalate; RR: Risk ratio

#
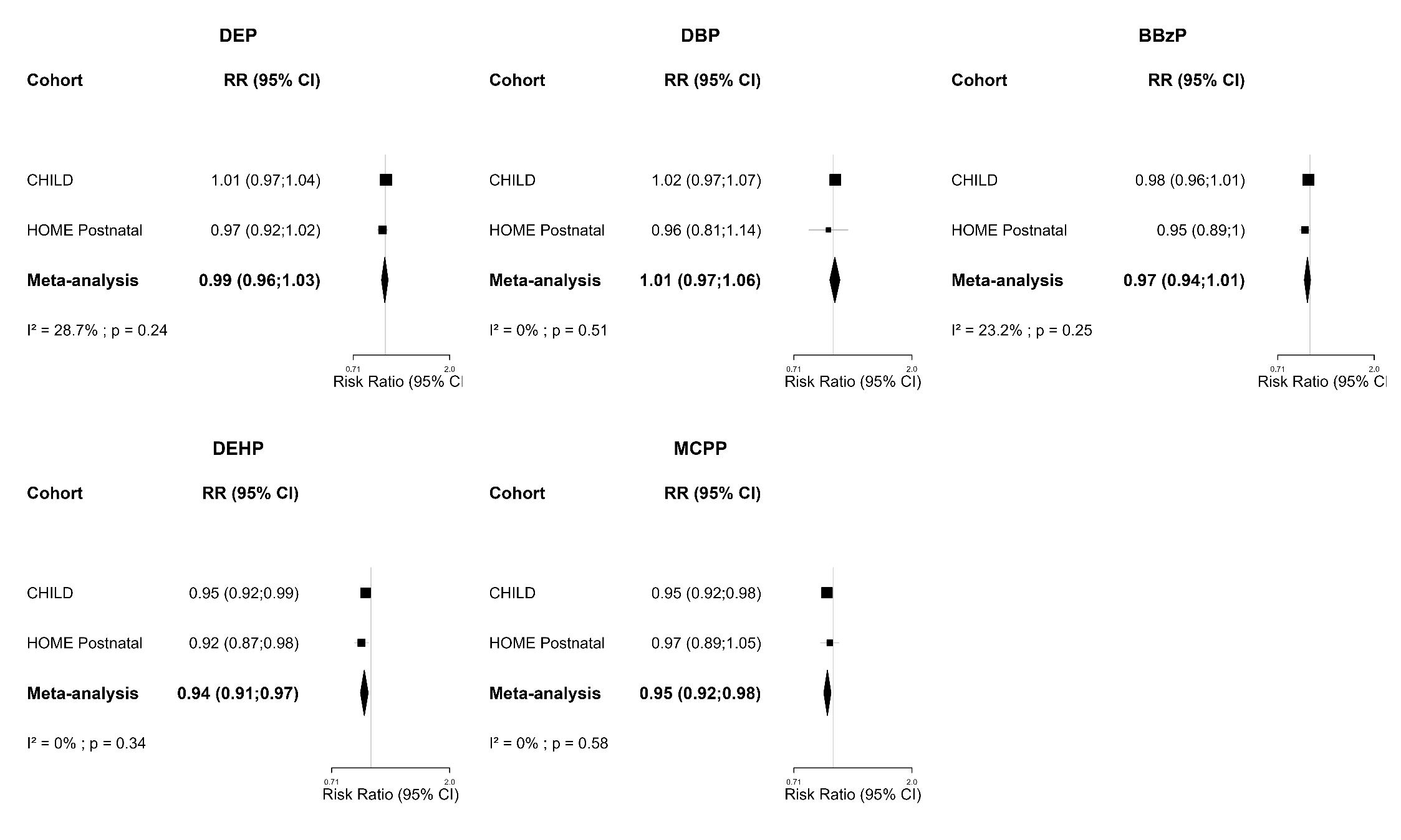
Figure S20. Random-effects Meta-analysis of Cohort-specific Estimates of postnatal Phthalate and Bisphenol Exposure and Childhood Eczema

*Models adjusted for maternal age, ethnicity, parental education, lone parenthood, family history of asthma, sex, prenatal tobacco smoke exposure, and season of birth. All exposures are modelled as estimated daily intakes, except MCPP, which is modelled using biomarker concentrations.* Abbreviations: BBzP: Benzyl butyl phthalate; DBP: Dibutyl phthalate; DEHP: Di(2-ethylhexyl) phthalate; DEP: Diethyl phthalate; MCPP: Mono-(3-carboxypropyl) phthalate; RR: Risk ratio

#
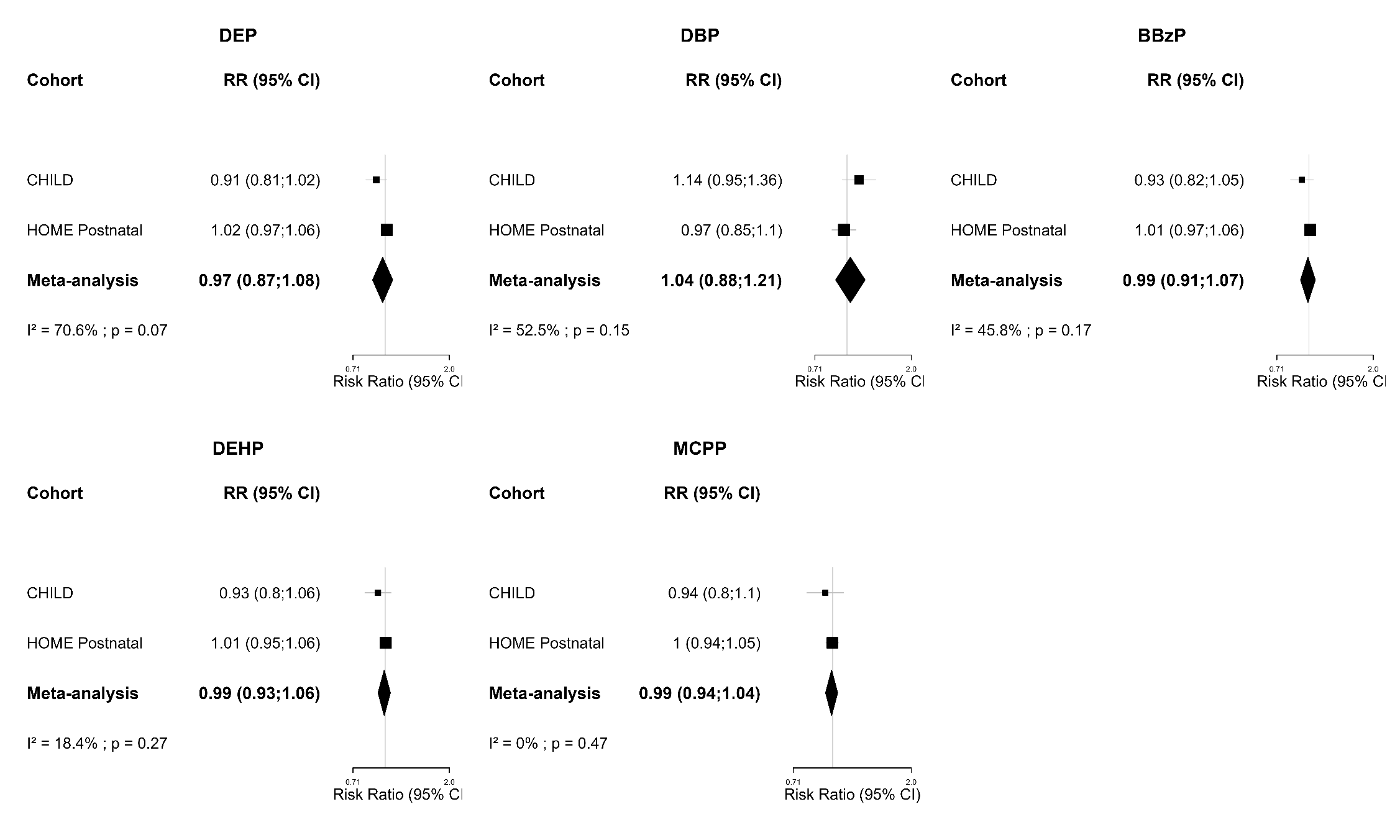
Figure S21. Random-effects Meta-analysis of Cohort-specific Estimates of postnatal Phthalate and Bisphenol Exposure and Childhood Rhinitis

*Models adjusted for maternal age, ethnicity, parental education, lone parenthood, family history of asthma, sex, prenatal tobacco smoke exposure, and season of birth. All exposures are modelled as estimated daily intakes, except MCPP, which is modelled using biomarker concentrations. Abbreviations: BBzP: Benzyl butyl phthalate; DBP: Dibutyl phthalate; DEHP: Di(2-ethylhexyl) phthalate; DEP: Diethyl phthalate; MCPP: Mono-(3-carboxypropyl) phthalate*; RR: Risk ratio

#
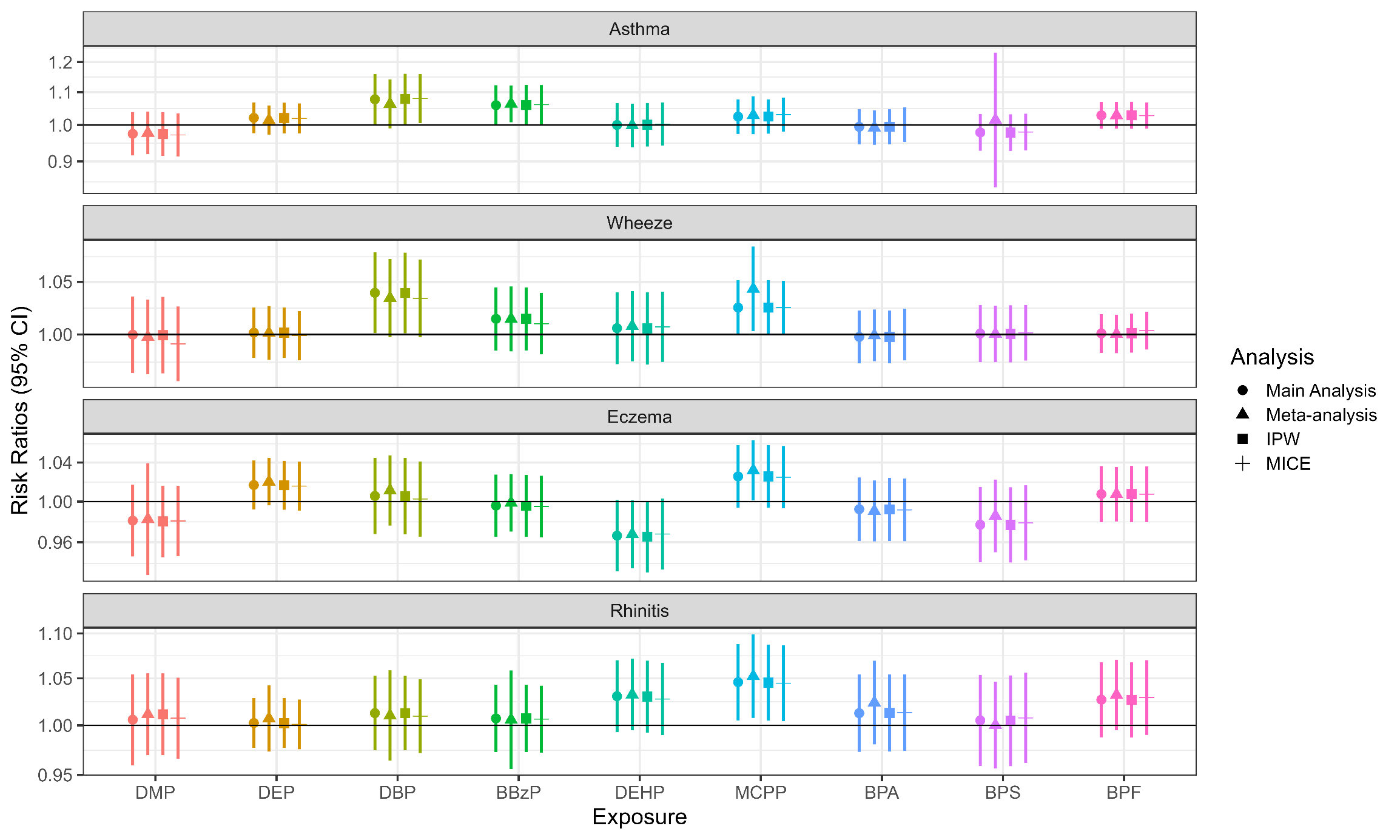
Figure S22. Forest plot of risk ratios with 95%confidence intervals of sensitivity analyses for prenatal exposure.

*Models adjusted for maternal age, ethnicity, parental education, lone parenthood, family history of asthma, sex, prenatal tobacco smoke exposure, and season of birth. All exposures are modelled as estimated daily intakes, except MCPP, which is modelled using biomarker concentrations. Abbreviations: BBzP: Benzyl butyl phthalate; BPA: Bisphenol A; BPF: Bisphenol F; BPS: Bisphenol S; DBP: Dibutyl phthalate; DEHP: Di(2-ethylhexyl) phthalate; DEP: Diethyl phthalate; DMP: Dimethyl phthalate; MCPP: Mono-(3-carboxypropyl) phthalate*

#
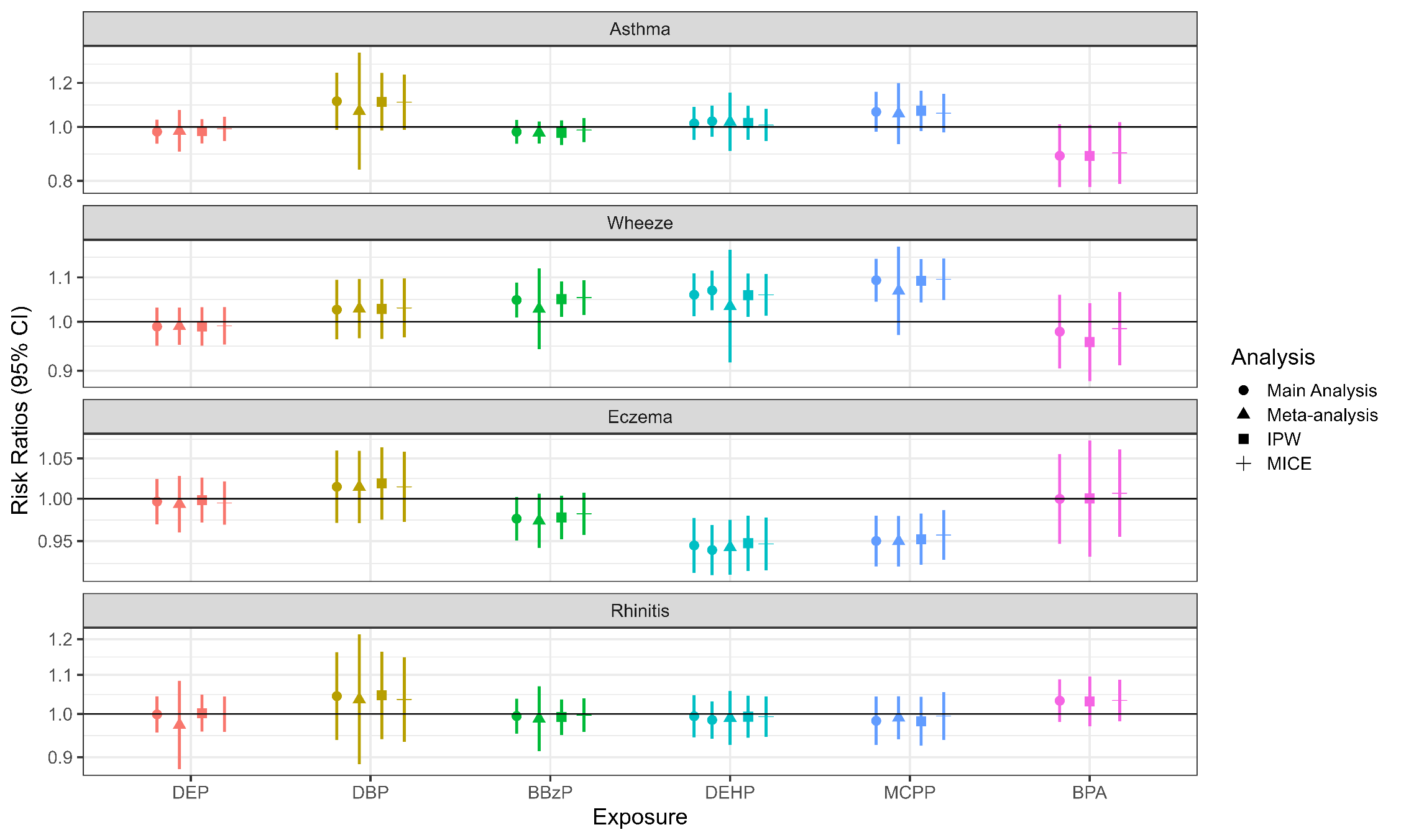
Figure S23. Forest plot of risk ratios with 95%confidence intervals of sensitivity analyses for postnatal exposure.

*Models adjusted for maternal age, ethnicity, parental education, lone parenthood, family history of asthma, sex, prenatal tobacco smoke exposure, and season of birth. All exposures are modelled as estimated daily intakes, except MCPP, which is modelled using biomarker concentrations.* Abbreviations: BBzP: Benzyl butyl phthalate; BPA: Bisphenol A; DBP: Dibutyl phthalate; DEHP: Di(2-ethylhexyl) phthalate; DEP: Diethyl phthalate; MCPP: Mono-(3-carboxypropyl) phthalate
